# Base-Mediated Depolymerization of Amine-Cured Epoxy Resins

**DOI:** 10.1021/acssuschemeng.3c04181

**Published:** 2023-11-20

**Authors:** Rebecca C. DiPucchio, Katherine R. Stevenson, Ciaran W. Lahive, William E. Michener, Gregg T. Beckham

**Affiliations:** Renewable Resources and Enabling Sciences Center, National Renewable Energy Laboratory, Golden, Colorado 80401 United States

**Keywords:** amine-cured epoxies, chemical recycling, C−O bond cleavage, C−N bond cleavage, base-mediated deconstruction, model polymers, model substrates

## Abstract

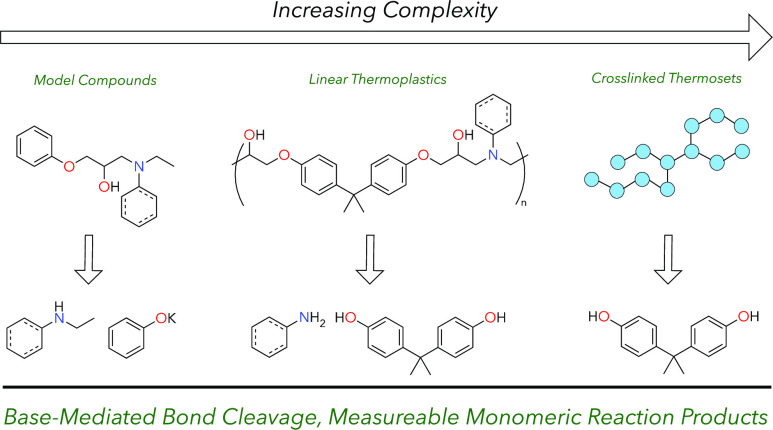

Carbon fiber-reinforced epoxy composites are used in multiple industries, including aerospace, automotive, and wind energy applications, due to their excellent strength-to-weight ratios and tunable material properties. Fortunately, recycling strategies for carbon fiber-based composites are emerging, with the primary focus on the recovery of fibers due to the cost and energy intensity in their production. In addition to fiber recovery, there is an opportunity to recycle the epoxy components such that ideal recycling strategies would yield both fibers and epoxy monomers for reuse. To that end, here we examine potassium *tert*-butoxide-mediated cleavage of C–O and C–N bonds in amine-cured epoxy resins. We accomplish this via developing model compounds that reflect both C–O and C–N linkages in amine-cured epoxy composites before expanding to both model linear thermoplastics and thermosets. We obtain excellent yields of both phenol (up to 97% molar yield) and amine products (up to 99 mol %) from aromatic and/or aliphatic amine-based model compounds. This system enables up to a quantitative yield of bisphenol A and up to 58% molar yield of aniline from model thermoplastic epoxy amines and 71% molar yield of BPA from a reaction with a thermoset substrate. These data correspond to a 15% mass recovery of BPA from a commercial epoxy thermoset.

## Abstract 2

This paper illustrates a base-mediated approach for epoxy resin deconstruction, with increased complexity from model compounds to industrially relevant thermosets. Work here will inform improving epoxy recycling strategies to tackle growing composite waste.

## Introduction

Carbon fiber-reinforced epoxy composites are used as lightweight components in a variety of products ranging from wind turbine blades to structural components in airplanes and vehicles.^1^ Carbon fiber costs range from $20/kg to $40/kg for lower strength applications or up to $175/kg for aviation-specific applications.^2,3^ Production of these materials requires substantial energy inputs, which in turn generates 43 kg of CO_2_e/kg of new fiber during typical US carbon fiber manufacturing.^2,4,5^ The substantial energy and GHG emissions associated with carbon fiber production and the lack of recycling options have motivated the development of strategies for carbon fiber recovery. Meanwhile, epoxy resins comprise about ∼50 wt % of carbon fiber composite materials, and global demand for these resins was 4 million metric tons in 2020.^2,6^ Epoxies on their own are primarily used in construction, coatings, and electronics. Production of epoxy thermosets results in 4.6 CO_2_e/kg of new resin.^2^

Current disposal of composite waste usually involves landfilling,^1^ pyrolysis,^7^ or grinding composites for use in applications that can tolerate lower-quality mechanical properties.^8,9^ Emerging chemical recycling strategies to deconstruct composite waste materials have been reported using Lewis acidic,^10−12^ strong Bronsted acid,^13^ oxidative,^14−16^ or ionic liquid-based^17^ reactions. A relatively new contribution employs a homogeneous Ru-based catalyst.^18^ Work to date has primarily focused on recycling carbon fiber, including assessment of postrecovery mechanical properties of the fibers. However, the epoxy portion of these composites also represents a substantial amount of unrecovered carbon, encouraging opportunities to recover epoxy monomers while still maintaining carbon fiber strength and alignment.^2^

**Figure 1:**
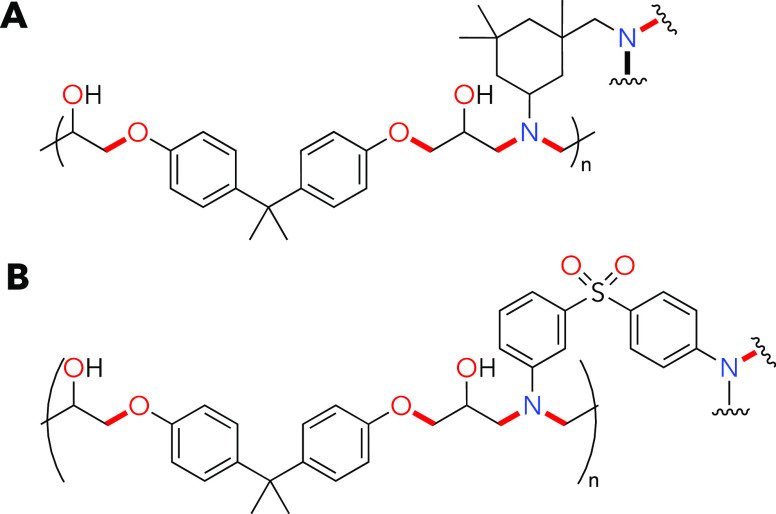
General examples of epoxy resin structures. (A) Aliphatic epoxy amines and (B) aromatic epoxy amine networks. In all cases, bonds targeted for cleavage are highlighted in red.

To date, chemical recycling strategies specific to amine-cured resins are primarily limited to strong acids or oxidants.^19^ Amine-cured epoxies represent the epoxy component of some of the strongest and more widely used composite materials.^6^ These resins are more challenging to depolymerize relative to their anhydride-cured material counterparts, the latter of which are linked via ester bonds, for which a variety of catalytic strategies exist.^20^ Conversely, amine–epoxy systems contain ether and amine linkages between monomers (Figure 1).^21^ Using tetrafunctional amine-based curing agents also results in a more densely cross-linked network than difunctional alcohol monomers. In addition, these epoxies are often proprietary structures that vary in composition based on the intended application. As a result, literature contributions in epoxy recycling tend to either use industry samples or generate their own cross-linked networks.^10,11,13,14,22^ Overall, efforts in epoxy recycling would benefit from improved substrate characterization such that deconstruction reactions can be better understood and the development of more efficient depolymerization processes will be accelerated.^23^

Chemical recycling methods for amine-cured epoxy resins and carbon fiber composites should ideally cleave C–O or C–N bonds to generate useful monomers. With this motivation, here we report a potassium *tert*-butoxide (KO*t*Bu)-mediated strategy for the depolymerization of amine-cured epoxy resins developed via reactions with aromatic and aliphatic amine-based model compounds. Using metal alkoxide bases as reagents for deconstruction resulted in valuable monomer products from simultaneous C–O and C–N cleavage, and these reagents also play a key role in maintaining high monomer yields. Reactions with model epoxy thermoplastics were used to inform reaction conditions on both model and industrially motivated amine-cured epoxy thermosets. The bottom-up approach in this work also presents well-characterized model substrates of multiple complexities for depolymerization studies.

## Results

**Figure 2:**
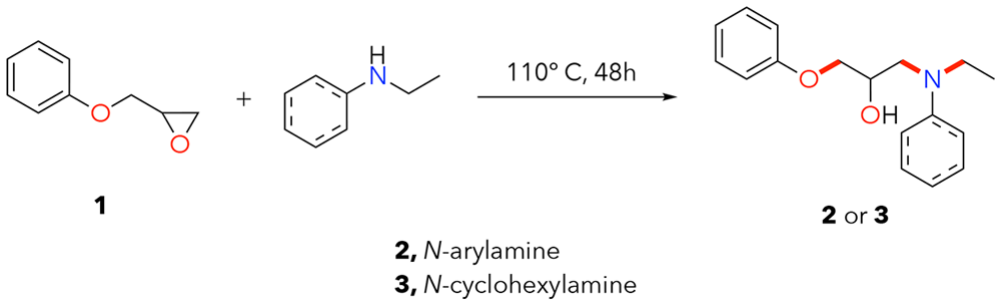
Synthetic routes to (**2**) aromatic and (**3**) aliphatic epoxy model compounds. Bonds targeted for cleavage are highlighted in red.

We initially designed small molecules **2** and **3** (Figure 2), to act as model systems to reflect the C–O ethereal and C–N amino linkages in industrial epoxy amine materials. These model compounds allowed for the screening of reaction conditions for deconstruction and simple postreaction analysis. These small-molecule models are straightforward to synthesize, here up to 50 g in a single batch, by combining epoxide **1** with a chosen amine partner and heating the mixture to 110 °C in the absence of a solvent. The resulting products were not purified before use in deconstruction after confirming purity by nuclear magnetic resonance (NMR) and gas chromatography (GC) (see Figures S1–S8 in the Supporting Information (SI) for model compound characterization data, Section S1 for materials and instrumentation, and Section S2 for the model compound syntheses). Starting materials and deconstruction products were quantified in a single GC experiment (see Section S3 and Figure S9 for GC method development information).

We began testing deconstruction conditions by combining our aromatic model compound **2** with a variety of oxidants and Lewis acids. These conditions were inspired by recently published work from our group that highlighted oxidative C–C cleavage in other commodity plastics.^24^ These conditions all resulted in very low product yields and/or instability of most identified amine reaction products (Section S4). We moved on to homogeneous reducing agents such as LiAlH_4_ or NaBH_4_, and no reactivity was observed in either case. Further screens shifted to 5 equiv each of a wide range of bases from different reactivity classes. We evaluated bases KO*t*Bu, Cs_2_CO_3_, KOH, diazabicycloundecene (DBU), and sodium bis(trimethylsilyl)amide (NaHMDS). Trial solvents for these base-mediated tests were toluene or ethylene glycol, as we hypothesized that bases would react well in these solvents, and high boiling points would accommodate relatively high temperatures for screening. This was important as we inevitably needed to heat polymers above their *T*_g_ for reactivity in the later stages of this paper. The screening reaction temperature was 140 °C such that toluene reactions would be at reflux, and the ethylene glycol counterparts would not. Reactions were conducted in microwave vials that can withstand up to 30 bar of pressure (see Section S5 in the SI for general deconstruction methods). We qualitatively assessed the reactivity for productive reactions by GC with a flame ionization detector (FID) and identified products by a combination of commercial standards, mass spectrometry, and nuclear magnetic resonance (NMR) spectroscopy analyses of isolated small molecules. We then quantitatively measured product yields for two preferred initial base classes with GC-FID via calibration curves and continued to optimize conditions below for promising bases.

**Figure 3:**
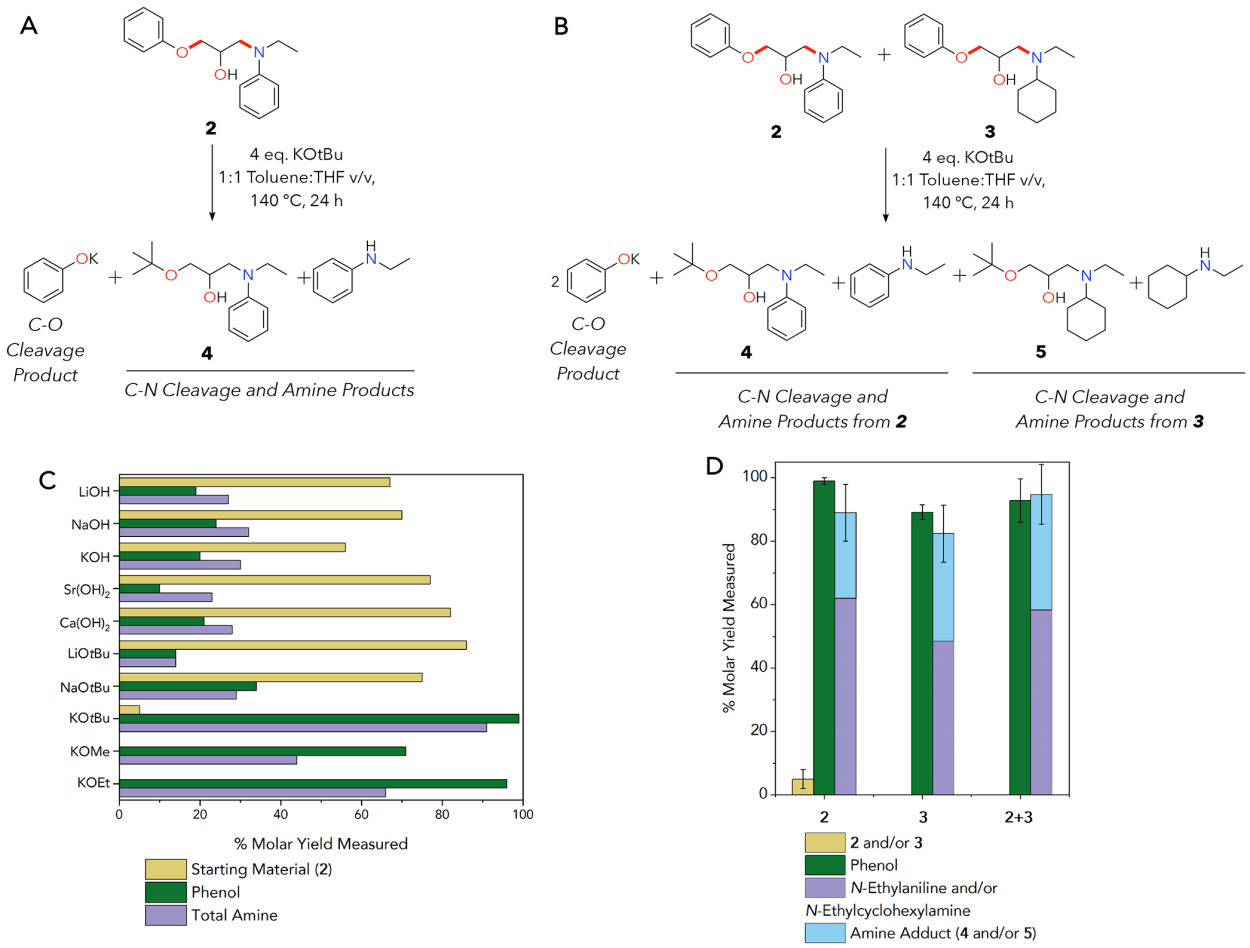
(A) Reaction scheme for C–O and C–N bond cleavage in an aromatic (**2**) amine-based model compound. Reaction conditions are listed to the right of the arrow (4 equiv KO*t*Bu, 24 h at 140 °C, 1:1 THF:toluene, 3 mL), bonds targeted for cleavage are shown in the starting materials in red, and C–O and C–N bonds are distinguished. (B) Reaction scheme for C–O and C–N bond cleavage in aromatic (**2**) and aliphatic (**3**) amine-based model compounds. Reaction conditions are listed to the right of the arrow (4 equiv KO*t*Bu, 24 h at 140 °C, 1:1 THF:toluene, 3 mL), bonds targeted for cleavage are shown in the starting materials in red, and C–O and C–N bonds are distinguished. Note that schemes in **A** and **B** reflect identified and quantified products only. (C) Optimizing base choice for C–O and C–N bond cleavage. (D) Optimized reaction conditions as in **A** and product yields for both these model compound systems. As noted in the text, potassium phenoxide is generated in situ but is protonated during the reaction workup and thus was measured and quantified as phenol. These experiments were conducted in duplicate, and the error bars reflect standard deviation values. Numerical data for **D** are provided in Table S8.

After these initial reaction screens, only conditions in toluene generated any product, and only KO*t*Bu resulted in full consumption of **2**. As shown in Figure 3A, the reaction products were divided into potassium phenoxide as the sole C–O cleavage product and a combination of *N*-ethylaniline and amine adducts **4** as the only two identifiable amine products from **2** (Figure 3A). Figure 3B illustrates reaction products from **2** and **3** together, which are elaborated on later in this section. Characterization data after isolation of amine adducts **4** and **5** (Figures S17–S24) confirmed both of their structures, allowing us to determine the yields of these new compounds. Molar yields shown in Figure 3D reflect this separation of alcohols from C–O cleavage and all amine products when applicable.

After observing that the potassium phenoxide generated from C–O cleavage under highly basic conditions (pH ≈ 14) was not fully soluble in toluene, we transitioned the reaction solvent to a 1:1 v/v mixture of toluene:tetrahydrofuran (THF). This solvent mixture was also found to be useful for enabling polymer reactivity (vide infra). For the model compounds, a 1:1 v/v mixture of toluene and THF allowed for direct sampling and yield measurements without any reaction quenching or workup needed. The potassium phenoxide could be measured and quantified with a phenol standard as this salt becomes protonated during dilution before GC analyses to form phenol. KO*t*Bu also generates higher yields than either sodium or lithium *tert*-butoxide (Figures 3C and S12 and Table S4). This idea of counterion noninnocence is well established in organometallic chemistry and catalysis, as these changes are often responsible for either differences in reactivity or product selectivity.^25−28^

When further comparing the KO*t*Bu-mediated reactivity with five different metal hydroxide bases (LiOH, NaOH, KOH, Ca(OH)_2_, and Sr(OH)_2_), KO*t*Bu exhibited improved reactivity in all cases (Figures 3C and S10 and Table S2). All reactions using each of the five hydroxide bases resulted in significant starting material remaining after 24 h at 140 °C, relative to full consumption using KO*t*Bu under the same conditions. Similarly, either KOEt or KOMe generated full conversion of **2** to reaction products (Figures 3C and S11 and Table S3). Through additional reaction screening, we continued with ^–^O*t*Bu bases instead of their methyl or ethyl counterparts due to similar reaction profiles but either fewer reaction side products, improved reagent robustness, and/or lower cost with its use (Figures 3C and S11 and Table S3).

Additional experimental trials investigated reaction temperatures, base equivalents, reaction volume, and reaction time (Figures S13 and S15 and Tables S5–S7). Optimized yields for bond cleavage with **2** were 99 ± 1 mol % for phenol and 89 ± 9 mol % for total amine products (Figure 3D, reaction conditions: 140 °C, 24 h, 1:1 THF:toluene, 4 equiv of KO*t*Bu). These reaction conditions worked similarly well with **3** alone (89 ± 3 mol % for phenol, 85 ± 5 mol % for total amine products, Figure 3D). We also mixed equal ratios of **2** and **3** as a final model compound test to highlight the ability of KO*t*Bu to cleave C–N and C–O bonds in a combination of aliphatic or aromatic amine-containing materials. In this case, the total phenol yield was 97 ± 4 mol % and the total yield of amine products was 99 ± 1 mol % (Figure 3D).

**Figure 4:**
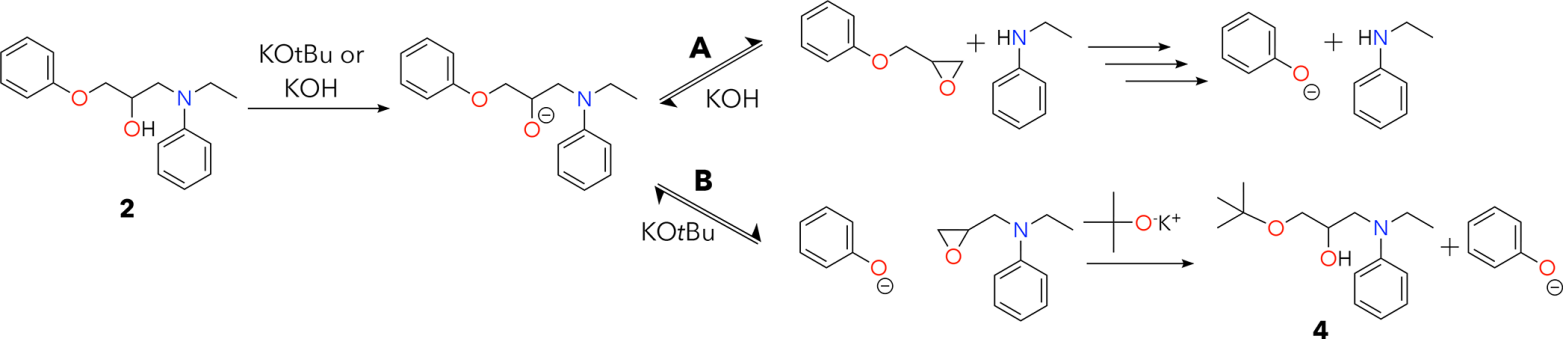
Proposed reaction pathway for C–O and C–N bond cleavage in epoxy model compounds. As noted in the text, we identified that only alkoxide bases can access pathway B, allowing for improved yields with these reagents. In this figure, we used KO*t*Bu as an example of an alkoxide base and KOH to represent other base classes.

Based on the products formed during the reaction, we hypothesized that these KO*t*Bu-mediated bond cleavage events occur via an epoxide mechanism with two potential reaction pathways (Figure 4) that occur simultaneously. Both options generate potassium phenoxide, but pathway **A** results in free *N*-ethylaniline or *N*-ethylcyclohexylamine, while pathway **B** yields amine adducts **4** or **5** depending on the starting material. Existing basic epoxy deconstruction strategies^16,22^ use hydroxide-type bases that can only access pathway **A**, while we hypothesize that reactivity through **A** and **B** concurrently leads to improved yield and stability of amine products. We assessed the validity of pathway **A** in this proposed mechanism by treating hypothesized intermediate epoxide **1** with combinations of KO*t*Bu, heat, and *N*-ethylaniline to observe product formation. Attempts to isolate intermediates used to test pathway **B** were successful in very low yields, and the desired intermediate presented decomposition challenges such that tests with it were not viable. All reactions in this test with **A** occurred at 24 h at 140 °C with 1:1 v/v THF:toluene. In these control reactions, **1** did not change with heat alone and cleanly generated potassium phenoxide after adding 2 equiv of KO*t*Bu. When combining **1** and 1 equiv of *N*-ethylaniline, both compounds were unchanged with heat alone, while adding 2 equiv of KO*t*Bu produced both phenoxide and *N*-ethylaniline (Figure S26). We hypothesize that these compounds are stable and do not combine to form **2** at 140 °C in this test because they are in a dilute solution instead of being mixed neatly (which is how we generate model compound **2**). These data are in alignment with the hypothesized reaction pathway shown in Figure 4.

**Figure 5:**
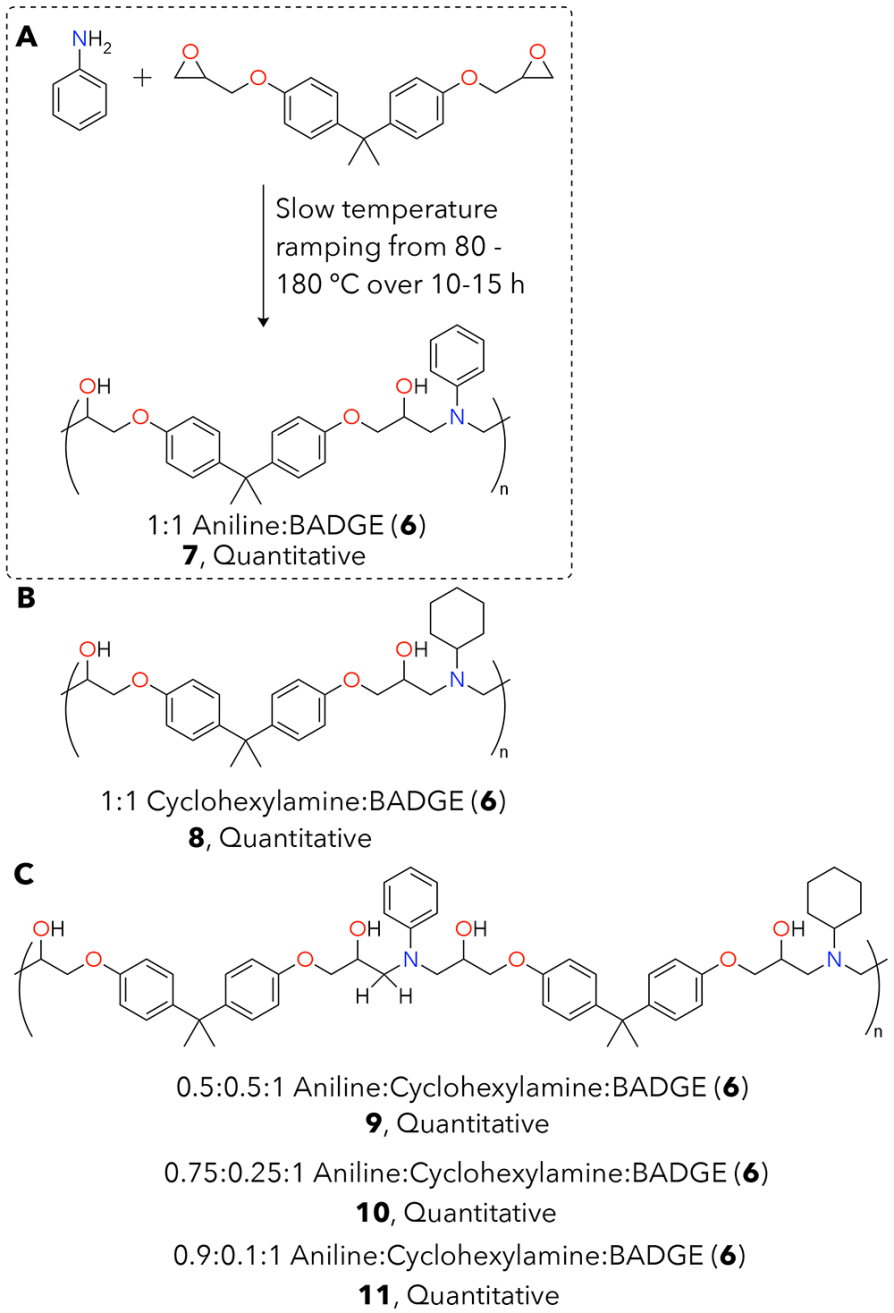
(A) Synthesis of **7** as an example aromatic thermoplastic reaction scheme, (B) an aliphatic thermoplastic, **8**, and (C) a 50:50 mixed aromatic:aliphatic thermoplastic substrate, **9**. All polymers are BADGE-based, and **7** contains only an aromatic amine comonomer agent while **8** uses an aliphatic amine and **9** contains a 1:1 ratio of these monomers. All structures in this figure were polymerized in silicone trays in a variable-temperature oven and used directly without any purification.

Based on the success with model compounds, we generated related thermoplastics to investigate reactivity with amine-cured epoxy polymers. We used 2,2′-(((propane-2,2-diylbis(4,1-phenylene))bis(oxy))bis(methylene))bis(oxirane) (BADGE, **6**) since it is a common industrial epoxide in amine–epoxy resins (Figure 5).^6,29^ Thermoplastics combine aniline and/or cyclohexylamine in 1:1 molar ratios with **6** as a diepoxide. All polymers in this work are generated via polymerizing resin mixtures in silicone trays containing 1 cm^3^ cavities (Figure S25). Resultant polymer substrates are 1 cm × 1 cm pieces of ∼200 mg each, where one cube is used for each deconstruction reaction test. MAss is used to describe these polymer pieces, as the 1 × 1 × 1 silicone cavities were intentionally not filled all the way. As with the small-molecule models, polymer yields were quantitative, and resultant materials allow us to separate the reactivity of aromatic and aliphatic amines in depolymerization (**7** and **8**). We also extended our epoxy synthesis strategy to a mixed aliphatic and aromatic thermoplastic containing 1:1 aniline:cyclohexylamine (**9**). We report full heating cycles for each of these polymers in the SI, as developed based on polymerization data from differential scanning calorimetry (DSC; Figures S30A, S35A, S41A, S46A, and S51A). Polymer samples that are predominantly aromatic amine (i.e., **7**) based are darker red-brown in color, while those with significant aliphatic amine content are yellow (**8** and **9**).

We also used these linear polymers to enable solution-state analysis techniques before and after depolymerization reactions including NMR spectroscopy and gel permeation chromatography (GPC). Figures S26–29, S31–34, and S37–40 show ^1^H, ^13^C, correlation spectroscopy (COSY), and heteronuclear single quantum coherence (HSQC), while Figures S30F, S35E, and S41E show infrared (IR) spectra for each compound. GPC was also used to determine molecular weight distributions for soluble polymer fractions (Figures S30E, S35D, S41D). We supplemented these solution-state structural data with thermal information from DSC (Figures S30, S35, and S41) and thermogravimetric analysis (TGA) for each polymer (Figures S30, S35, S41).

We also completed solubility tests with polymers **7**–**9** (Figures S52–S54), which demonstrated that all 3 polymers are insoluble in toluene, trichlorobenzene, ethylene glycol, and limonene. Additionally, **7** was also insoluble in acetic acid, hexafluoroisopropanol, and *N-*methyl-2-pyrrolidone. In contrast, we obtained the best results in all cases with ethers, such as THF or dioxane. Through structural analyses and solubility studies for each thermoplastic, we hypothesized that aromatic amine-containing materials are slightly cross-linked through the alcohol in the polymer backbone; an elaboration on this proposed mechanism and resultant network is shown in Figure S56. We anticipate that this cross-linking occurs only with materials that have at least 75% aniline content due to the decreased nucleophilicity of aniline compared with cyclohexylamine. As a result, polymers with 75 or 90% aniline content (**10** and **11**, Figure 5C) remain partly insoluble in all 10 organic solvents tested with polymer **7** (Figure S52), indicating partial cross-linking (Figure S36). Solution-state analyses for these polymers reflect the ∼50% soluble polymer fraction (42% for **7** and 43% for **10**) for these slightly cross-linked thermoplastics (Figures S42–S45, S46D, S47–S50, S51D), while thermal and IR data are reflective of the whole polymer systems (Figures S46/S51).

**Figure 6:**
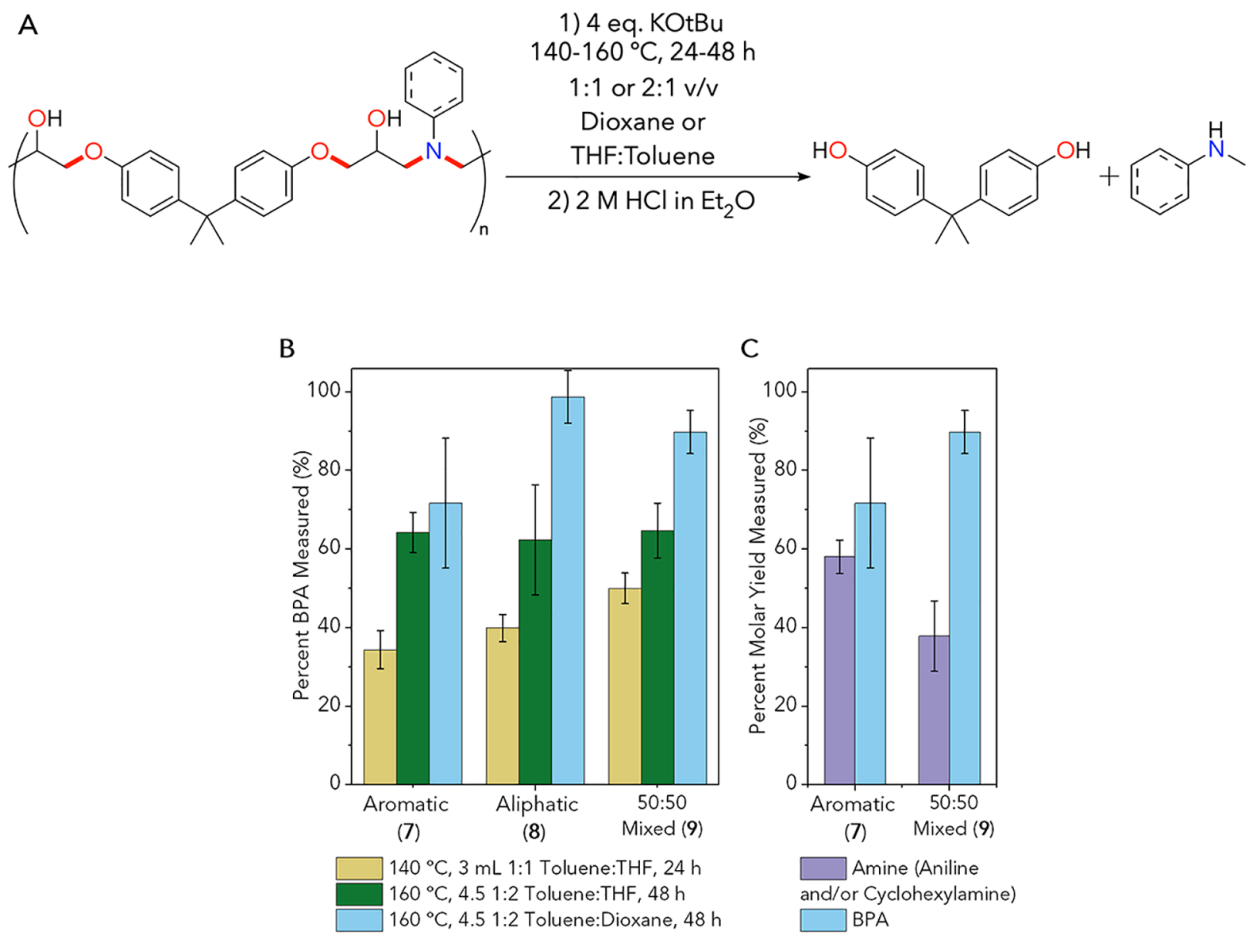
(A) Sample thermoplastic deconstruction reaction. Note that this scheme reflects identified and quantified products only. (B) Thermoplastic deconstruction BPA molar yields under three different conditions. Yellow bars (*left*) represent optimized reaction conditions from model compounds (140 °C, 24 h, 1:1 v/v THF:toluene), while the dark green bars (*middle*) represent partially optimized reaction conditions for thermoplastics (160 °C, 48 h, 2:1 v/v THF:toluene). Optimal conditions are shown in light blue (*right*, 160 °C, 48 h, 2:1 v/v dioxane:toluene). Reactions were conducted in duplicate or triplicate, and error bars show standard deviation values. (C) Amine (purple) and BPA (blue) yields from deconstruction of aromatic amine-containing thermoplastic substrates. Numerical data shown in this figure are provided in Tables S18 and S19.

The initial investigations into thermoplastic depolymerization utilized the optimized conditions from both model compounds **2** and **3** (140 °C, 24 h, 1:1 toluene:THF, see Section S8 for full thermoplastic deconstruction procedures). These reactions initially generate the dipotassium salt of bisphenol A (BPA). Upon reaction quenching with 2 equiv of 2 M HCl in ether (step 2 in Figure 6A), we directly obtained and quantified BPA (32 ± 8 mol % with **7**). We confirmed the initial dipotassium salt mentioned above via a BPA stability test, where 0.5 mmol of this diol was added and reacted alone under basic reaction conditions above (140 °C, 24 h, 1:1 toluene:THF). We measured only 46% of BPA before a 2 M HCl quench in this stability control reaction but observed 96% directly after this step. As a result, all remaining yields in this work are shown after a HCl quenching step. Reactions to optimize thermoplastic deconstruction illustrated that polymer dissolution or swelling must occur before bond cleavage events. As a result, reactions in toluene only were completely unsuccessful, since all polymers (**7**–**9**) would not dissolve and thus not depolymerize.

We subsequently improved our polymer deconstruction conditions through a 2:1 v/v THF:toluene ratio, maintaining a small amount of toluene for improved base reactivity, and added THF for polymer solubility (Figures S55/S56 and Tables S10/S11). We observed a large increase in BPA yields (36 to 43 mol % with **8** as a substrate) after 48 h reaction times (Figures S57/S58 and Tables S12/13). Additional data in the SI (Figure S59 and Table S14) highlight that using cryomilled thermoplastics results in higher yields than reacting unmodified cubes, presumably due to improved surface area. We recognize that this preprocessing strategy is impractical with carbon fiber-reinforced composites, so we continued with full cubes for reactions. Further optimization relative to small-molecule conditions raised the reaction temperature from 140 to 160 °C, with corresponding yield increases with **8** from 37 to 50 mol % (Figure S60 and Table S15). Combining all of these improvements produced a 66 ± 5 mol % BPA yield from **7** (Figure 6B and Table S17).

We proposed that a solvent to maintain polymer substrate solubility during reactions with a boiling point higher than that of THF might improve monomer yields. To this point, we substituted 3 mL of THF with dioxane in deconstruction reactions to obtain the highest amounts of BPA for all three thermoplastics. Both THF and dioxane result in similar qualitative solubilities of the starting polymers at room temperature. We propose that improved reactivities are due to the higher boiling point of dioxane as compared to that of THF such that a larger proportion of this solvent was in solution at 160 °C to solubilize polymers during deconstruction. The resultant molar yields of BPA were 72 ± 16% for **7**, 99 ± 7% for **8**, and 90 ± 5% for **9**. These data did not significantly improve after increasing the reaction temperature to 180 °C (Figure S61 and Table S16). Our BPA yields from thermoplastics compare with the high yields of phenol observed when reacting small-molecule models (vide supra). Further, our best reaction conditions for thermoplastics also resulted in significant yields of aniline after reactions (58 ± 4 mol % from **7** and 38 ± 9 mol % of total amine content from **9**; Figure 6C). Aliphatic amine components of these polymer reactions are complex oligomers and vary much more than in the small-molecule reactions; identifying and measuring these products are ongoing areas of work. Preliminary investigations to this goal used HPLC-MS data to assess the mixture of amine components; these were short oligomers that are very complex to assign or quantify.

**Figure 7:**
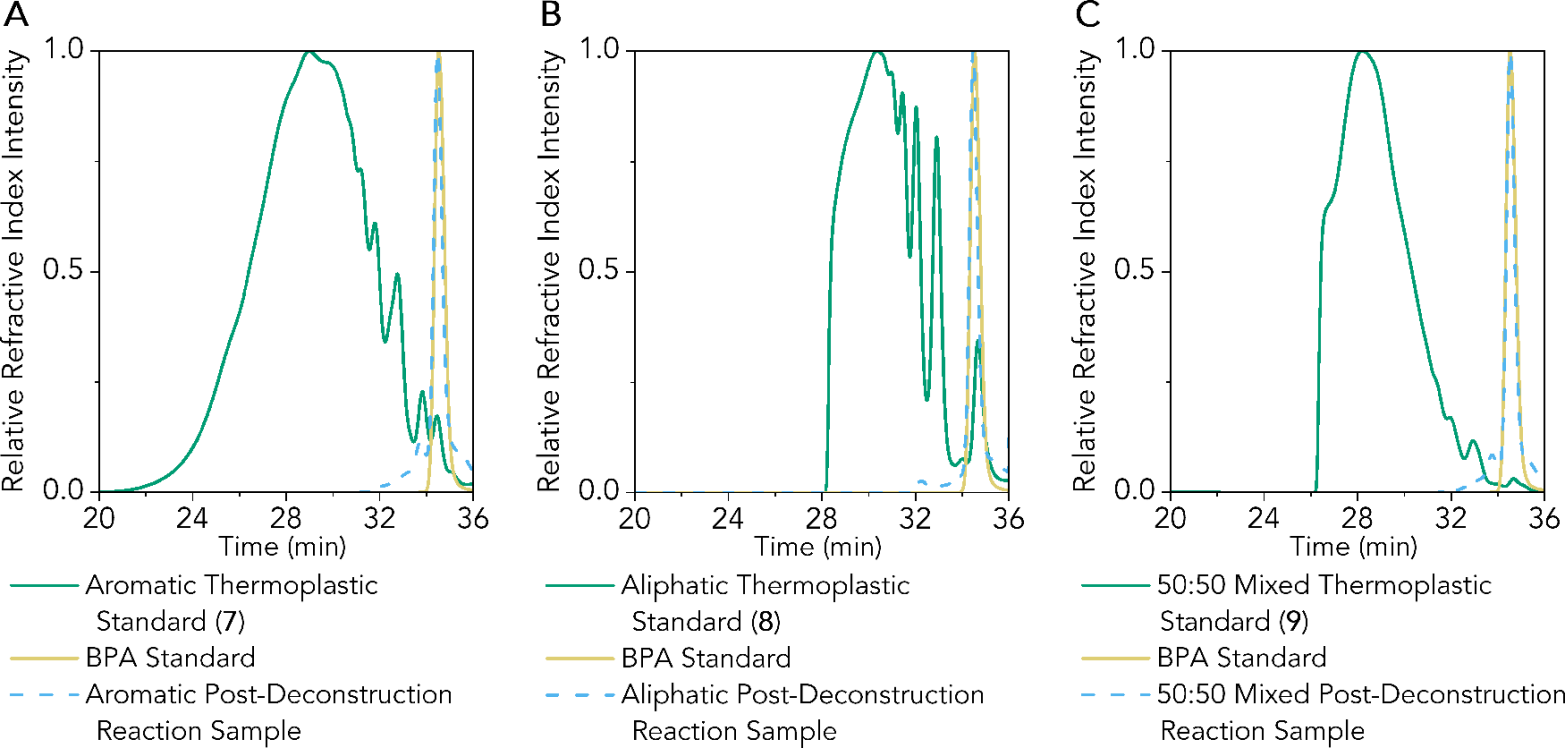
(A) GPC data before and after epoxy deconstruction for aromatic thermoplastic **7**, (B) aliphatic thermoplastic **8**, and (C) 50:50 mixed thermoplastic **9**. In all three cases, GPC experiments were conducted in THF. Dark green traces represent thermoplastic standards before reaction, yellow traces are from a BPA standard, and light blue traces represent postreaction analyses. The sharp peak observed in all three postreaction samples is BPA. Amine products cannot be observed by GPC because their molecular weights are below the instrument and column resolution. All plots are from the refractive index (RI) data. *M_n_* and PDI data for all polymers are in Table S9, where PDI represents polydispersity index information.

Optimized deconstruction results can also be visualized with GPC traces before and after reactions for models **7**–**9** (Figure 7). The difference in postdeconstruction reaction purity between THF and dioxane-containing reactions is also represented by GPC (Figure S62).

**Figure 8:**
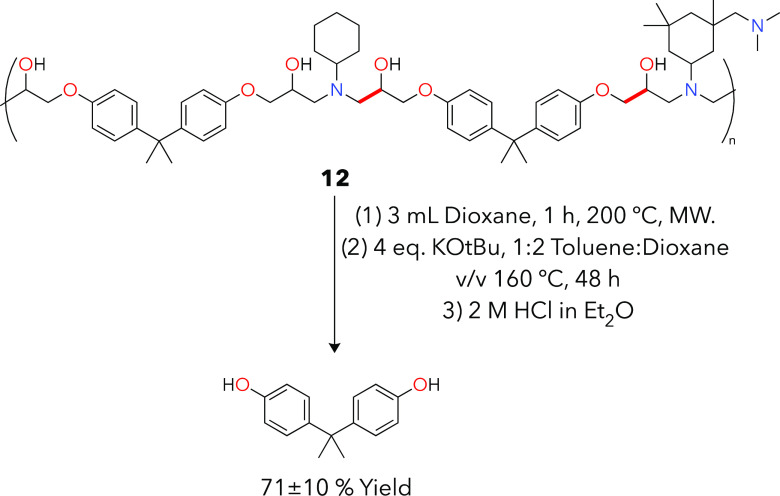
Deconstruction reaction using an aliphatic amine-based thermoset substrate (**12**). Reaction conditions 160 °C, 48 h, and 2:1 v/v THF:toluene were applied from thermoplastic depolymerization work. In this figure, the MW represents microwave irradiation. Raw data representing these yields are found in Table S19.

To test thermoset materials, we developed thermoset **12** (Figure 8) to replicate the thermal properties of industrial amine-epoxies that we measured from Hexion’s proprietary resin combinations (*T*_g_ = 85 °C). This process involved optimizing cross-link densities and ratios of monoamines to diamines in a polymer resin (see Section S9 for a full thermoset synthesis procedure). We chose isophorone diamine (IPDA) as a cross-linker due to its prevalence in current industrial epoxies.^6^ Combining this amine in equal ratios with cyclohexylamine and using **6** as the sole epoxide resulted in a polymer with a *T*_g_ of 105 °C (Figures 8 and S63).

Relative to the thermoplastics described above, thermoset **12** does not dissolve in any solvent, but rather, it only swelled slowly over time. Reactions with a thermoset cube and optimized conditions from thermoplastics above resulted in minimal product formation and a full cube remaining after heating. To accelerate thermoset swelling and thus improve depolymerization, we heated a cube of polymer **12** in 3 mL of either THF or dioxane for 1 h at 200 °C, taking inspiration from previous success with solvent choices when using thermoplastic substrates. These preswelling experiments were done in a CEM chemical microwave. After this, the cube already began to break apart into somewhat smaller and fragile, gel-like pieces but the network itself was unchanged via TGA analyses. We quantified this polymer swelling by TGA to assess solvent incorporation into the network by % mass (Figure S65A for postswelling TGA data).

We then transferred the CEM vial contents to a Biotage microwave vial with an appropriate pressure rating, added 1.5 mL of toluene and 4 equiv of KO*t*Bu to this postswelling mixture, and used optimized reaction conditions from thermoplastics (48 h, 160 °C) to deconstruct this polymer for a BPA yield of 71 ± 10 mol % when dioxane is used as a cosolvent. This compares with 43 ± 9 mol % BPA without preswelling. As with thermoplastics above, the aliphatic amine products were not observed after thermoset deconstruction. Addressing and understanding this challenge is a current area of investigation.

Finally, these results were expanded to an industrial material using EPIKOTE Resin MGS RIMR 135 and Curing Agent MGS RIMH134-RIMH-137. We combined and polymerized these reagents as instructed in the data sheet from Hexion to generate thermoset resin **13**. Thermal data for this polymer (*T*_g_ = 85 °C) is in Figure S64. Preswelling data for this polymer are shown in Figure S65B. Applying the optimized reaction conditions from **12** above (2:1 THF:tol, 100 wt % KO*t*Bu, 48 h, 160 °C) resulted in 15 wt % BPA by GC-FID from this industrial epoxy amine.

## Discussion

This work demonstrates a KO*t*Bu-mediated method for epoxy amine deconstruction developed by using synthesized model compounds, thermoplastics, and a thermoset. Full depolymerization of amine–epoxy resins will require C–O and/or C–N bond cleavage, and it is noteworthy that the monomers from epoxy amine resins pose unique stability challenges, especially as probable polyaniline formation is a common issue that prevents recovery in many oxidation or acid-catalyzed strategies.^12,14,15,29^ In our initial oxidation reaction tests, we obtained black and somewhat insoluble material after reactions. Confirming that the exact aggregate identity of this material is particularly challenging and was not considered part of this work. We propose that both amine and potassium phenoxide yields are consistently higher with alkoxide bases than any of their hydroxide counterparts because hydroxide bases consistently do not access products from the second reaction pathway in Figure 4. Isolating amine adducts **4** and **5** from aromatic or aliphatic amine models enables trapping amines through less reactive products in epoxy deconstruction. Future work aimed at recovering amine hardeners should investigate catalytic strategies that continue to stabilize these products for improved yields. Further, we highlighted reactivity using both aromatic and aliphatic amines, as industrial epoxies often contain custom ratios of different amine curing agents in proprietary ratios.^6^ It is crucial for a recycling method to address both of these amine groups for applicability across composite industries.

Linear thermoplastics are useful tools in method development for epoxy depolymerization because they allowed us to separate reactivity challenges using polymer substrates from mass transfer issues associated with insoluble thermosets. These polymers were also prepared to maintain solution-state analyses before and after deconstruction reactions. We directly measured BPA and aniline after thermoplastic depolymerization reactions, substantially improving the recovery of epoxy carbon content relative to existing options.^29^ This BPA and aniline content could be used to generate new BADGE to be used directly with recovered amine for newly synthesized resins or to manufacture plastics with more established chemical recycling pathways, such as polycarbonates.^30^ Versatility from products generated is important, as epoxy redesign work expands and primarily focuses on biobased epoxides beyond only **6**.^31^ Another emerging redesign approach involves Recylamine, a technology that employs reversible acetal groups to existing epoxy resins, such that they can be cleaved as desired after use.^32^

When considering thermoset reactivity, industrial thermosets we worked with often have glass transition temperatures around 110 °C, and thus, we designed **12** to be close to that range. The reactions with thermosets highlighted the need for swelling prior to deconstruction such that KO*t*Bu can effectively penetrate this connected network. Yields of these thermoset reactions can potentially be improved through reaction engineering, which will be pursued in future efforts. Ongoing work will also emphasize expanding to carbon fiber-containing composites to assess mechanical material properties after deconstruction.

## Conclusions

In summary, we developed an aliphatic base-mediated strategy for cleaving C–O ether and C–N amine linkages in epoxy resins. This method was developed with model compounds and expanded to linear thermoplastic polymers for high yields of BPA and aniline. Reactions with synthesized amine-cured epoxy thermoplastics then translate to a thermoset that can be deconstructed to generate high yields of BPA. This method points to a strategy for advancing carbon fiber recovery in the future with the added objective of maintaining significant carbon from the epoxy portion of composite materials.

## Supporting Information Available

Reaction procedures; reagent information; characterization data; control reactions; and tabulated yields (PDF)

## References

[ref1] Gopalraj S. K.; Kärki T. A review on the recycling of waste carbon fibre/glass fibre-reinforced composites: fibre recovery, properties and life-cycle analysis. SN Appl. Sci. 2020, 2, 433 10.1007/s42452-020-2195-4.

[ref2] Nicholson S. R.; Rorrer N. A.; Carpenter A. C.; Beckham G. T. Manufacturing energy and greenhouse gas emissions associated with plastics consumption. Joule 2021, 5, 673–686. 10.1016/j.joule.2020.12.027.

[ref3] Malveda M.; Sesto B.; Zhang V.; Passararat S. IHS Report on Carbon Fibers, 2022.

[ref4] Hanes R. J.; Carpenter A. Evaluating opprtunities to improve material and energy impacts in commodity supply chains. Env. Syst. Decis. 2017, 37, 6–12. 10.1007/s10669-016-9622-5.

[ref5] This number was generated via a tool called Materials Flows through Industry (MFI). The MFI tool is freely available to interested users on the NREL web site. Accounts can be created there to use the tool.

[ref6] Linak E.; Buchholz U.; Guan M.; Kishi A. IHS Report on Epoxy Resins, 2020.

[ref7] Naqvi S. R.; Prabhakara H. M.; Bramer E. A.; Dierkes W.; Akkerman R.; Brem G. A critical review on recycling of end-of-life carbon fibre/glass fibre reinforced composites waste using pyrolysis towards a circular economy. Resour. Conserv. Recycl. 2018, 136, 118–129. 10.1016/j.resconrec.2018.04.013.

[ref8] Liu P.; Barlow C. Y. Wind turbine blade waste in 2050. Waste Manage. 2017, 62, 229–240. 10.1016/j.wasman.2017.02.007. 28215972

[ref9] Cooperman A.; Eberle A.; Lantz E. Wind turbine blade material in the united states: quantities, costs, and end-of-life options. Resour. Conserv. Recycl. 2021, 168, 105439 10.1016/j.resconrec.2021.105439.

[ref10] Wang Y.; Cui X.; Ge H.; Yang Y.; Wang Y.; Zhang C.; Li J.; Deng T.; Qin Z.; Hou X. Chemical recycling of carbon fiber reinforced epoxy resin composites via selective cleavage of the carbon-nitrogen bond. ACS Sustainable Chem. Eng. 2015, 3, 3332–3337. 10.1021/acssuschemeng.5b00949.

[ref11] Liu T.; Zhang M.; Guo X.; Liu C.; Liu T.; Xin J.; Zhang J. Mild chemical recycling of aerospace fiber/epoxy composite wastes and utilization of the decomposed resin. Polym. Degrad. Stab. 2017, 139, 20–27. 10.1016/j.polymdegradstab.2017.03.017.

[ref12] Ma Y.; Navarro C. A.; Williams T. J.; Nutt S. R. Recovery and reuse of acid digested amine/epoxy-based composite matrices. Polym. Degrad. Stab. 2020, 175, 109125 10.1016/j.polymdegradstab.2020.109125.

[ref13] Jiang J.; Deng G.; Chen X.; Gao X.; Guo Q.; Xu C.; Zhou L. On the successful chemical recycling of carbon fiber/epoxy resin composites under the mild condition. Compos. Sci. Technol. 2017, 151, 243–251. 10.1016/j.compscitech.2017.08.007.

[ref14] Navarro C. A.; Kedzie E. A.; Ma Y.; Michael K. H.; Nutt S. R.; Williams T. J. Mechanism and catalysis of oxidative degradation of fiber-reinforced epoxy composites. Top. Catal. 2018, 61, 704–709. 10.1007/s11244-018-0917-2. 30288016 PMC6166884

[ref15] Lo J. N.; Nutt S. R.; Williams T. J. Recycling benzoxazine-epoxy composites via catalytic oxidation. ACS Sustainable Chem. Eng. 2018, 6, 7227–7231. 10.1021/acssuschemeng.8b01790.

[ref16] Ma Y.; Nutt S. Chemical treatment for recycling of amine/epoxy composites at atmospheric pressure. Polym. Degrad. Stab. 2018, 153, 307–317. 10.1016/j.polymdegradstab.2018.05.011.

[ref17] Pérez R. L.; Ayala C. E.; Opiri M. M.; Ezzir A.; Li G.; Warner I. M. Recycling thermoset epoxy resin using alkyl-methyl-imidazolium ionic liquids as green solvents. ACS Appl. Polym. Mater. 2021, 3, 5588–5595. 10.1021/acsapm.1c00896. 34796334 PMC8593865

[ref18] Ahrens A.; Bonde A.; Sun H.; Wittig N. K.; Hammershøj H. C. D.; Batista G. M. F.; Sommerfeldt A.; Frølich S.; Birkedal H.; Skrydstrup T. Catalytic disconnection of c–o bonds in epoxy resins and composites. Nature 2023, 617, 730–737. 10.1038/s41586-023-05944-6. 37100913 PMC10208972

[ref19] Navarro C. A.; Giffin C. R.; Zhang B.; Yu Z.; Nutt S. R.; Williams T. J. A structural chemistry look at composites recycling. Mater. Horiz. 2020, 7, 2479–2486. 10.1039/D0MH01085E.

[ref20] Li H.; Aguirre-Villegas H. A.; Allen R. D.; Bai X.; Benson C. H.; Beckham G. T.; Bradshaw S. L.; Brown J. L.; Brown R. C.; Cecon V. S.; Curley J. B.; Curtzwiler G. W.; Dong S.; Gaddameedi S.; García J. E.; Hermans I.; Kim M. S.; Ma J.; Mark L. O.; Mavrikakis M.; Olafasakin O. O.; Osswald T. A.; Papanikolaou K. G.; Radhakrishnan H.; Sanchez Castillo M. A.; Sánchez-Rivera K. L.; Tumu K. N.; Van Lehn R. C.; Vorst K. L.; Wright M. M.; Wu J.; Zavala V. M.; Zhou P.; Huber G. W. Expanding plastics recycling technologies: chemical aspects, technology status and challenges. Green Chem. 2022, 24, 8899–9002. 10.1039/D2GC02588D.

[ref21] Capricho J. C.; Fox B.; Hameed N. Multifunctionality in epoxy resins. Polym. Rev. 2020, 60, 1–41. 10.1080/15583724.2019.1650063.

[ref22] Zhao Q.; Jiang J.; Li C.; Li Y. Efficient recycling of carbon fibers from amine-cured cfrp composites under facile condition. Polym. Degrad. Stab. 2020, 179, 109268 10.1016/j.polymdegradstab.2020.109268.

[ref23] Ellis L. D.; Rorrer N. A.; Sullivan K. P.; Otto M.; McGeehan J. E.; Román-Leshkov Y.; Wierckx N.; Beckham G. T. Chemical and biological catalysis for plastics recycling and upcycling. Nat. Catal. 2021, 4, 539–556. 10.1038/s41929-021-00648-4.

[ref24] Sullivan K. P.; Werner A. Z.; Ramirez K. J.; Ellis L. D.; Bussard J. R.; Black B. A.; Brandner D. G.; Bratti F.; Buss B. L.; Dong X.; Haugen S. J.; Ingraham M. A.; Konev M. O.; Michener W. E.; Miscall J.; Pardo I.; Woodworth S. P.; Guss A. M.; Román-Leshkov Y.; Stahl S. S.; Beckham G. T. Mixed plastics waste valorization through tandem chemical oxidation and biological funneling. Science 2022, 378, 207–211. 10.1126/science.abo4626. 36227984

[ref25] Zhdanko A.; Maier M. E. Explanation of counterion effects in gold(i)-catalyzed hydroalkoxylation of alkynes. ACS Catal. 2014, 4, 2770–2775. 10.1021/cs500446d.

[ref26] Jia M.; Bandini M. Counterion effects in homogeneous gold catalysis. ACS Catal. 2015, 5, 1638–1652. 10.1021/cs501902v.

[ref27] Lu Z.; Li T.; Mudshinge S. R.; Xu B.; Hammond G. B. Optimization of catalysts and conditions in gold (ii) catalysis - counterion and additive e ff ects. Chem. Rev. 2021, 121, 8452–8477. 10.1021/acs.chemrev.0c00713. 33476128

[ref28] Davison N.; Mcmullin C. L.; Zhang L.; Hu S.; Waddell P. G.; Wills C.; Dixon C.; Lu E. Li vs na: divergent reaction patterns between organolithium and organosodium complexes and ligand-catalyzed ketone/aldehyde methylenation. J. Am. Chem. Soc. 2023, 145, 6562–6576. 10.1021/jacs.3c01033. 36890641 PMC10037334

[ref29] Navarro C. A.; Ma Y.; Michael K. H.; Breunig H. M.; Nutt S. R.; Williams T. J. Catalytic, aerobic depolymerization of epoxy thermoset composites. Green Chem. 2021, 23, 6356–6360. 10.1039/D1GC01970H.

[ref30] Brunelle D. J. Advances in Polycarbonates: An Overview; ACS Publication, 2005; Vol. 898.

[ref31] Wang C.; Singh A.; Rognerud E. R.; Murray R.; Musgrave G.; Skala M.; Murdy P.; DesVeaux J.; Nicholson S. R.; Harris K.; Canty R.; Mohr F.; Shapiro A. J.; Barnes D.; Beach R.; Allen R. D.; Beckham G. T.; Rorrer N. A. Synthesis, characterization, and recycling of bio-derivable polyester covalently adaptable networks for industrial composite applications. Matter10.1016/j.matt.2023.10.033.

[ref32] https://www.adityabirlachemicals.com/brand-list.php?id=recyclamine.

