# Supplementary material for: Base-Mediated Depolymerization of Amine-Cured Epoxy Resins
Source: ACS Sustain Chem Eng. 2023 Nov 20;11(48):16946–54. doi: 10.1021/acssuschemeng.3c04181 (PMC10698742; doi:10.1021/acssuschemeng.3c04181)
Supplement: Supplementary file 1 — sc3c04181_si_001.pdf [file sc3c04181_si_001.pdf]

Supporting Information for:

Base-mediated depolymerization of amine-cured epoxy resins

Rebecca C. DiPucchio, Katherine R. Stevenson, Ciaran W. Lahive, William E. Michener, Gregg T. Beckham

Renewable Resources and Enabling Sciences Center, National Renewable Energy Laboratory, Golden CO, 80401 USA

Number of pages: 59
Number of tables: 19
Number of figures: 65

Table of Contents

S1. Materials and Instrumentation 2
S2. Model Compound Syntheses and Characterization 2
S3. A GC-FID Method for Small Molecule Product Quantification from Model Compounds and Model Thermoplastics 7
S4. Oxidative Reaction Strategies Attempted 8
S5. Base-Mediated Depolymerization Reactions of Model Compounds in Microwave Vials 9
S5. Isolating and Characterizing Base Adduct Products from Model Compound Deconstruction Reactions 15
S6. Thermoplastic Syntheses and Characterization 21
S7. Thermoplastic Solubility Studies 45
S8. Base-Mediated Depolymerization Reactions of Model Thermoplastics in Microwave Vials 48
S9. Thermoset Syntheses and Characterization 56
S10. Thermoset Swelling Studies 59
S11. Thermoset Deconstruction Reactions 59

S1. Materials and Instrumentation

Potassium tert-butoxide (Sigma-Aldrich), 1,2-epoxy-3-phenoxypropane (Sigma-Aldrich), *N*-ethylaniline (Sigma-Aldrich), *N*-ethylcyclohexylamine (Sigma-Aldrich), bisphenol a diglycidyl ether (Sigma-Aldrich), aniline (Sigma-Aldrich), cyclohexylamine (TCI Chemicals), isophorone diamine (TCI Chemical), and 4,4'-diaminodiphenylsulfone (EMD Millipore) were used as received.

All NMR spectra were recorded on a Bruker 300 MHz spectrometer at ambient temperature. Gas chromatograms of starting materials and resultant products were analyzed on an Agilent 7890A GC-FID System, unless otherwise noted. Polymer characterizations were conducted using either TA Discovery Series TGA 5500, TA Discovery Series DSC 25, and Perkin Elmer FT-IR Spectrometers. 20 mL microwave vials and caps used were purchased from Biotage. Each DSC cycle consistent of 5 min holds at 0 °C, followed by a 1.5 °C/min ramp to 200 °C, followed by a 5 min hold and a ramp down to 0 °C at the same rate. DSC data reported represent the second cycle for each run, except for curing data where both cycles are reported. Our TGA method consists of a ramp from 0 °C to 700 °C at 10 °C/min, followed by a 5 min hold at 700 °C.

All GPC data were collected on an Agilent 1260 Infinity II GPC with Wyatt detectors. Columns consisted of three Agilent PLgel MIXED-C, 7.5 x 300 mm, 5 µm, HPLC columns attached in series, with a matching guard column attached. HPLC grade THF solvent purchased from Sigma Aldrich was used as the mobile phase. The THF solvent was not filtered. Samples were dissolved in the THF solvent at a concentration of 20mg/mL, with a 30 kDa polystyrene standard prepared at 5 mg/mL. The samples were then filtered through a 2um filter directly into a 1.5mL GC vial. The operating conditions included using THF as the mobile phase, a flow rate of 1.0 mL/min, column oven temperature set to 40 °C, and a sample injection of 100uL. Detectors consisted of a miniDawn Multi-Angle Light Scattering detector (Wyatt Technology) used in combination with a Optilab Refractive Index detector (Wyatt Technology) and a Viscostar viscometer (Wyatt Technolgies). Wyatt Technologies Astra Software was used to analyze data.

Note that GPC and NMR data for partially crosslinked thermoplastics (**7**, **10**, **11**) reflects only the soluble portions of those thermoplastics, while thermal data reflects the entire polymer system.

S2. Model Compound Syntheses and Characterization

General procedure for the synthesis of small molecule model compounds: Compounds were prepared by combining a chosen amine and with an equimolar amount of 1,2-epoxy-3-phenoxypropane. The resultant solution was stirred as a neat mixture at 700 rpm for forty eight hours at 110 °C. The resultant oil products were used and characterized without any further purification.

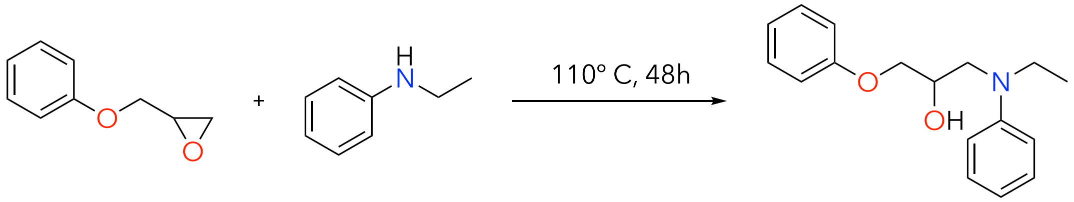

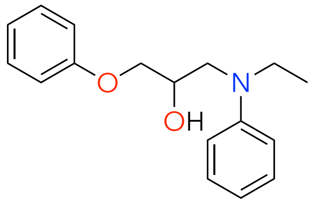

Synthesis of 1-(ethyl(phenyl)amino)-3-phenoxypropan-2-ol (2): Prepared following the general procedure outlined above: *N*-ethylaniline (20.0 g, 165 mmol), 1,2-epoxy-3-phenoxypropane (24.8 g, 165 mmol). ^{1}H NMR (300 MHz, $CDCl_3$, 298 K): δ 7.25 (m, 4H, m-C_6H_5, m-C_6H_5), 6.95 (m, 3H, p-C_6H_5, o-C_6H_5), 6.79 (d, 2H, o-C_6H_5), 6.72 (t, 1H, p-C_6H_5), 4.26 (s, 1H, OH), 4.02 (m, 2H, NCH_2), 3.44 (m, 4H, OCH_2, NCH_2), 2.54 (s, 1H, CH), 1.14 (t, 3H, CH_3) ppm. ^{13}C NMR (75 MHz, $CDCl_3$, 298 K): δ 158.48, 148.44, 129.54, 129.34, 121.23, 116.99, 114.59, 113.20, 69.61, 68.07, 53.37, 46.13, 11.57 ppm.

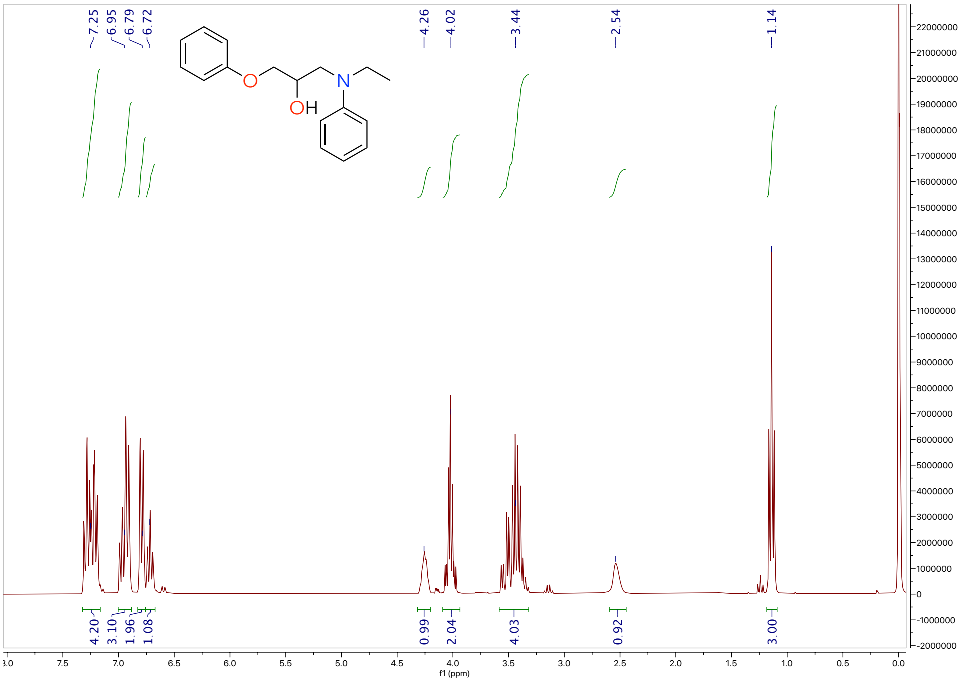

Figure S1. ^{1}H NMR spectrum (300 MHz, $CDCl_3$, 298 K) of 1-(ethyl(phenyl)amino)-3-phenoxypropan-2-ol. Residual $CDCl_3$ peak not visible due to concentration of sample.

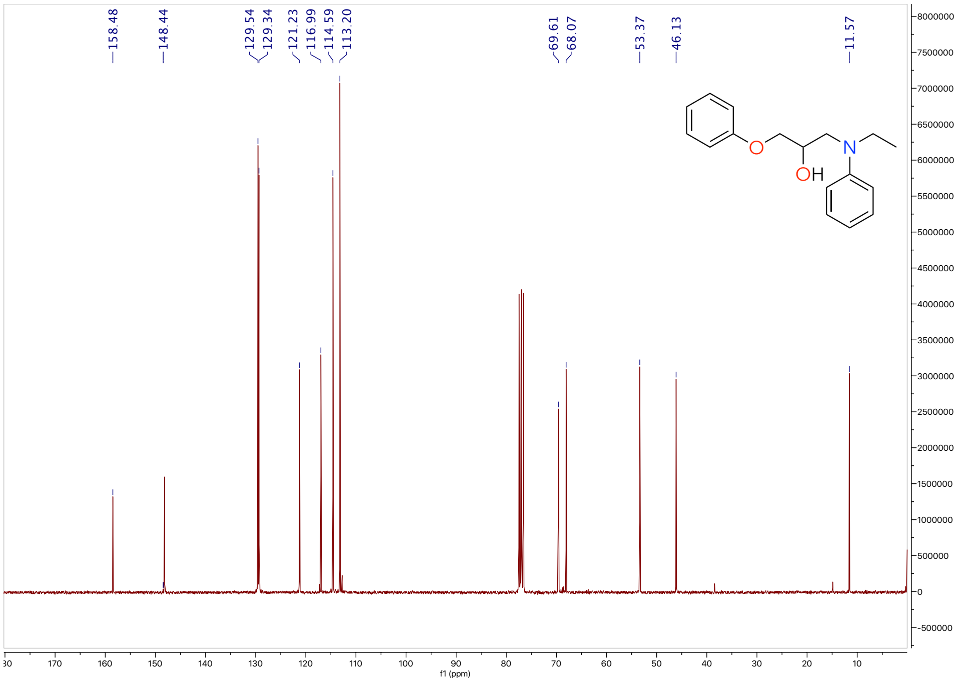

Figure S2. ^{13}C NMR spectrum (75 MHz, $CDCl_3$, 298 K) of 1-(ethyl(phenyl)amino)-3-phenoxypropan-2-ol.

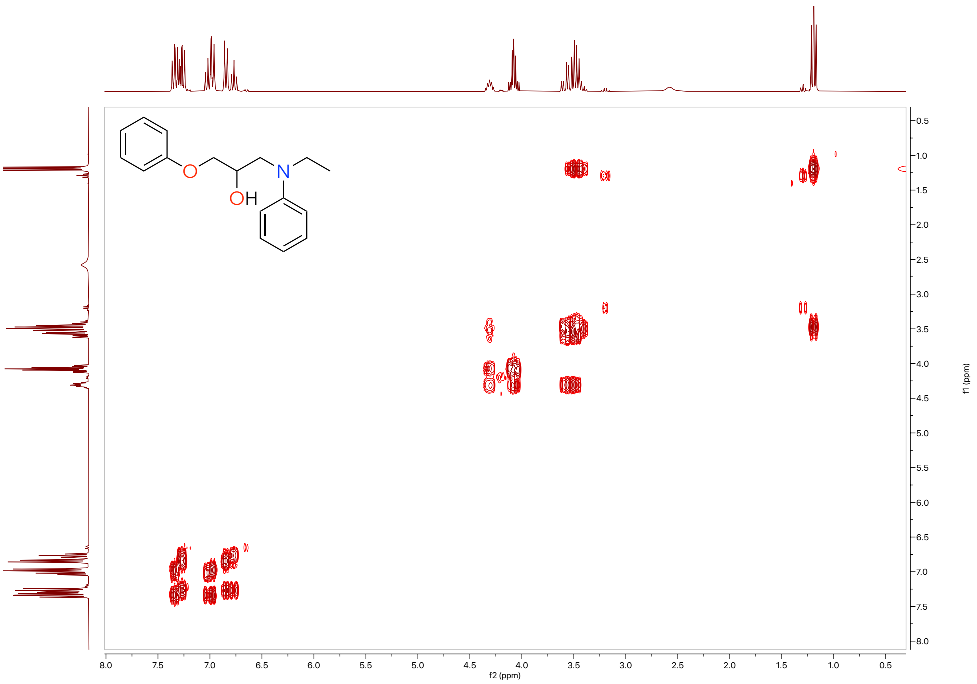

Figure S3. COSY NMR spectrum (300 MHz, $CDCl_3$, 298 K) of 1-(ethyl(phenyl)amino)-3-phenoxypropan-2-ol.

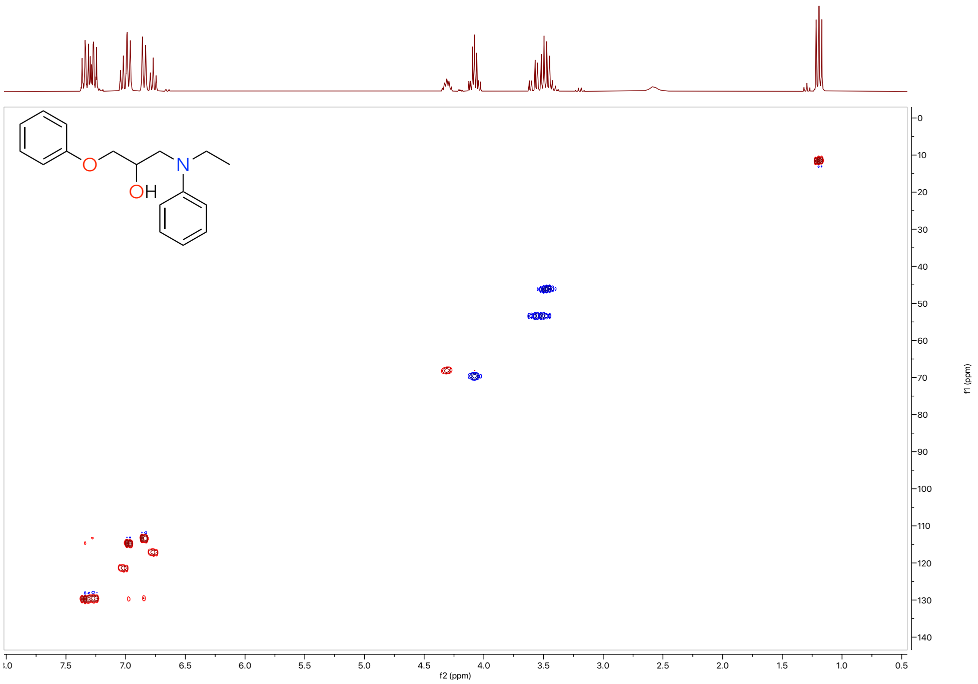

Figure S4. HSQC NMR spectrum (300 MHz/ 75 MHz, $CDCl_3$, 298 K) of 1-(ethyl(phenyl)amino)-3-phenoxypropan-2-ol.

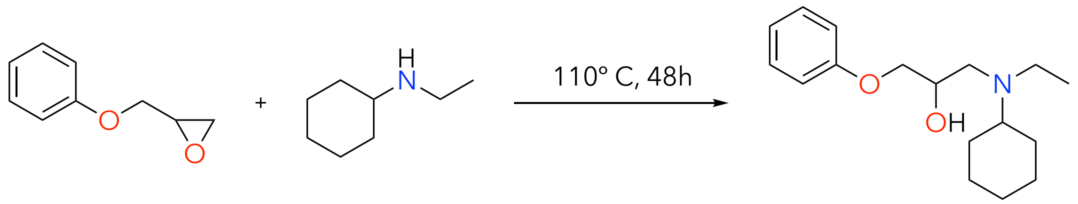

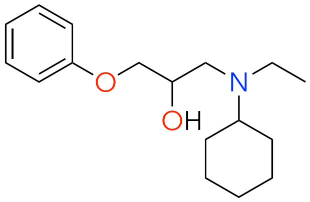

Synthesis of 1-(cyclohexyl(ethyl)amino)-3-phenoxypropan-2-ol (3): Prepared following the general procedure outlined above: *N*-ethylcyclohexylamine (2.5035 g, 19.65 mmol), 1,2-epoxy-3-phenoxypropane (2.947 g, 19.65 mmol). ^{1}H NMR (300 MHz, $CDCl_3$, 298 K): δ 7.28 (dd, 2H, m-C_6H_5), 6.94 (t, 3H, o,p-C_6H_5), 3.95 (m, 3H, OCH_2CH), 2.58 (m, 5H, $CH_2N(CH)CH_2$), 1.82 (t, 3H, C_6H_{11}), 1.68 (t, 2H, C_6H_{11}), 1.27 (m, 5H, C_6H_{11}), 1.06 (t, 3H, CH_3) ppm. ^{13}C NMR (75 MHz, $CDCl_3$, 298 K): δ 158.91, 129.55, 121.25, 114.55, 70.58, 65.81, 59.93, 52.63, 44.63, 30.96, 27.74, 26.27, 26.07, 14.73 ppm.

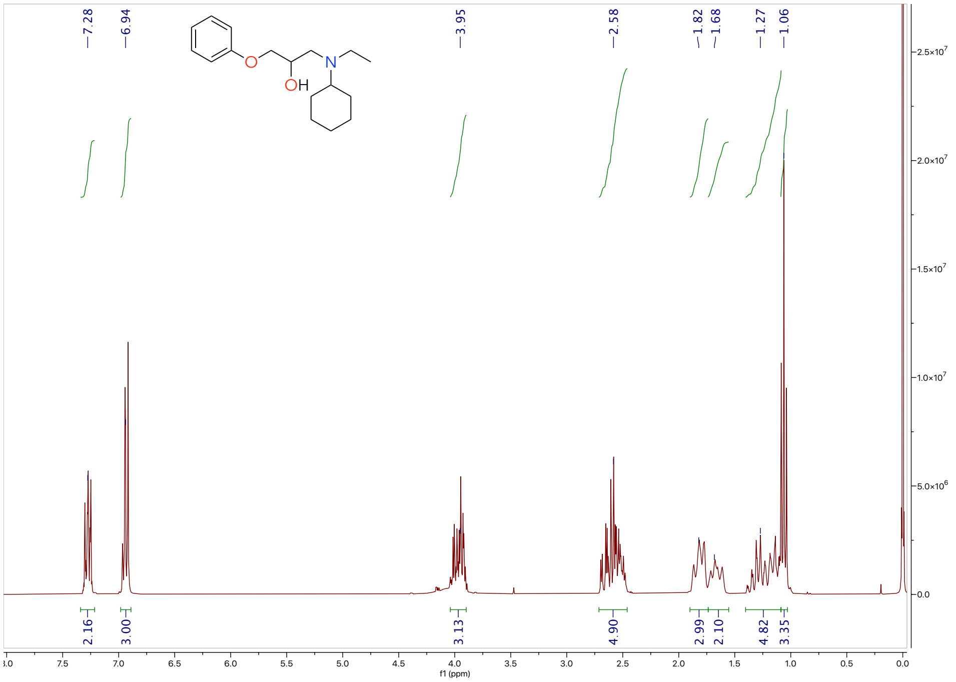

Figure S5. ^{1}H NMR spectrum (300 MHz, $CDCl_3$, 298 K) of 1-(cyclohexyl(ethyl)amino)-3-phenoxypropan-2-ol. Residual $CDCl_3$ peak not visible due to concentration of sample.

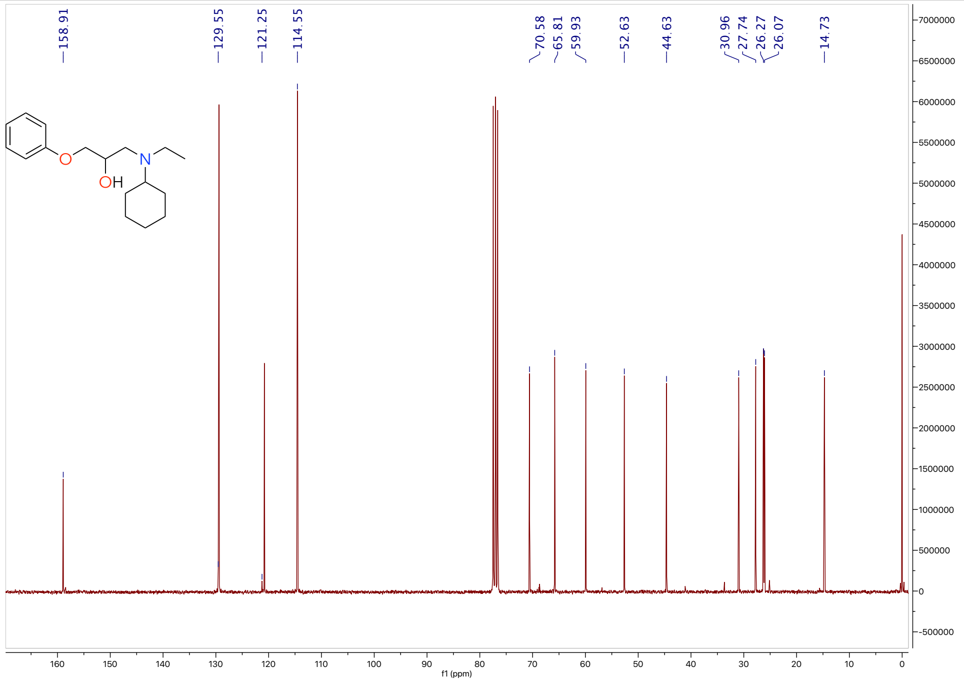

Figure S6. ^{13}C NMR spectrum (75 MHz, $CDCl_3$, 298 K) of 1-(cyclohexyl(ethyl)amino)-3-phenoxypropan-2-ol.

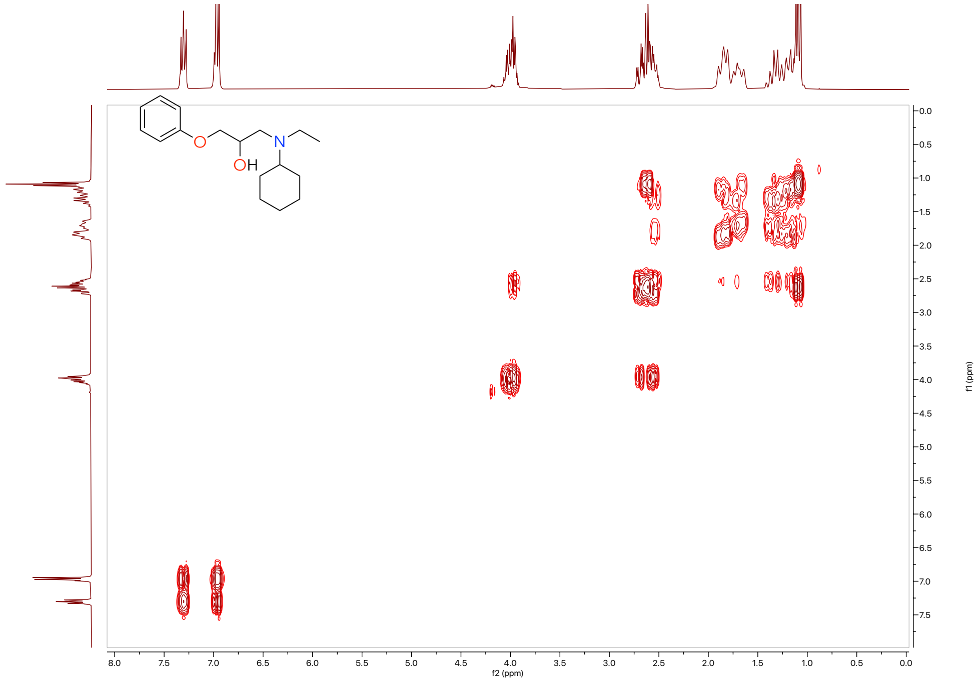

Figure S7. COSY NMR spectrum (300 MHz, $CDCl_3$, 298 K) of 1-(cyclohexyl(ethyl)amino)-3-phenoxypropan-2-ol.

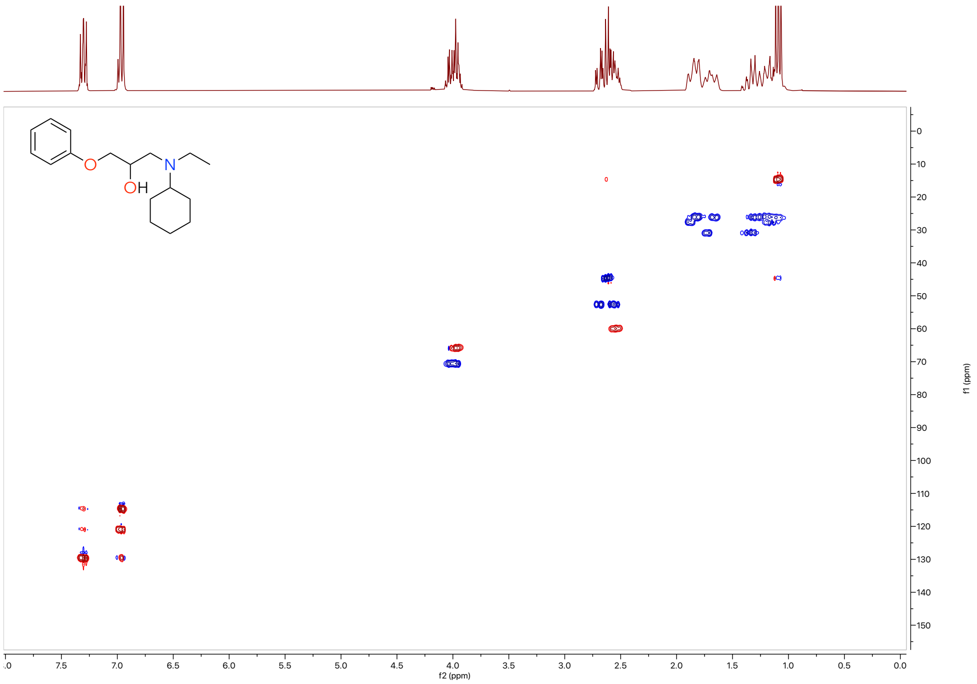

Figure S8. HSQC NMR spectrum (300 MHz/ 75 MHz, $CDCl_3$, 298 K) of 1-(cyclohexyl(ethyl)amino)-3-phenoxypropan-2-ol.

S3. A GC-FID Method for Small Molecule Product Quantification from Model Compounds and Model Thermoplastics

The GC method used in this paper was developed on an Agilent 7890A GC instrument eqipeed with a flame ionization detector (FID), a CP-Sil-8 CB column (part # CP8751), and a split/splitless (S/SL) inlet. The method begins at 30 °C, with a 5-minute hold. This is followed by a 25 °C/min ramp to 300 °C and a second 5-minute hold. The injection volume for each sample is 0.2 uL, with a 0.5 uL syringe and an acetone rinse. 9-point calibration curves were made for all compounds below, with linear ranges of 10–1000ppm and R^2 values of >.995. The only exception to this is compounds **4B** and **5**, which could not be isolated in sufficient amounts for calibration curves. Compound **4B** was quantified using the response factor for compound **4A**, while compound **5** was quantified using the response factor for **4**. Note that calibration verification standards (CVSs) for **4** and **4A** revealed some minor increases over many injections, though standard concentrations were as expected at the beginning of every run. HPLC grade acetone was used as a dilution and rinse solvent for all samples in this paper. All reaction samples were prepared using a 100x dilution from neat reaction samples in HPLC grade acetone. Standards for each sample are shown below, followed by a sample reaction sample for deconstruction reactions with **2**.

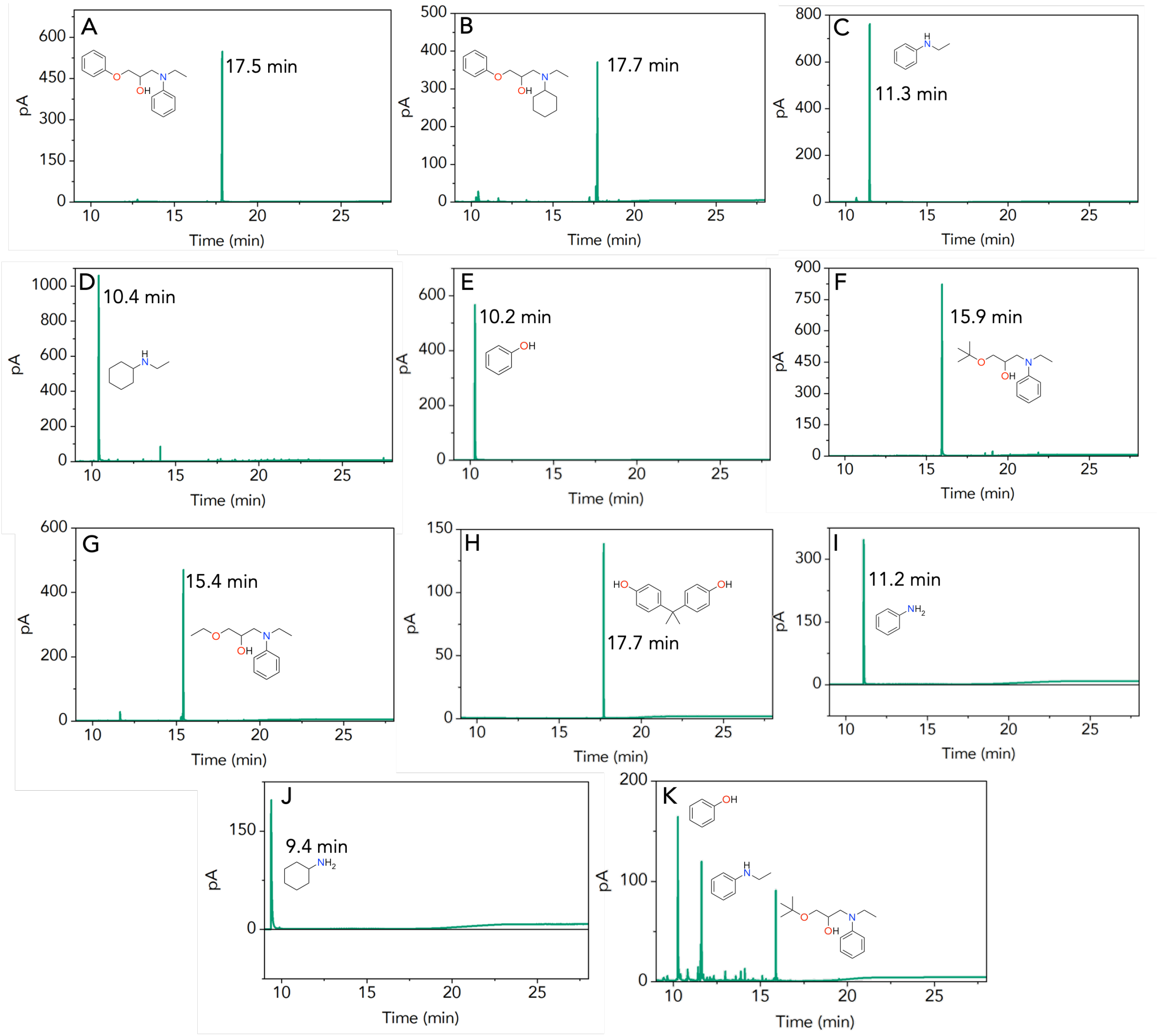

Figure S9. A–**J** in this figure are standard GC-FID data at 500 ppm for compounds as quantified in this work. (**A**) GC data for 1-(ethyl(phenyl)amino)-3-phenoxypropan-2-ol. (**B**) GC data for 1-(cyclohexyl(ethyl)amino)-3-phenoxypropan-2-ol. (**C**) GC data for N-ethylaniline. (**D**) GC for N-ethylcyclohexylamine. (**E**) GC data for phenol. (**F**) GC data for 1-(tert-butoxy)-3-(ethyl(phenyl)amino)propan-2-ol. (**G**) GC data for 1-ethoxy-3-(ethyl(phenyl)amino)propan-2-ol. (**H**) GC data for bisphenol A. (**I**) GC data for aniline. (**J**) GC data for cyclohexylamine. (**K**) A GC-FID chromatogram from a sample deconstruction reaction with **2**. Reaction conditions for this reaction were 4 eq. KOtBu, 3 mL 1:1 Toluene:THF, 140 °C, 24h.

S4. Oxidative Reaction Strategies Attempted

Initial reaction tests focused on oxidants and Lewis acids as reactions to cleave C–O bonds with air as an oxidant. These results are summarized briefly in **Table S1** below. All reactions in this section were run in 75 mL Parr reactors. As noted in the main text, amine content is very low in all cases as compared with base reactions below. Notably, amine products from both model compounds and polymers decomposed during stability tests under similar oxidative/ acidic reaction conditions.

Table S1. A summary of oxidative reaction conditions attempted for C-O bond cleavage in an epoxy model compound (**2**). In this table, NHPI represents *N*-Hydroxyphthalimide. Yield data are percentages, were calculated via GC-FID analyses, and are an average of two runs. All reactions were run at 90 °C for 16 hours.

Entry	Substrate	Conditions	Starting Material	Phenol	Total Amine Yield
1	**2**	4 mol% $Co(OAc)_2$, 10 mol% NHPI, 60 bar He	0	38	14
2	**2**	4 mol% $Co(OAc)_2$, 10 mol% NHPI, 31 bar He/29 bar air	0	61	22
3	**2**	4 mol% $Mn(OAc)_2$, 10 mol% NHPI, 60 bar He	0	37	15
4	**2**	4 mol% $Mn(OAc)_2$, 10 mol% NHPI, 31 bar He/29 bar air	0	49	19
5	**2**	4 mol% $Zr(acac)_2$, 10 mol% NHPI, 60 bar He	0	24	14
6	**2**	4 mol% $Zr(acac)_2$, 10 mol% NHPI, 31 bar He/29 bar air	0	60	19

S5. Base-Mediated Depolymerization Reactions of Model Compounds in Microwave Vials

General procedure (GP1) for model compound deconstruction reactions in microwave vials:
0.5 mmol of a representative model compound and desired equivalents of base were weighed and directly added to a 20 mL Biotage microwave vial with a magnetic stir bar. Appropriate volumes of solvent(s) (THF and/or toluene) were added via a positive displacement pipette before sealing the reaction with a Biotage cap septum via an automatic crimper set to 55% tightness. The vial was then placed in a preheated pie plate heat block on a hot plate and reacted while stirring at 650 rpm. The vial was removed from heat after varying times. Reactions were then directly sampled by measuring a 10uL sample with a 10uL positive displacement pipette and diluting with 990uL of HPLC grade acetone. This creates a 100x reaction dilution for direct GC-FID quantification of products and any remaining starting material.

Reaction optimization studies:

Table S2. Comparing deconstruction reactivity with groups 1 and 2 hydroxide bases. Number values represent % yield values measured via GC-FID.

Entry	Substrate	Conditions	2	Phenol	Free Amine	4
1	**2**	4 eq. LiOH, 3 mL 1:1 Toluene:THF, 140 °C, 24h	67	19	27	0.00
2	**2**	4 eq. NaOH, 3 mL 1:1 Toluene:THF, 140 °C, 24h	70	24	32	0.00
3	**2**	4 eq. KOH, 3 mL 1:1 Toluene:THF, 140 °C, 24h	56	20	30	0.00
4	**2**	4 eq. $Ca(OH)_2$, 3 mL 1:1 Toluene:THF, 140 °C, 24h	77	10	23	0.00
5	**2**	4 eq. $Sr(OH)_2$, 3 mL 1:1 Toluene:THF, 140 °C, 24h	82	21	28	0.00

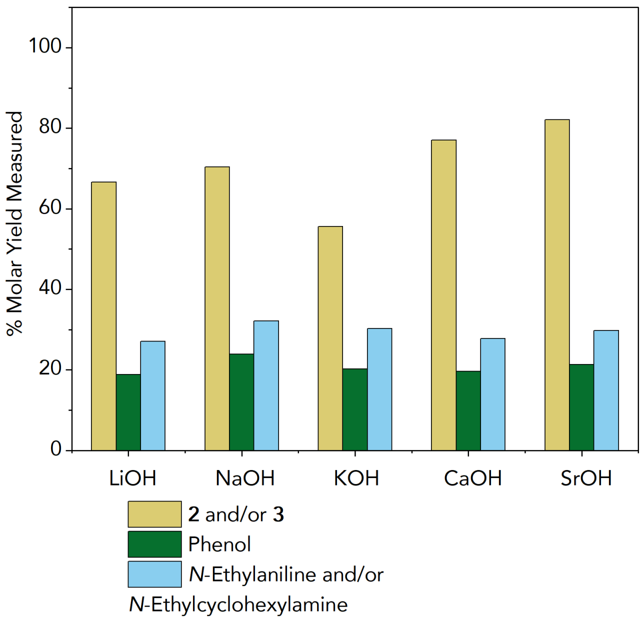

Figure S10. Comparing deconstruction reactivity with groups 1 and 2 hydroxide bases.

Table S3. Comparing deconstruction reactivity with KO*t*Bu, KOEt, and KOMe. Number values represent % yield values measured via GC-FID.

Entry	Substrate	Conditions	Starting Material	Phenol	Free Amine	Amine Adduct
1	**2**	4 eq. KOMe, 1:1 Toluene:THF, 140C, 24h	0	71	19	25
2	**2**	4 eq. KOEt, 1:1 Toluene:THF, 140C, 24h	0	96	33	30
3	**2**	4 eq. KOtBu, 1:1 Toluene:THF, 140C, 24h	5	99	25	66

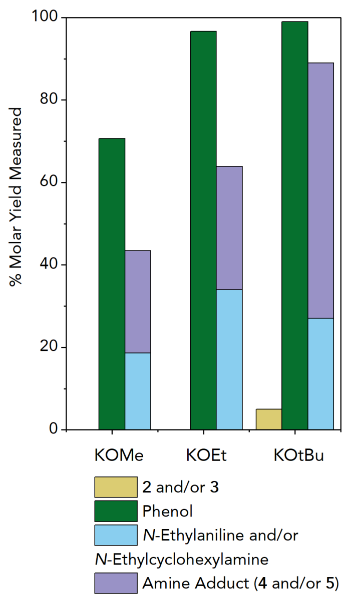

Figure S11. Comparing deconstruction reactivity with KOtBu, KOEt, and KOMe.

Table S4. Comparing sodium, lithium, and potassium tert-butoxide bases. Number values represent % yield values measured via GC-FID.

Entry	Substrate	Conditions	2	Phenol	Free Amine	4
1	**2**	4 eq. LiOtBu, 1:1 Toluene:THF, 140 °C, 24h	86	14	14	0.00
2	**2**	4 eq. KOtBu, 1:1 Toluene:THF, 140 °C, 24h	5	99	25	66
3	**2**	4 eq. NaOtBu, 1:1 Toluene:THF, 140 °C, 24h	75	34	20	9

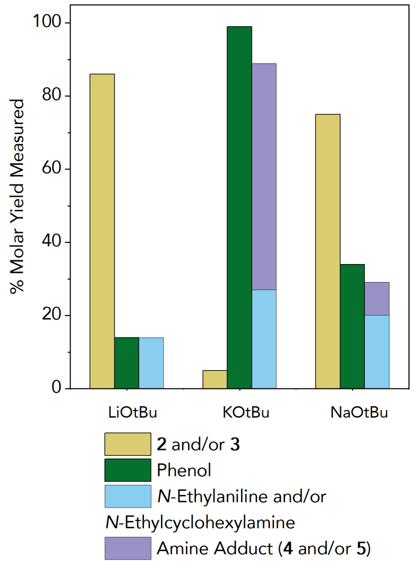

Figure S12. Comparing sodium, lithium, and potassium tert-butoxide bases.

Table S5. Comparing deconstruction reactivity with different equivalents of KOtBu at 140 °C. Number values represent % yield values measured via GC-FID.

Entry	Substrate	Conditions	2	Phenol	Free Amine	4
1	**2**	1 eq. KOtBu, 3 mL 1:1 Toluene:THF, 140 ℃, 24h	94	16	16	0
2	**2**	2 eq. KOtBu, 3 mL 1:1 Toluene:THF, 140 ℃, 24h	9	94	28	17
3	**2**	3 eq. KOtBu, 3 mL 1:1 Toluene:THF, 140 ℃, 24h	7	100	27	49
4	**2**	4 eq. KOtBu, 3 mL 1:1 Toluene:THF, 140 ℃, 24h	5	99	27	62

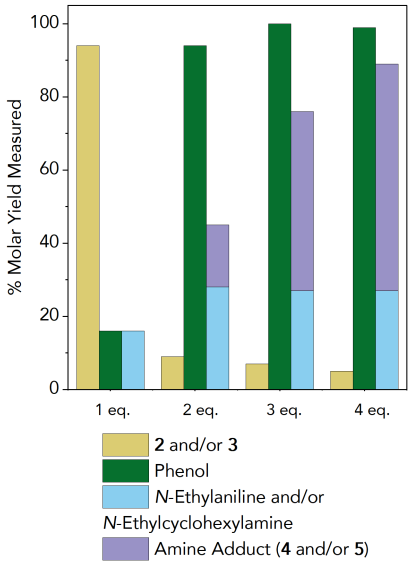

Figure S13. Comparing deconstruction reactivity with different equivalents of KOtBu at 140 °C.

Table S6. Comparing deconstruction reactivity with different equivalents of KOtBu at 160 °C. Number values represent % yield values measured via GC-FID.

Entry	Substrate	Conditions	2	Phenol	Free Amine	4
1	**2**	1 eq. KOtBu, 3 mL 1:1 Toluene:THF, 160 ℃, 24h	37	46	20	0
2	**2**	2 eq. KOtBu, 3 mL 1:1 Toluene:THF, 160 ℃, 24h	8	92	16	33
3	**2**	3 eq. KOtBu, 3 mL 1:1 Toluene:THF, 160 ℃, 24h	7	100	33	47
4	**2**	4 eq. KOtBu, 3 mL 1:1 Toluene:THF, 160 ℃, 24h	0	102	35	66

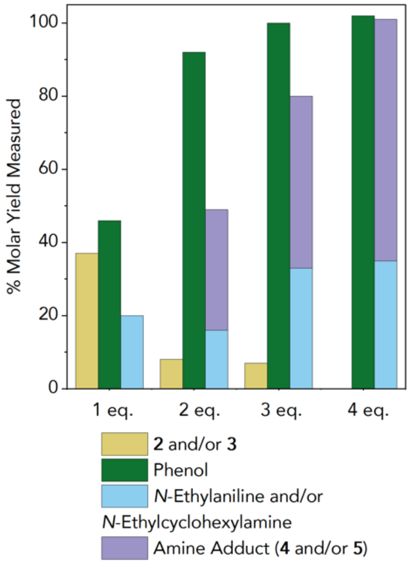

Figure S14. Comparing deconstruction reactivity with different equivalents of KOtBu at 160 °C.

Table S7. Comparing deconstruction reactivity with different reaction volumes. Number values represent % yield values measured via GC-FID.

Entry	Substrate	Conditions	2	Phenol	Free Amine	4
1	**2**	4 eq. KotBu, 1 mL 1:1 Toluene:THF, 140 °C, 24h	0	104	27	65
2	**2**	4 eq. KotBu, 2 mL 1:1 Toluene:THF, 140 °C, 24h	0	106	26	76
3	**2**	4 eq. KotBu, 3 mL 1:1 Toluene:THF, 140 °C, 24h	0	99	25	69
4	**2**	4 eq. KotBu, 4 mL 1:1 Toluene:THF, 140 °C, 24h	0	101	29	65
5	**2**	4 eq. KotBu, 5 mL 1:1 Toluene:THF, 140 °C, 24h	0	98	28	64
6	**2**	4 eq. KotBu, 6 mL 1:1 Toluene:THF, 140 °C, 24h	0	98	29	63
7	**2**	4 eq. KotBu, 7 mL 1:1 Toluene:THF, 140 °C, 24h	0	100	30	61
8	**2**	4 eq. KotBu, 8 mL 1:1 Toluene:THF, 140 °C, 24h	0	101	28	66
9	**2**	4 eq. KotBu, 9 mL 1:1 Toluene:THF, 140 °C, 24h	0	94	28	61

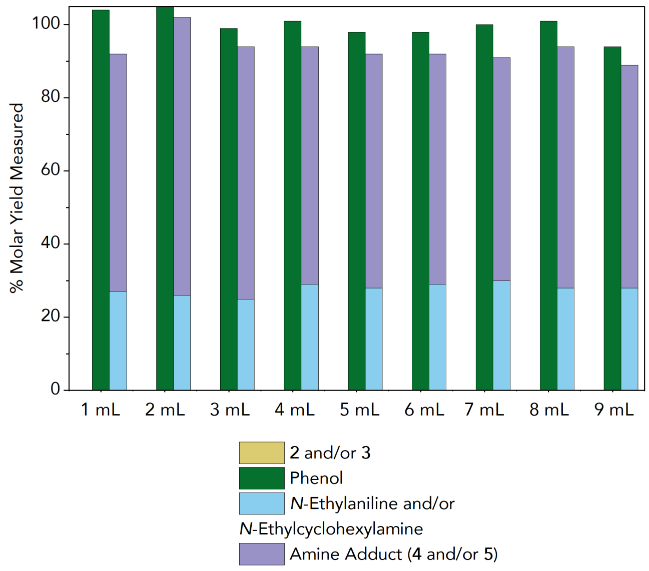

Figure S15. Comparing deconstruction reactivity with different reaction volumes.

Table S8. Yield data that was used for **figure 3** in the main body of this paper. Number values represent % yield values measured via GC-FID.

Entry	Substrate	Conditions	Starting Material	Phenol	Free Amine	Amine Adduct
1	**2**	4 eq. KotBu, 3 mL 1:1 Toluene:THF, 140 °C, 24h	8	98	30	52
2	**2**	4 eq. KotBu, 3 mL 1:1 Toluene:THF, 140 °C, 24h	7	99	26	66
3	**2**	4 eq. KotBu, 3 mL 1:1 Toluene:THF, 140 °C, 24h	0	101	25	69
		Average =	5	99	27	62
		SD =	4	1	2	7
4	**3**	4 eq. KotBu, 3 mL 1:1 Toluene:THF, 140 °C, 24h	0	86	32	49
5	**3**	4 eq. KotBu, 3 mL 1:1 Toluene:THF, 140 °C, 24h	0	92	33	57
6	**3**	4 eq. KotBu, 3 mL 1:1 Toluene:THF, 140 °C, 24h				
		Average =	0	89	32	53
		SD =	0	3	0.3	4
7	**2 + 3**	4 eq. KotBu, 3 mL 1:1 Toluene:THF, 140 °C, 24h	0	101	35	63
8	**2 + 3**	4 eq. KotBu, 3 mL 1:1 Toluene:THF, 140 °C, 24h	0	93	35	65
9	**2 + 3**	5 eq. KotBu, 3 mL 1:1 Toluene:THF, 140 °C, 24h	0	85	39	47
		Average =	0	93	36	58
		SD =	0	7	2	8

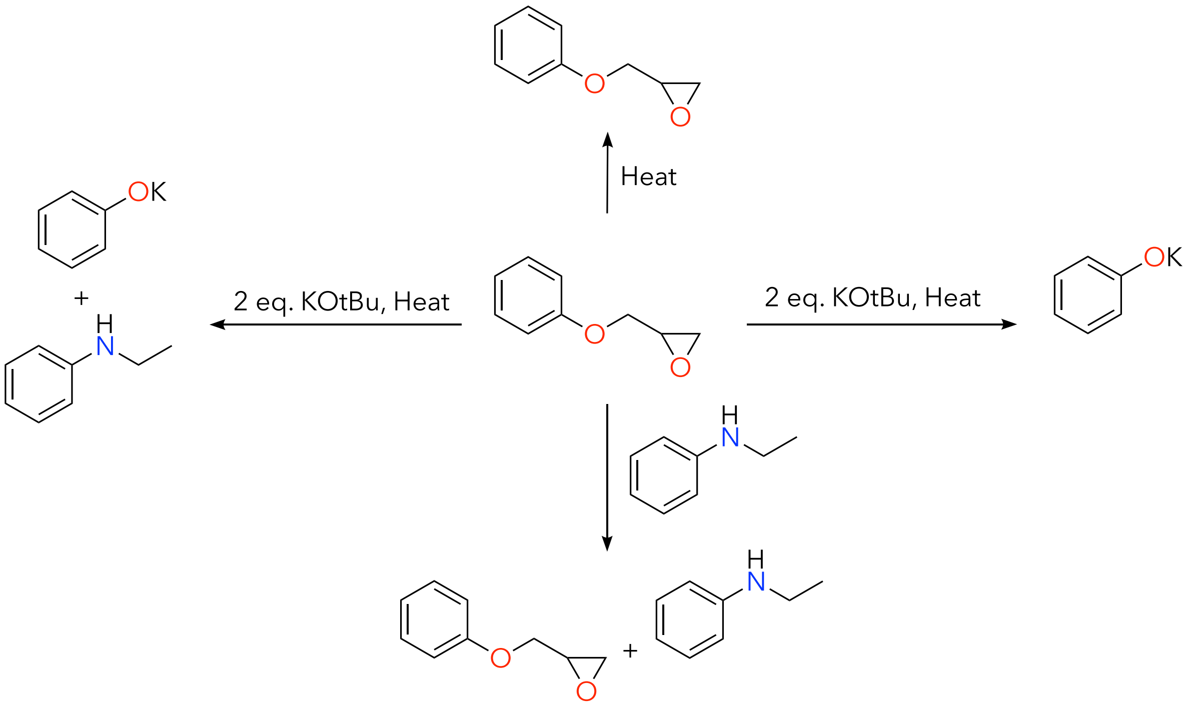

Figure S16. Qualitative results from testing reactivity with **1** as a proposed reaction intermediate. In this figure, heat refers to 24 h at 140 °C in 1:1 THF:toluene.

S5. Isolating and Characterizing Base Adduct Products from Model Compound Deconstruction Reactions

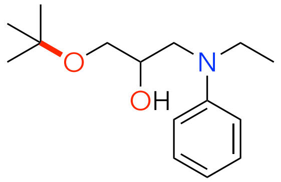

Synthesis of 1-(tert-butoxy)-3-(ethyl(phenyl)amino)propan-2-ol (4): Prepared following the general procedure outlined above (**GP1**). The crude reaction mixture was then directly dried under vacuum. The resultant oil was redissolved in a minimal amount of dichloromethane and purified via normal phase automated column chromatography on a Teledyne ISCO instrument. Compound **4** was isolated in a 21 % isolated yield (24 mg). ^{1}H NMR (300 MHz, $CDCl_3$, 298 K): δ 7.21 (m, 2H, m-C_6H_5), 6.78 (d, 2H, o-C_6H_5), 6.69 (t, 1H, p-C_6H_5), 3.97 (m, 1H, OH), 3.40 (m, 6H, CH_2), 2.52 (d, 1H, CH), 1.22-1.17 (s, 9H, CH_3), 1.15-1.13 (t, 3H, CH_3) ppm. ^{13}C NMR (75 MHz, $CDCl_3$, 298 K): δ 148.29, 129.23, 116.31, 112.71, 73.27, 68.73, 63.37, 53.43, 45.67, 27.58, 11.56 ppm.

Note: 2D NMR spectra for this compound serves as a representative example for the following two base adducts.

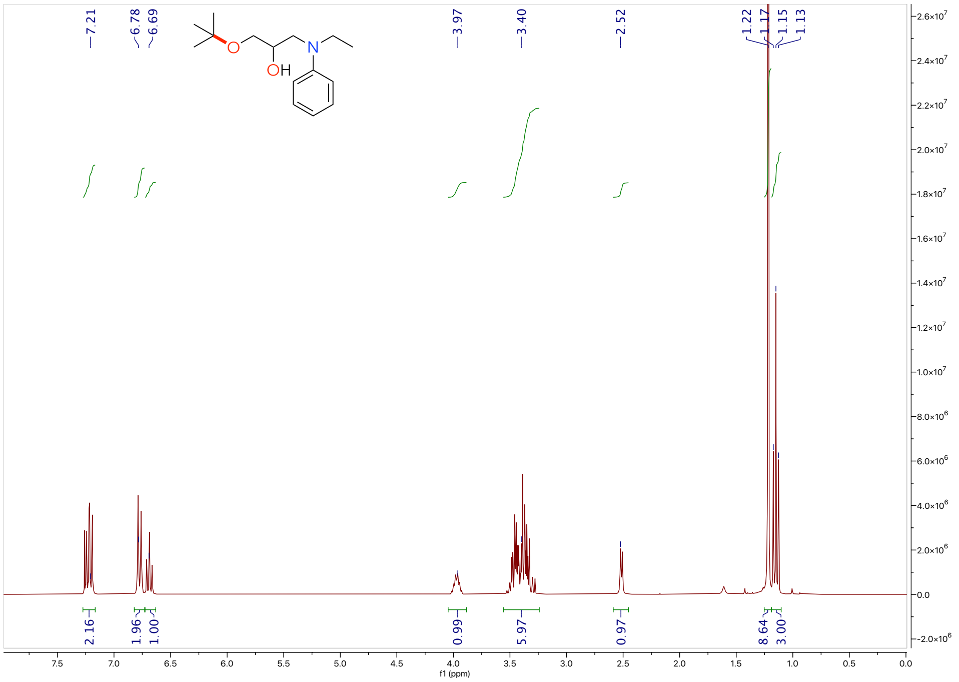

Figure S17. ^{1}H NMR spectrum (300 MHz, $CDCl_3$, 298 K) of 1-(tert-butoxy)-3-(ethyl(phenyl)amino)propan-2-ol.

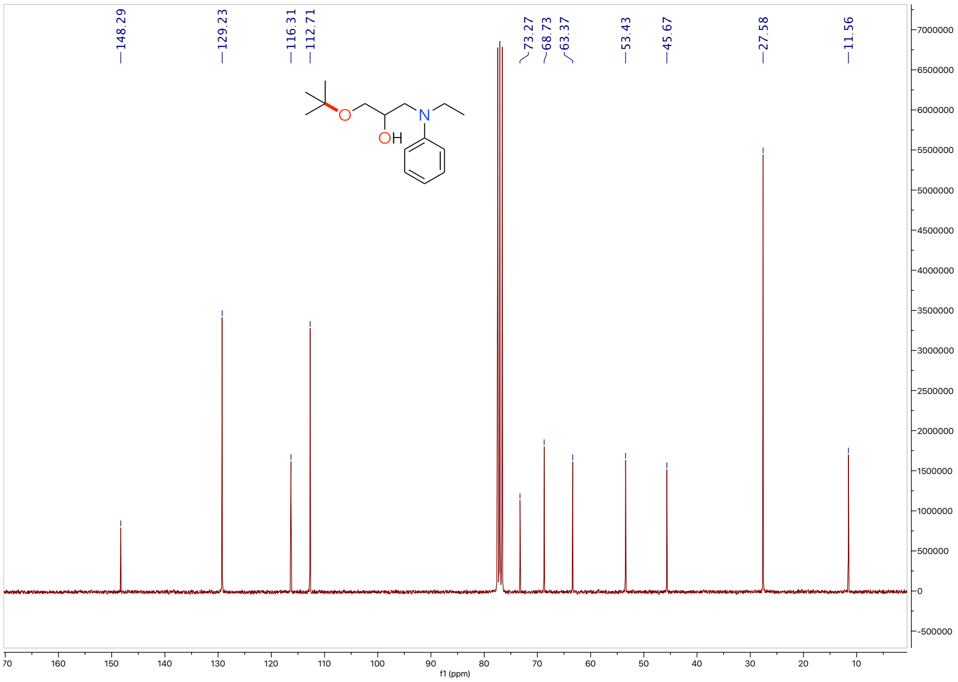

Figure S18. ^{13}C NMR spectrum (75 MHz, $CDCl_3$, 298 K) of 1-(tert-butoxy)-3-(ethyl(phenyl)amino)propan-2-ol.

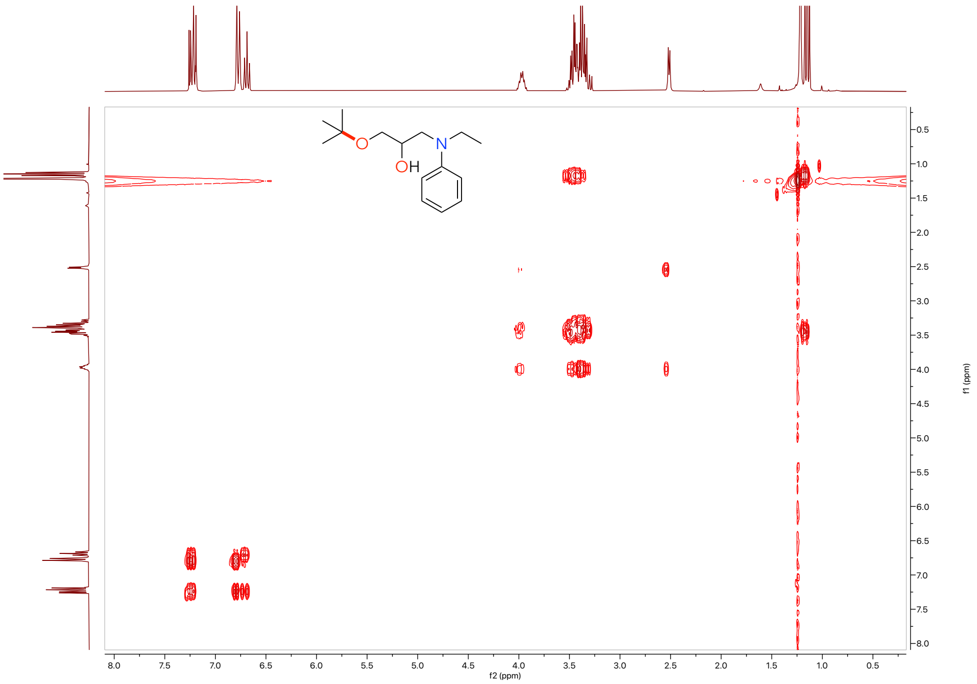

Figure S19. COSY NMR spectrum (300 MHz, $CDCl_3$, 298 K) of 1-(tert-butoxy)-3-(ethyl(phenyl)amino)propan-2-ol.

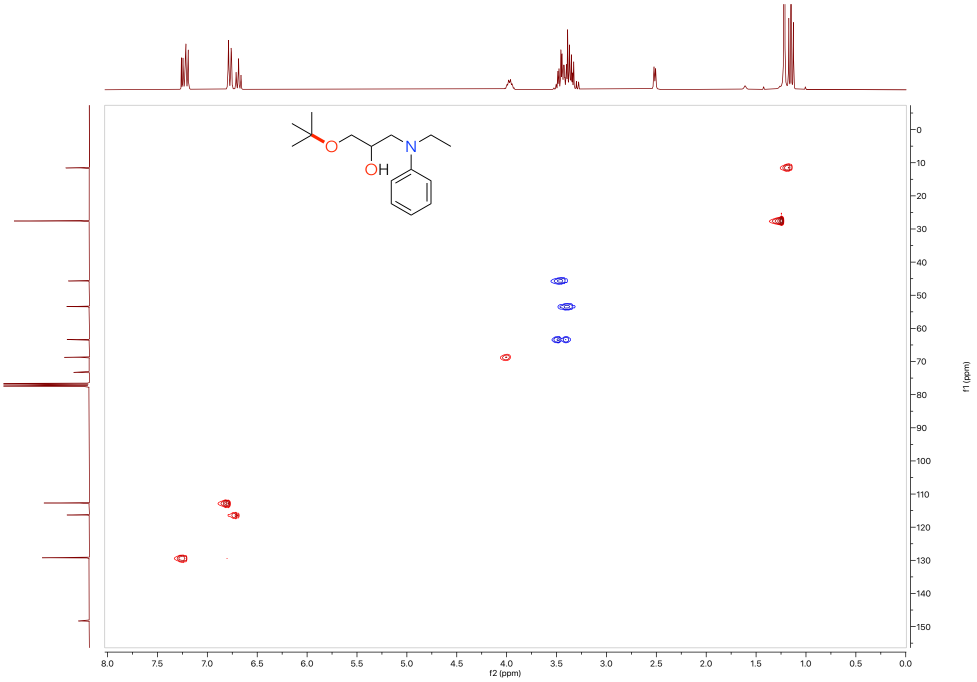

Figure S20. HSQC NMR spectrum (300 MHz/ 75 MHz, $CDCl_3$, 298 K) of 11-(tert-butoxy)-3-(ethyl(phenyl)amino)propan-2-ol.

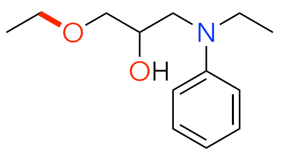

Synthesis of 1-ethoxy-3-(ethyl(phenyl)amino)propan-2-ol (4a): Prepared following the general procedure outlined above (**GP1**). The crude reaction mixture was then directly dried under vacuum. The resultant oil was redissolved in a minimal amount of dichloromethane and purified via normal phase automated column chromatography on a Teledyne ISCO instrument. Compound **4a** was isolated in a 25 % isolated yield (29 mg). ^{1}H NMR (300 MHz, $CDCl_3$, 298 K): δ 7.25 (m, 2H, m-C_6H_5), 6.83 (m, 2H, o-C_6H_5), 6.73 (t, 1H, p-C_6H_5), 4.07 (s, 1H, OH), 3.54-3.44 (m, 8H, CH_2), 2.52-2.51 (d, 1H, CH), 1.29-1.24 (t, 3H, CH_3), 1.20-1.15 (t, 3H, CH_3) ppm. ^{13}C NMR (75 MHz, $CDCl_3$, 298 K): δ 148.26, 148.20, 129.27, 129.16, 116.57, 112.87, 72.20, 68.53, 66.87, 53.32, 45.79, 15.16, 11.54 ppm.

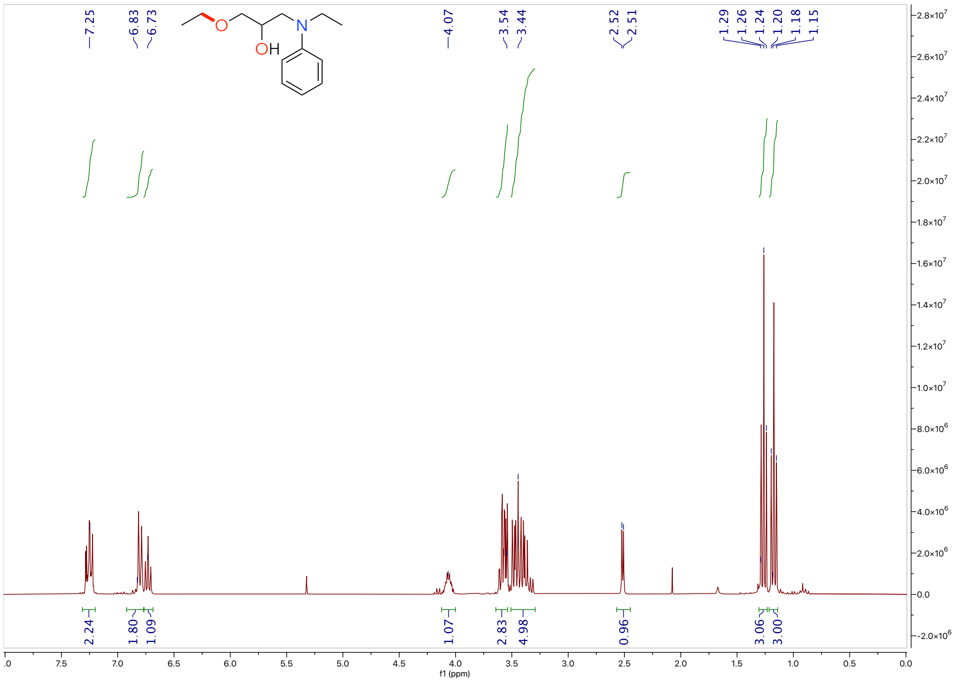

Figure S21. ^{1}H NMR spectrum (300 MHz, $CDCl_3$, 298 K) of 1-ethoxy-3-(ethyl(phenyl)amino)propan-2-ol.

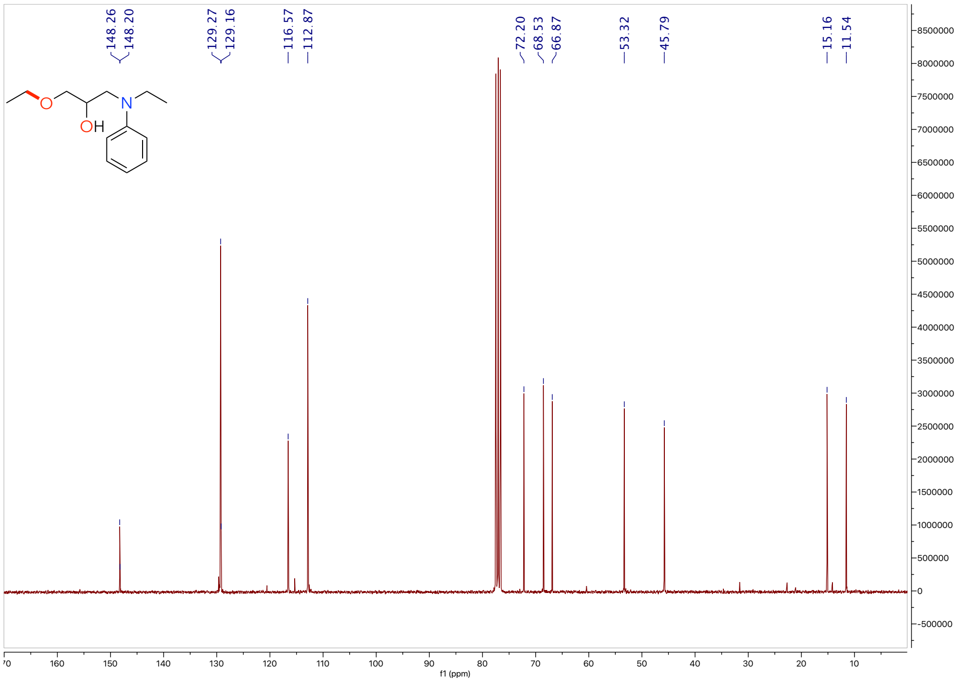

Figure S22. ^{13}C NMR spectrum (75 MHz, $CDCl_3$, 298 K) of 1-ethoxy-3-(ethyl(phenyl)amino)propan-2-ol.

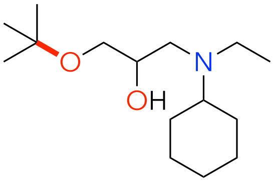

Synthesis of 1-(tert-butoxy)-3-(cyclohexyl(ethyl)amino)propan-2-ol (5): Prepared following the general procedure outlined above (**GP1**). The crude reaction mixture was then directly dried under vacuum. The resultant oil was redissolved in a minimal amount of dichloromethane and purified via normal phase automated column chromatography on a Teledyne ISCO instrument. Compound **5** was isolated in a 8 % isolated yield (10 mg). ^{1}H NMR (300 MHz, $CDCl_3$, 298 K): δ 3.67 (m, 1H, CH), 3.43-3.31 (m, 2H, CH_2), 2.25 (m, 5H, $CH_2N(CH)CH_2$), 1.82-1.68 (m, 6H, p,o-C_6H_{11}), 1.34-1.05 (m, 16H, m-C_6H_{11}, CH_3), 1.22 (s, 9H), 1.11 (m, 2H), 1.05 (t, 3H) ppm. ^{13}C NMR (75 MHz, $CDCl_3$, 298 K): δ 72.88, 66.89, 64.95, 59.89, 53.10, 44.56, 30.83, 27.74, 27.52, 26.31, 26.25, 26.10, 14.70 ppm.

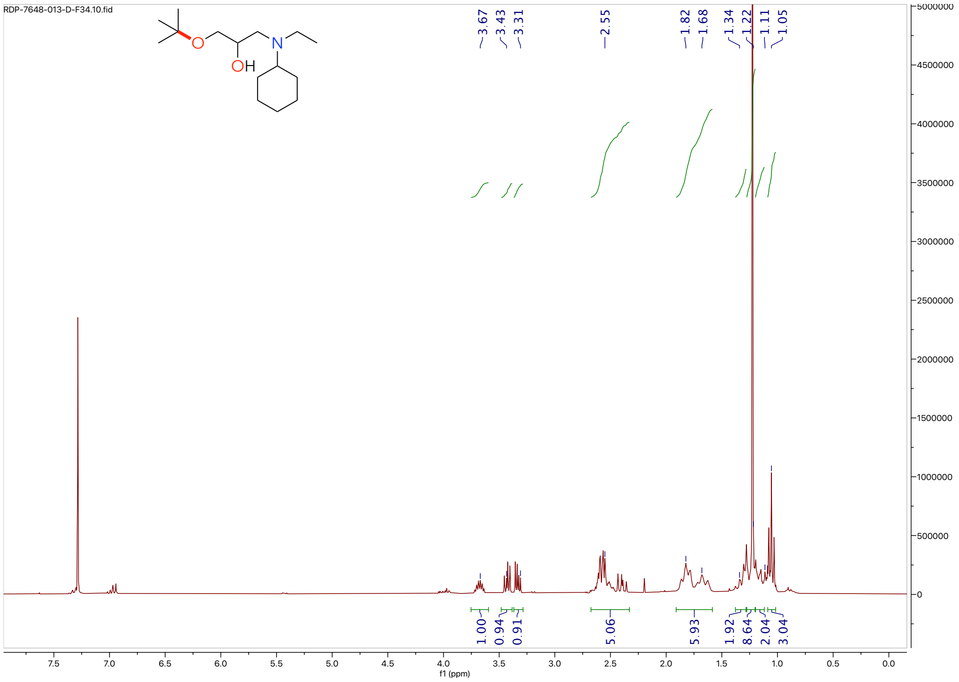

Figure S23. ^{1}H NMR spectrum (300 MHz, $CDCl_3$, 298 K) of 1-(tert-butoxy)-3-(cyclohexyl(ethyl)amino)propan-2-ol.

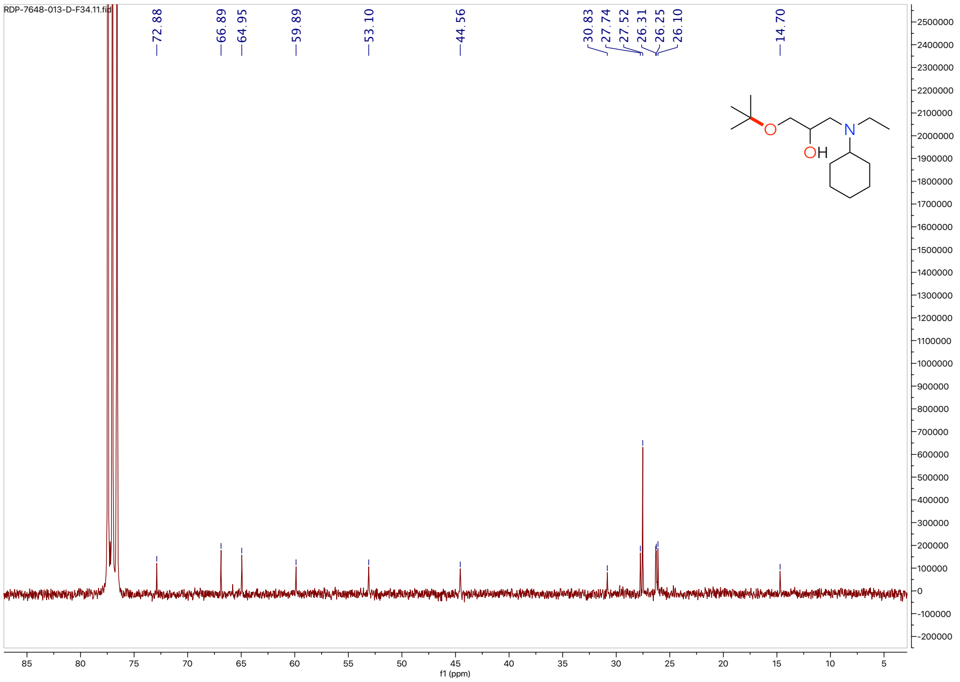

Figure S24. ^{13}C NMR spectrum (75 MHz, $CDCl_3$, 298 K) of 1-(tert-butoxy)-3-(cyclohexyl(ethyl)amino)propan-2-ol.

S6. Thermoplastic Syntheses and Characterization

General procedure for the synthesis of thermoplastic epoxy model materials (GP2): Polymers were prepared by combining a chosen amine and with one equivalent of 2,2'-(((propane-2,2-diylbis(4,1-phenylene))bis(oxy))bis(methylene))bis(oxirane) (BADGE, **6**). These ratios were set for a resultant 1:1 ratio of mols of N-H bonds to epoxide units. Monomers were combined by first melting the desired quantity of 2,2'-(((propane-2,2-diylbis(4,1-phenylene))bis(oxy))bis(methylene))bis(oxirane) and then transferring the resultant liquid to a spin mixer cup before directly adding each liquid amine. This resin mixture was homogenized in a spin mixer cup at 2000 rpm for 10 seconds, and then 3000 rpm for 1 minute and 50 seconds. The resultant solution was added over a scale to 1x1x1 cm cubes in a silicone sheet at desired weights. These polymer cubes were polymerized in a ventilated oven at unique cure schedules, as seen below in specific preparations. Polymer cubes were then directly characterized and used in deconstruction reactions without purification.

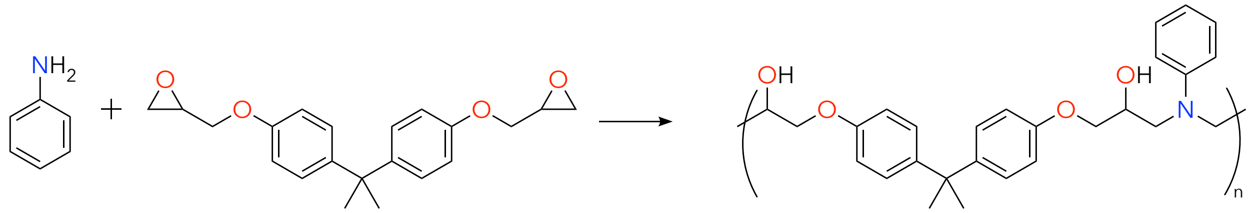

This aromatic thermoplastic was used as a test polymer to show reproducibility in polymer synthesis, and scalability. The series of image in ***Figure S25*** *below shows how we generate a large batch of polymer cubes in one curing event, such that we use the same batch of polymer for this entire paper. We also show representative DSC data (****Figure 42****) for polymer cubes of different masses, to highlight that one cure cycle can be used for materials of different physical sizes.*

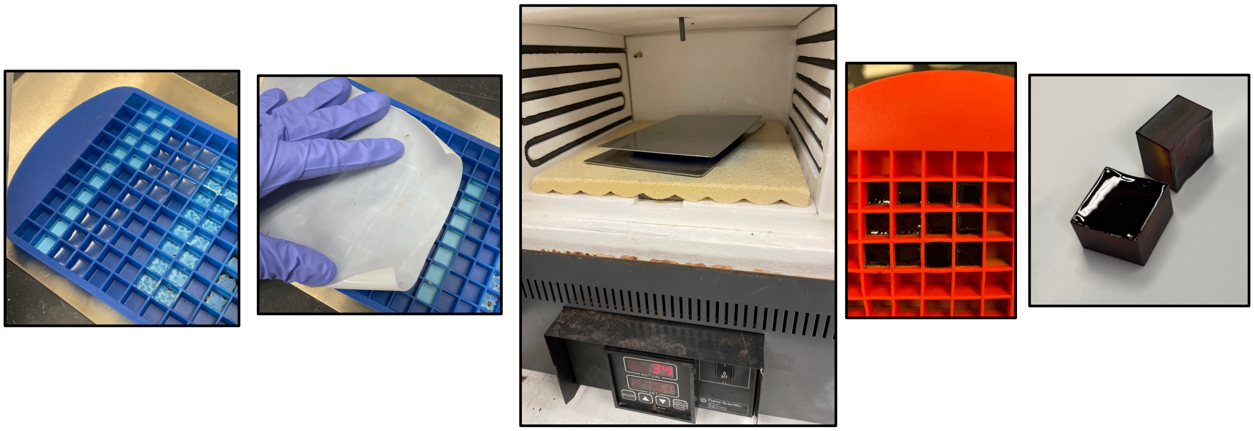

Figure S25. Curing setup for the scalable synthesis of ~200 mg cubes of poly-1-(ethyl(phenyl)amino)-3-(4-(2-(4-(2-hydroxypropoxy)phenyl)propan-2-yl)phenoxy)propan-2-ol (**7**).

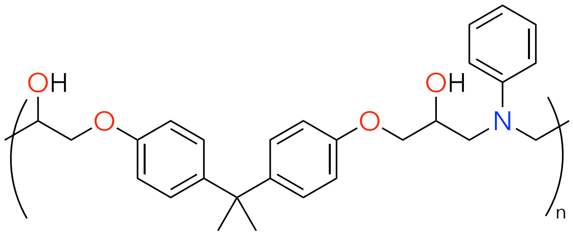

Synthesis of Poly-1-(ethyl(phenyl)amino)-3-(4-(2-(4-(2-hydroxypropoxy)phenyl)propan-2-yl)phenoxy)propan-2-ol (7): Prepared following the general procedure outlined above: Aniline (16.37 g, 176 mmol), BADGE (60 g, 176 mmol). ^{1}H NMR (300 MHz, THF-d_8, 298 K): δ 6.99 (m, 6H), 6.72 (q, 6H), 6.46 (q, 1H), 5.11 (d, 1H), 4.49 (d, 1H), 4.13 (m, 2H), 3.84 (m, 4H), 3.61 (dd, 1H), 3.44 (m, 1H), 3.18 (dd, 1H), 1.49 (s, 5H) ppm. ^{13}C NMR (75 MHz, THF-d_8, 298 K): δ 156.97, 156.92, 148.65, 147.99, 143.16, 143.08, 128.78, 128.70, 127.49, 127.46, 115.79, 113.75, 112.50, 112.05, 70.46, 70.33, 67.77, 67.63, 57.08, 55.45, 41.45, 30.57 ppm.

Polymerization Schedule:
80 °C for 1 hr; 1.5 °C/min ramp to 100 °C; 100 °C for 1 hr; 1.5 °C/min ramp to 120 °C; 120 °C for 1 hr; 1.5 °C/min ramp to 140 °C; 140 °C for 1 hr; 1.5 °C/min ramp to 160 °C; 160 °C for 1 hr; 1.5 °C/min ramp to 180 °C; 180 °C for 10 hr.

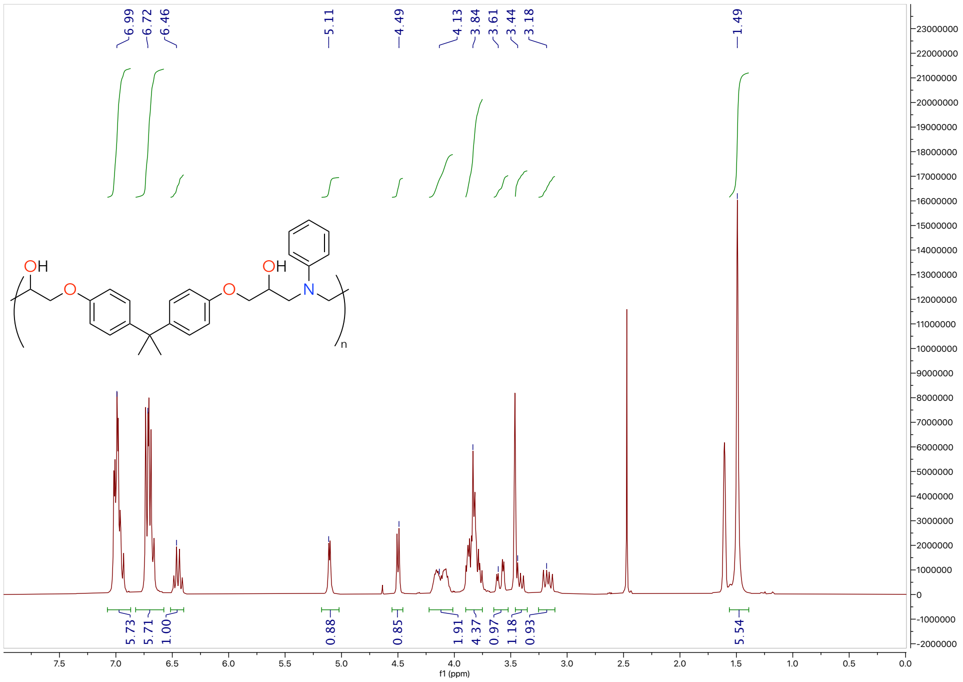

Figure S26. ^{1}H NMR spectrum (300 MHz, THF-d$_8$, 298 K) of poly-1-(ethyl(phenyl)amino)-3-(4-(2-(4-(2-hydroxypropoxy)phenyl)propan-2-yl)phenoxy)propan-2-ol (**7**).

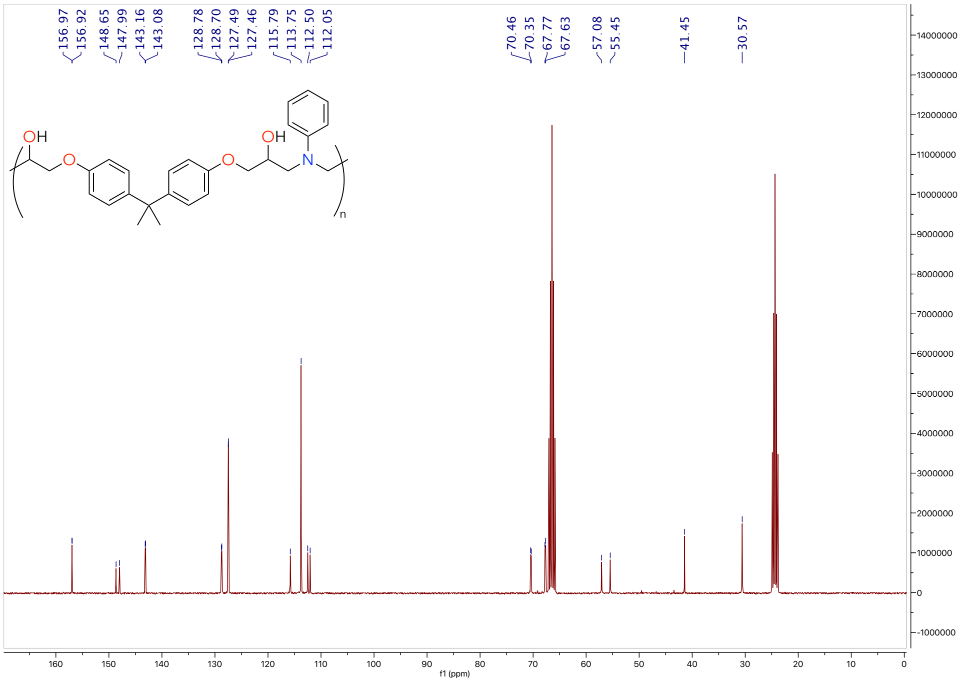

Figure S27. ^{13}C NMR spectrum (75 MHz, THF-d$_8$, 298 K) of poly-1-(ethyl(phenyl)amino)-3-(4-(2-(4-(2-hydroxypropoxy)phenyl)propan-2-yl)phenoxy)propan-2-ol (**7**).

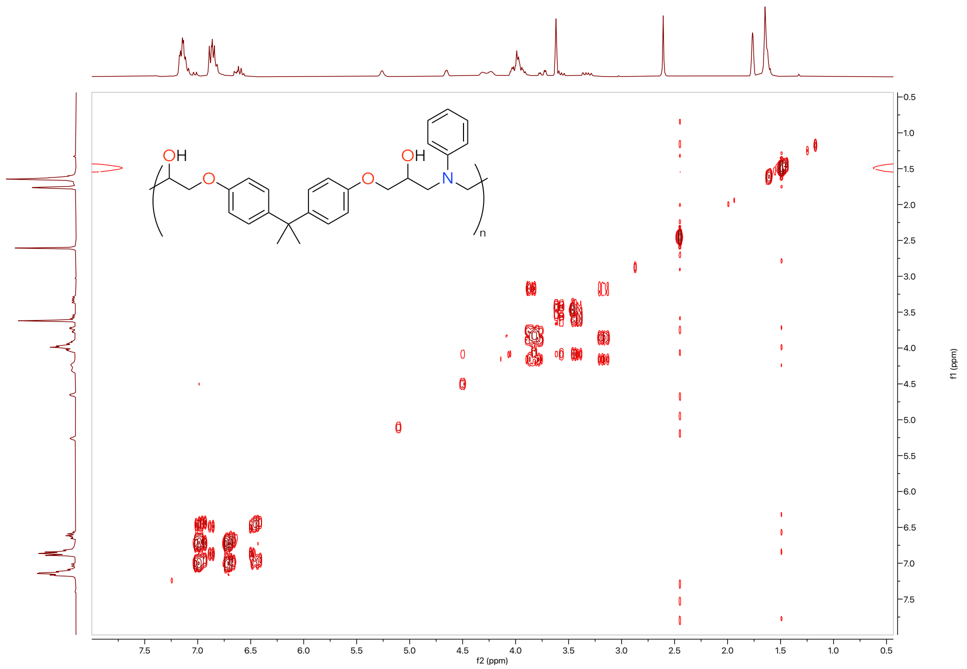

Figure S28. COSY NMR spectrum (300 MHz, THF-d$_8$, 298 K) of poly-1-(ethyl(phenyl)amino)-3-(4-(2-(4-(2-hydroxypropoxy)phenyl)propan-2-yl)phenoxy)propan-2-ol (**7**).

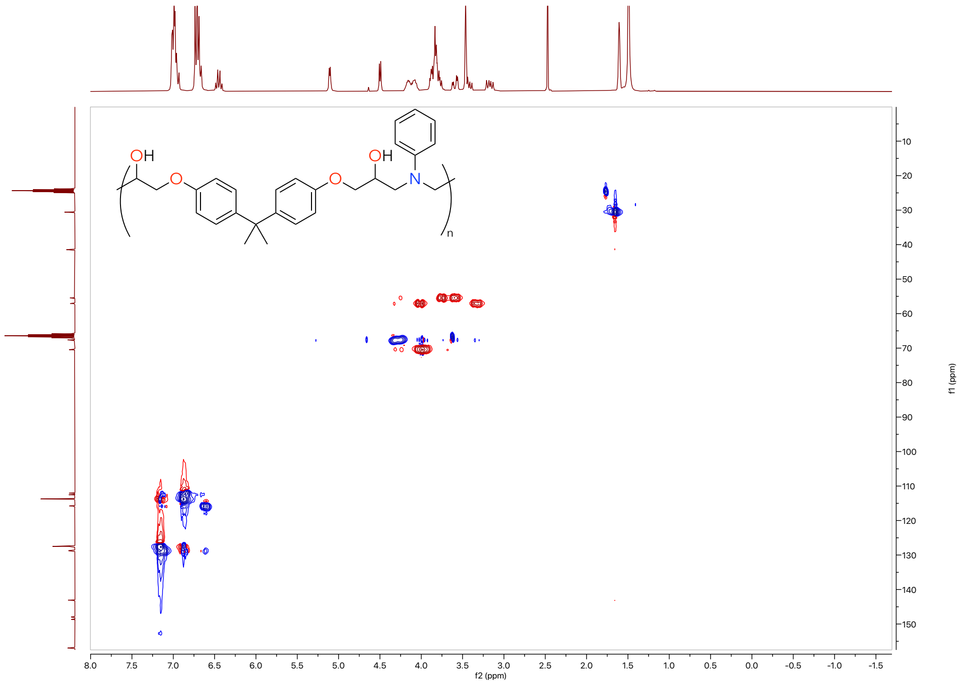

Figure S29. HSQC NMR spectrum (300 MHz/ 75 MHz, THF-d$_8$, 298 K) of poly-1-(ethyl(phenyl)amino)-3-(4-(2-(4-(2-hydroxypropoxy)phenyl)propan-2-yl)phenoxy)propan-2-ol (**7**).

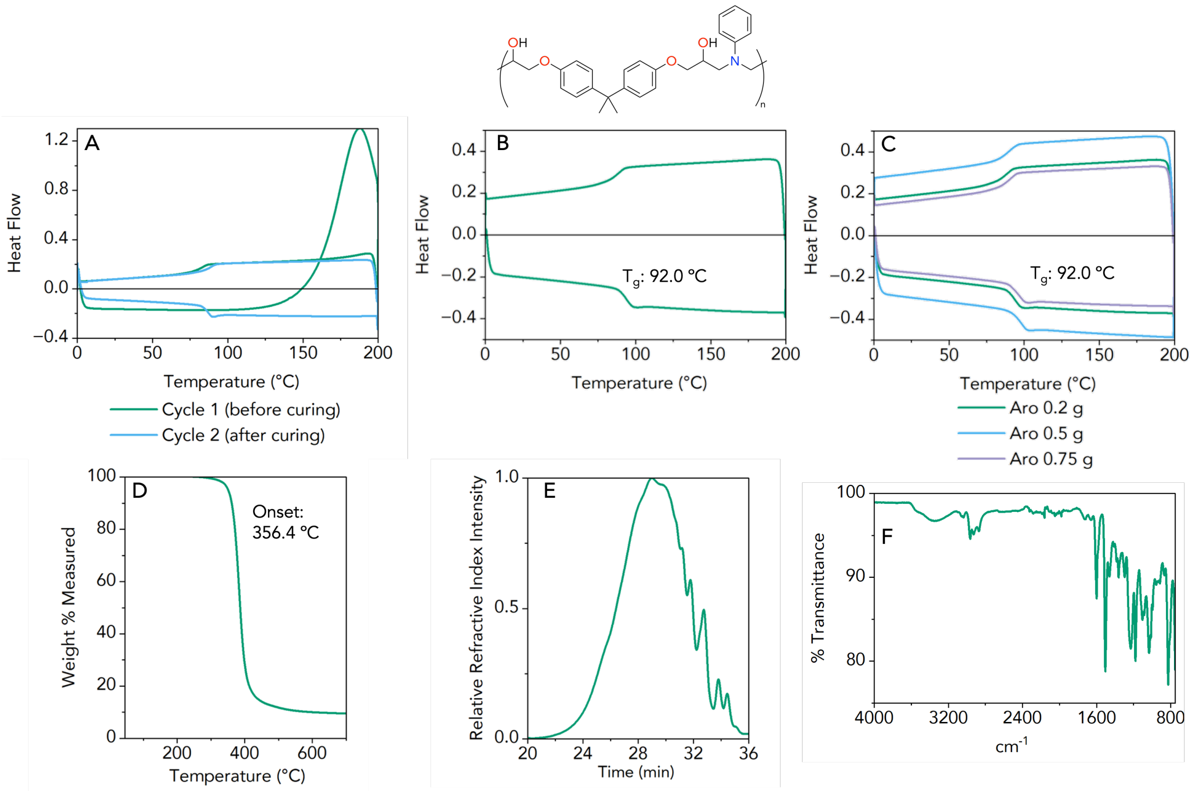

Figure S30. (A) DSC polymerization data before the synthesis of poly-1-(ethyl(phenyl)amino)-3-(4-(2-(4-(2-hydroxypropoxy)phenyl)propan-2-yl)phenoxy)propan-2-ol (**7**). (**B**) DSC analysis **7**. (**C**) DSC analysis of 0.2, 0.5, and 0.75g samples of **7**. **(D)** TGA analysis of **7**. (**E**) GPC analysis of **7**. (**F**) IR analysis of **7**.

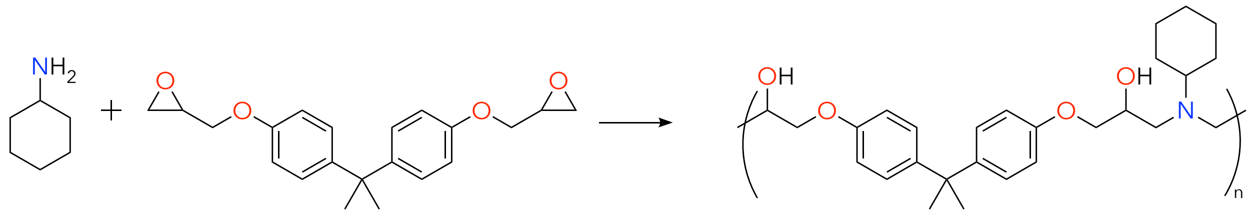

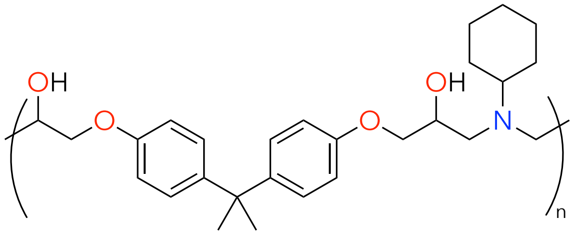

Synthesis of Poly-1-(cyclohexyl(ethyl)amino)-3-(4-(2-(4-(2-hydroxypropoxy)phenyl)propan-2-yl)phenoxy)propan-2-ol (8): Prepared following the general procedure outlined above: Aniline (8.72 g, 88 mmol), BADGE (30 g, 88 mmol). ^{1}H NMR (300 MHz, THF-d_8, 298 K): δ 6.99 (t, 4H), 6.67 (d, 4H), 4.18 (s, 1H), 4.04 (dd, 1H), 3.81 (s, 4H), 3.71 (t, 1H), 3.12 (m, 1H), 2.71 (m, 2H), 2.48 (m, 3H), 1.72 (t, 3H), 1.50 (s, 6H), 1.10 (m, 5H) ppm. ^{13}C NMR (75 MHz, THF-d_8, 298 K): δ 157.90, 157.88, 157.53, 157.51, 144.10, 144.01, 143.67, 143.58, 128.23, 128.16, 114.46, 71.54, 71.51, 69.90, 69.77, 69.20, 62.51, 62.31, 55.98, 55.41, 50.31, 44.20, 42.18, 31.35, 31.31, 30.14, 29.74, 29.33, 27.06, 26.95 ppm.

<u>Polymerization Schedule</u>: 40 °C for 1 hr; 1.5 °C/min ramp to 60 °C; 60 °C for 1 hr; 1.5 °C/min ramp to 80 °C; 80 °C for 1 hr; 1.5 °C/min ramp to 100 °C; 100 °C for 1 hr; 1.5 °C/min ramp to 120 °C; 120 °C for 1 hr; 1.5 °C/min ramp to 140 °C; 140 °C for 10 hr.

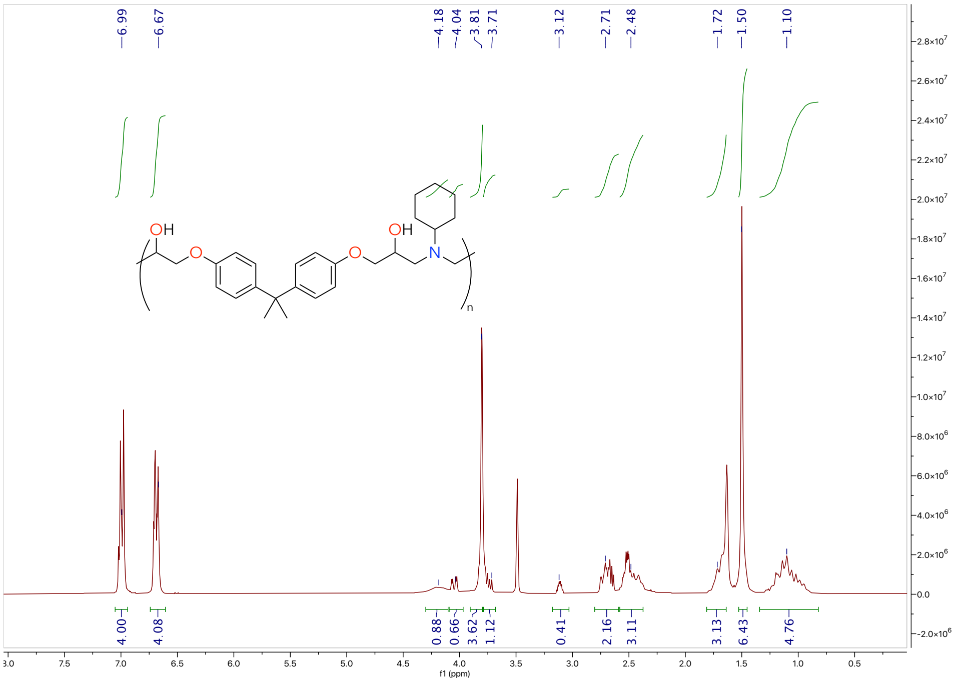

Figure S31. ^{1}H NMR spectrum (300 MHz, THF-d$_8$, 298 K) of poly-1-(cyclohexyl(ethyl)amino)-3-(4-(2-(4-(2-hydroxypropoxy)phenyl)propan-2-yl)phenoxy)propan-2-ol (**8**).

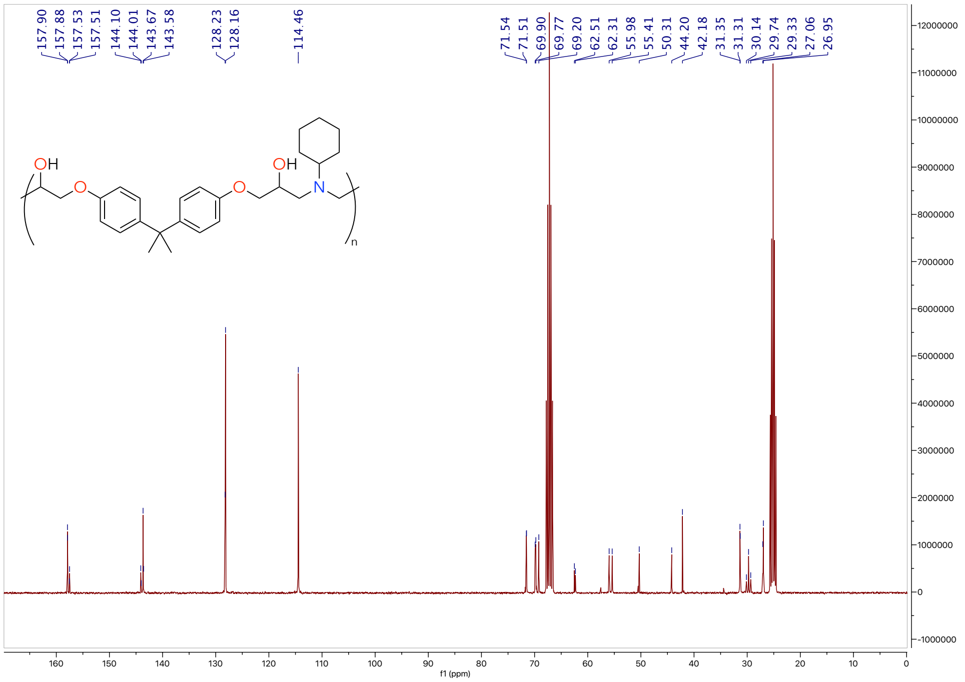

Figure S32. ^{13}C NMR spectrum (75 MHz, THF-d$_8$, 298 K) of poly-1-(cyclohexyl(ethyl)amino)-3-(4-(2-(4-(2-hydroxypropoxy)phenyl)propan-2-yl)phenoxy)propan-2-ol (**8**).

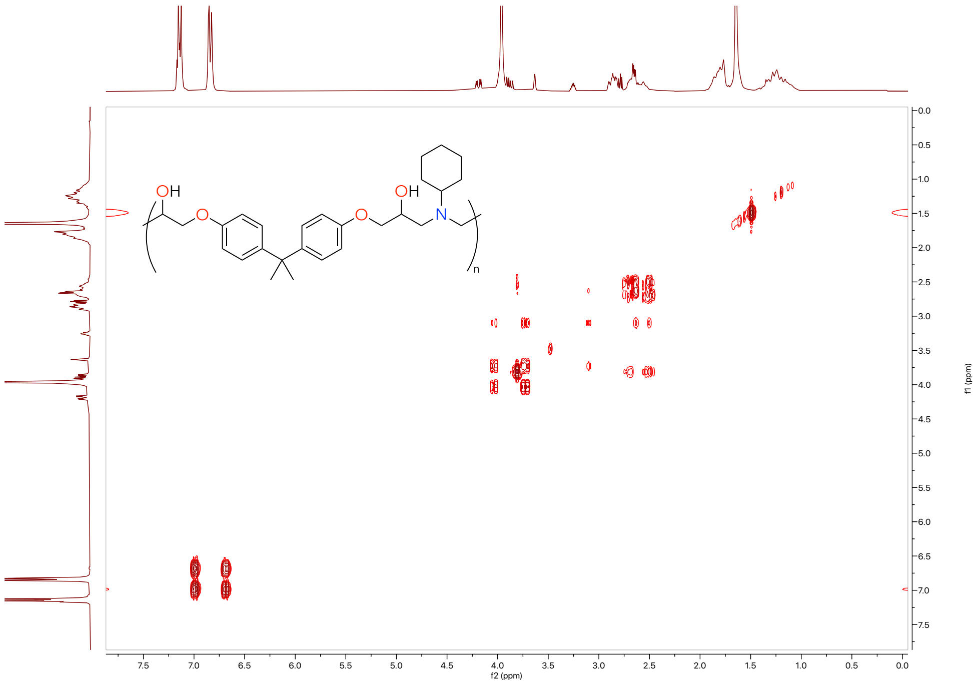

Figure S33. COSY NMR spectrum (300 MHz, THF-d_8, 298 K) of poly-1-(cyclohexyl(ethyl)amino)-3-(4-(2-(4-(2-hydroxypropoxy)phenyl)propan-2-yl)phenoxy)propan-2-ol (**8**).

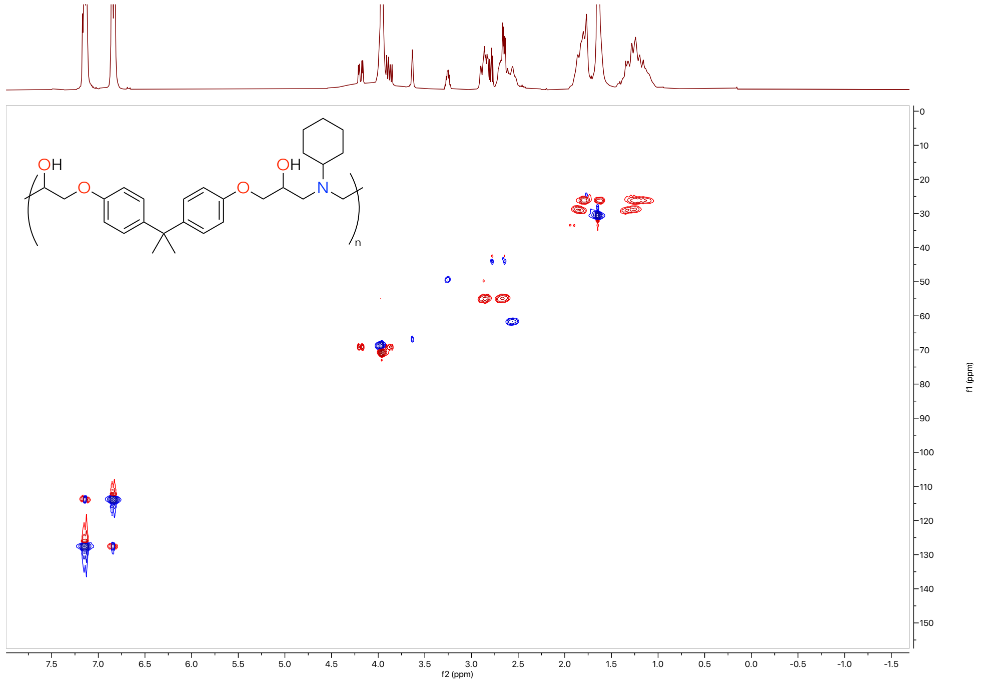

Figure S34. HSQC NMR spectrum (300 MHz/ 75 MHz, THF-d_8, 298 K) of poly-1-(cyclohexyl(ethyl)amino)-3-(4-(2-(4-(2-hydroxypropoxy)phenyl)propan-2-yl)phenoxy)propan-2-ol (**8**).

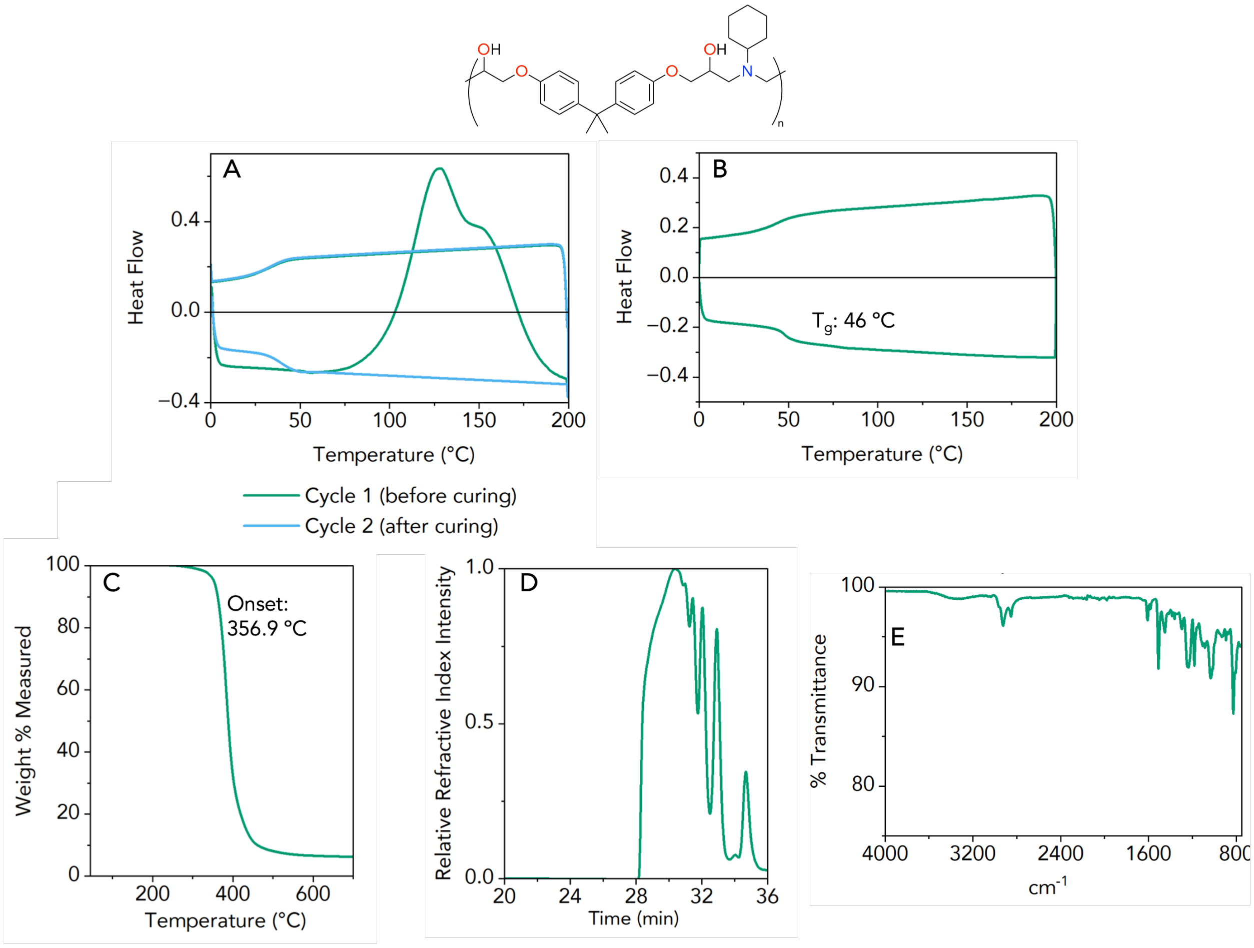

Figure S35. **(A)** DSC polymerization data before the synthesis of poly-1-(cyclohexyl(ethyl)amino)-3-(4-(2-(4-(2-hydroxypropoxy)phenyl)propan-2-yl)phenoxy)propan-2-ol (**8**). (**B**) DSC analysis **8**. (**C**) TGA analysis of **8**. (**D**) GPC analysis of **8**. (**E**) IR analysis of **8**.

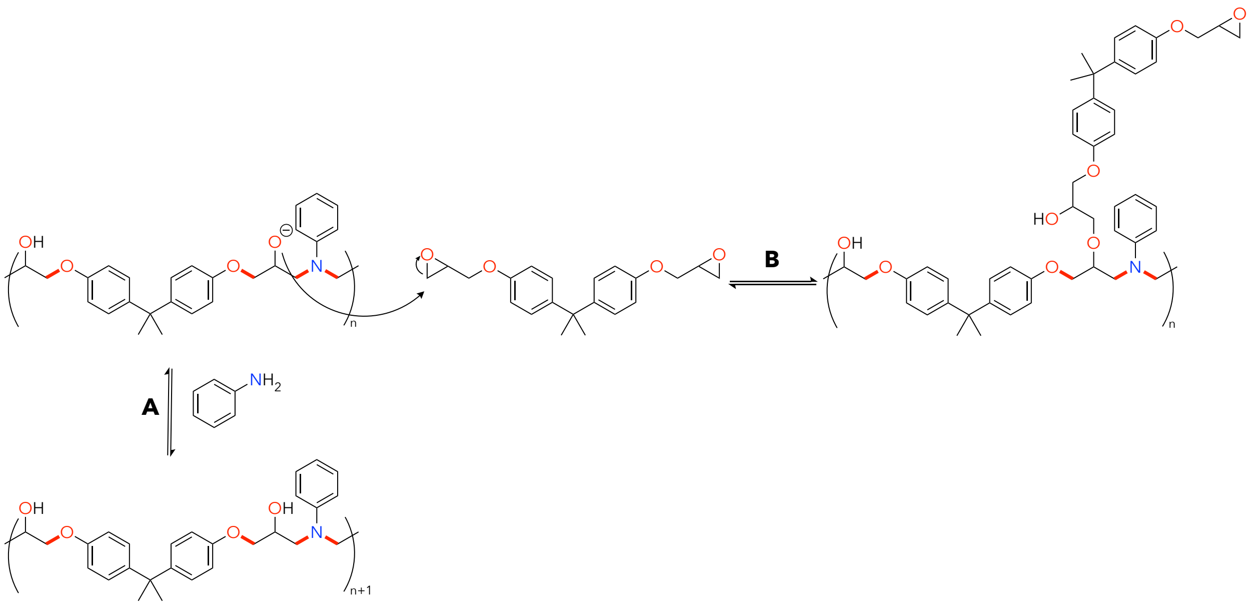

Figure S36. A proposed mechanism for the unexpected crosslinking of aromatic thermoplastic **6**. Typical polymerization is reflected in pathway **A**, while the start of crosslinking through the alcohol in a growing polymer chain is shown in **B**.

*We synthesized three thermoplastics with mixed ratios of aniline and cyclohexylamine curing agents. These compounds are **9-11**, with schemes, written preparations, and structural images that reflect these different amine ratios. NMR spectra for each polymer also reflect these ratios in ways that are more challenging to see in thermal data. As a result of the random copolymer mixtures in these cases, integrals from proton NMR spectra are not represented in typical whole proton numbers.*

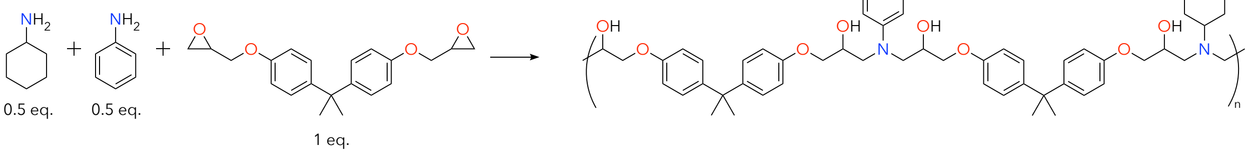

Synthesis of Poly-1-(cyclohexyl(ethyl)amino)-3-(4-(2-(4-(2-hydroxy-3-((2-hydroxy-3-(4-(2-(4-(2-hydroxypropoxy)phenyl)propan-2-yl)phenoxy)propyl)(phenyl)amino)propoxy)phenyl)propan-2-yl)phenoxy)propan-2-ol (9): Prepared following the general procedure outlined above: Aniline (1.36 g, 15 mmol), cyclohexylamine (1.45 g, 15 mmol), BADGE (10 g, 30 mmol). ^{1}H NMR (300 MHz, THF-d_8, 298 K): δ 7.01 (d, 10H), 6.70 (m, 10H), 6.46 (q, 1H), 5.11 (s, 1H), 4.50 (s, 1H), 4.14 (m, 4H), 3.83 (m, 10H), 3.60 (dd, 1H), 3.43 (m, 1H), 3.18 (m, 1H), 2.68 (m, 2H), 2.50 (m, 2H), 2.38 (s, 1H), 1.72 (s, 4H), 1.50 (s, 12H), 1.10 (m, 4H) ppm. ^{13}C NMR (75 MHz, THF-d_8, 298 K): δ 157.13, 156.98, 156.91, 156.75, 148.64, 147.99, 143.30, 143.18, 143.15, 143.11, 143.08, 142.90, 142.88, 128.77, 128.69, 127.46, 127.39, 115.78, 113.74, 113.69, 112.49, 112.04, 70.77, 70.46, 70.35, 69.12, 69.00, 68.44, 67.76, 67.62, 61.74, 61.55, 57.09, 55.45, 55.20, 54.63, 49.54, 43.43, 41.43, 30.57, 29.36, 28.96, 26.28, 26.18 ppm.

Polymerization Schedule:
80 °C for 1 hr; 1.5 °C/min ramp to 100 °C; 100 °C for 1 hr; 1.5 °C/min ramp to 130 °C; 130 °C for 10 hr.

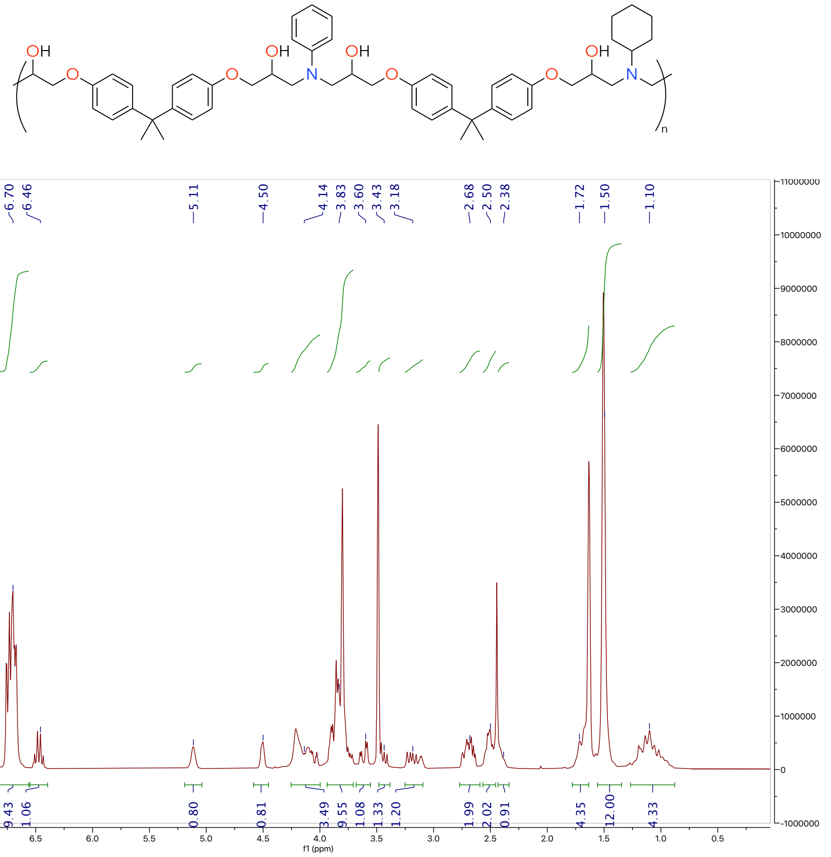

Figure S37. ^{1}H NMR spectrum (300 MHz, THF-d_8, 298 K) of poly-1-(cyclohexyl(ethyl)amino)-3-(4-(2-(4-(2-hydroxy-3-((2-hydroxy-3-(4-(2-(4-(2-hydroxypropoxy)phenyl)propan-2-yl)phenoxy)propyl)(phenyl)amino)propoxy)phenyl)propan-2-yl)phenoxy)propan-2-ol, as synthesized with 0.5 eq. of cyclohexylamine and 0.5 eq. of aniline (**9**).

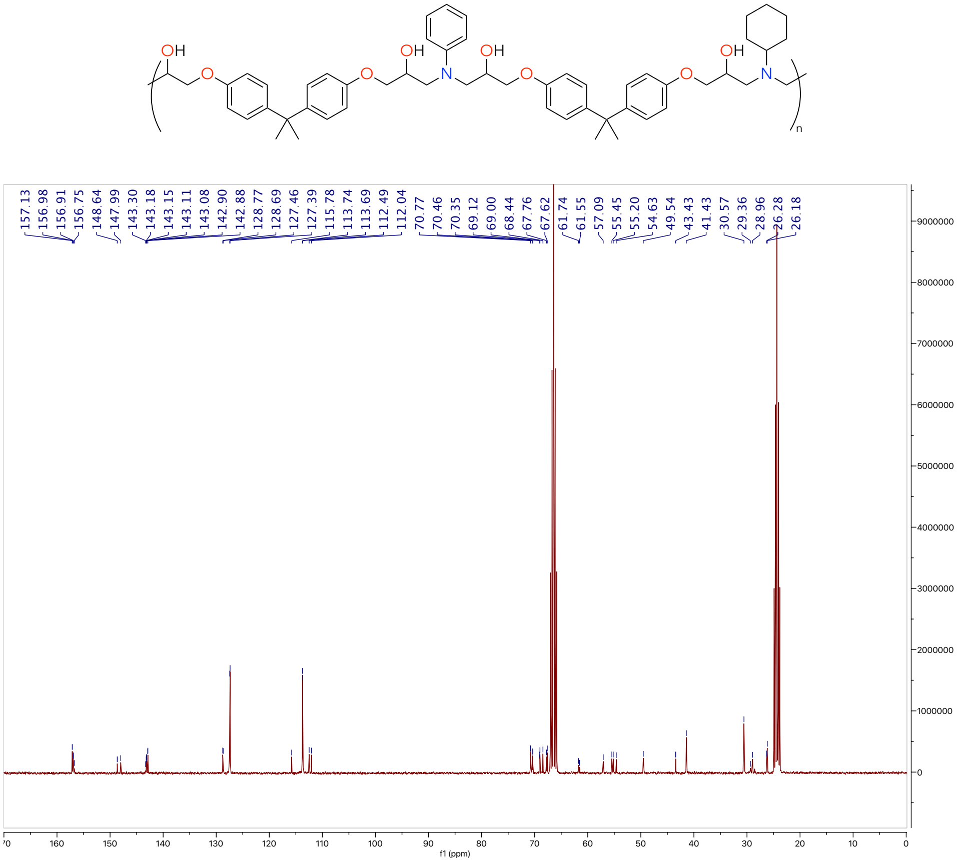

Figure S38. ^{13}C NMR spectrum (75 MHz, THF-d_8, 298 K) of poly-1-(cyclohexyl(ethyl)amino)-3-(4-(2-(4-(2-hydroxy-3-((2-hydroxy-3-(4-(2-(4-(2-hydroxypropoxy)phenyl)propan-2-yl)phenoxy)propyl)(phenyl)amino)propoxy)phenyl)propan-2-yl)phenoxy)propan-2-ol, as synthesized with 0.5 eq. of cyclohexylamine and 0.5 eq. of aniline (**9**).

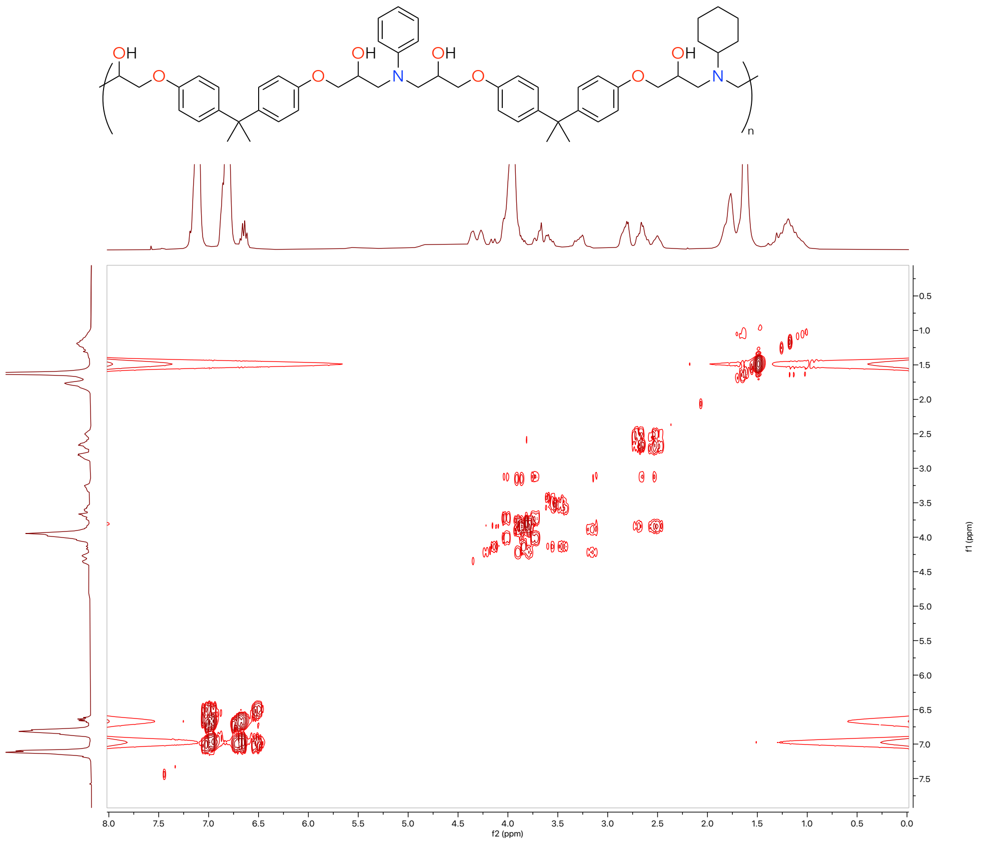

Figure S39. COSY NMR spectrum (300 MHz, THF-d_8, 298 K) of poly-1-(cyclohexyl(ethyl)amino)-3-(4-(2-(4-(2-hydroxy-3-((2-hydroxy-3-(4-(2-(4-(2-hydroxypropoxy)phenyl)propan-2-yl)phenoxy)propyl)(phenyl)amino)propoxy)phenyl)propan-2-yl)phenoxy)propan-2-ol, as synthesized with 0.5 eq. of cyclohexylamine and 0.5 eq. of aniline (**9**).

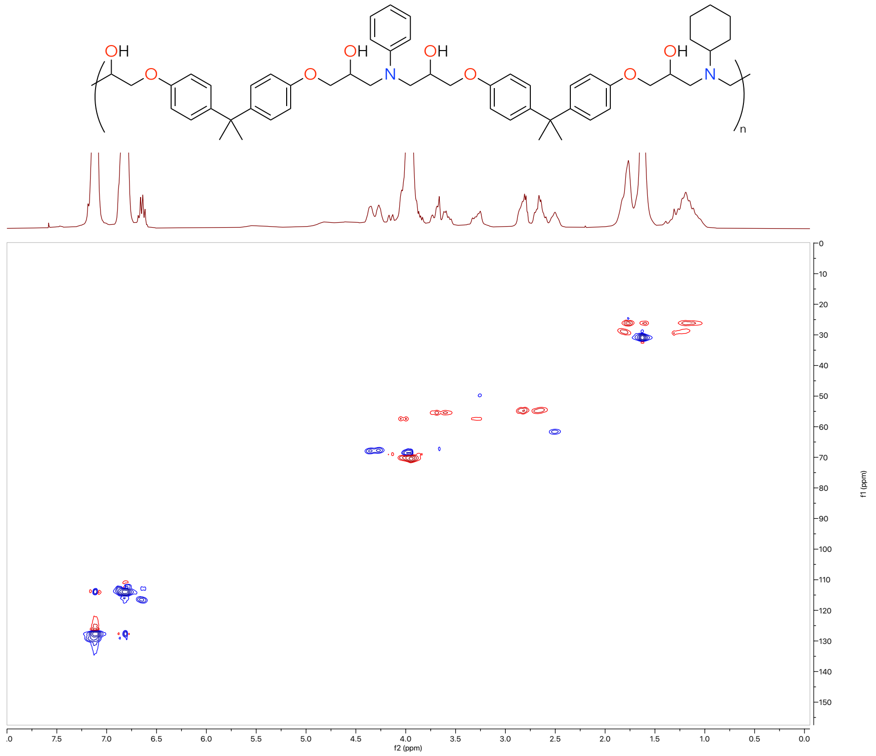

Figure S40. HSQC NMR spectrum (300 MHz/ 75 MHz, THF-d_8, 298 K) of poly-1-(cyclohexyl(ethyl)amino)-3-(4-(2-(4-(2-hydroxy-3-((2-hydroxy-3-(4-(2-(4-(2-hydroxypropoxy)phenyl)propan-2-yl)phenoxy)propyl)(phenyl)amino)propoxy)phenyl)propan-2-yl)phenoxy)propan-2-ol, as synthesized with 0.5 eq. of cyclohexylamine and 0.5 eq. of aniline (**9**).

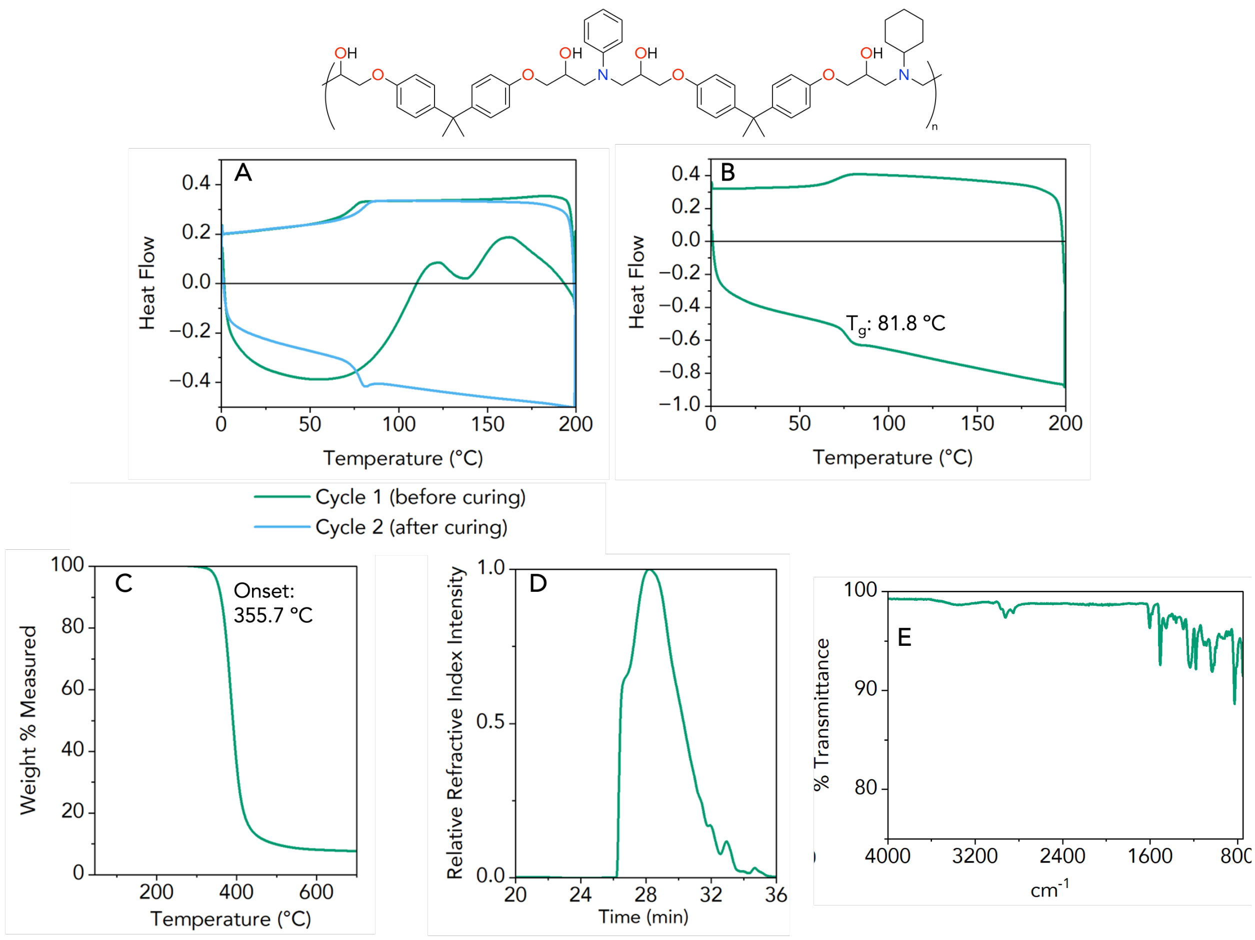

Figure S41. (**A**) DSC polymerization data before the synthesis of poly-1-(cyclohexyl(ethyl)amino)-3-(4-(2-(4-(2-hydroxy-3-((2-hydroxy-3-(4-(2-(4-(2-hydroxypropoxy)phenyl)propan-2-yl)phenoxy)propyl)(phenyl)amino)propoxy)phenyl)propan-2-yl)phenoxy)propan-2-ol, as synthesized with 0.5 eq. of cyclohexylamine and 0.5 eq. of aniline (**9**). (**B**) DSC analysis of **9**. (**C**) TGA analysis of **9**. (**D**) GPC analysis of **9**. (**E**) IR analysis of **9**.

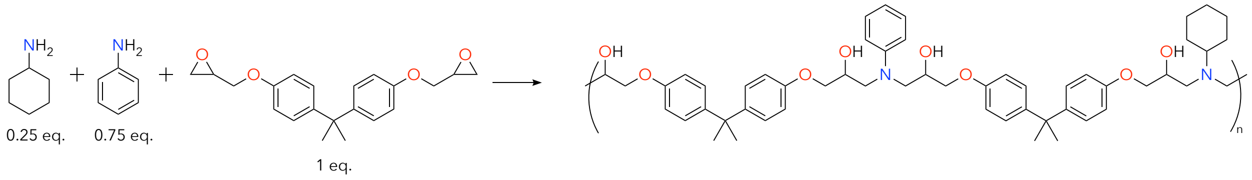

Synthesis of Poly-1-(cyclohexyl(ethyl)amino)-3-(4-(2-(4-(2-hydroxy-3-((2-hydroxy-3-(4-(2-(4-(2-hydroxypropoxy)phenyl)propan-2-yl)phenoxy)propyl)(phenyl)amino)propoxy)phenyl)propan-2-yl)phenoxy)propan-2-ol (10): Prepared following the general procedure outlined above: Aniline (4.10 g, 0.044 mol), cyclohexylamine (1.46 g, 0.015 mol), BADGE (20.00 g, 0.059 mol) (**10**).

Polymerization Schedule:
80 ºC for 1 hr; 1.5 ºC/min ramp to 100ºC; 100 ºC for 1 hr; 1.5 ºC/min ramp to 120 ºC; 120 ºC for 1 hr; 1.5 ºC/min ramp to 140 ºC; 140 ºC for 1 hr; 1.5 ºC/min ramp to 160 ºC; 160 ºC for 1 hr; 1.5 ºC/min ramp to 180 ºC; 180 ºC for 10 hr.

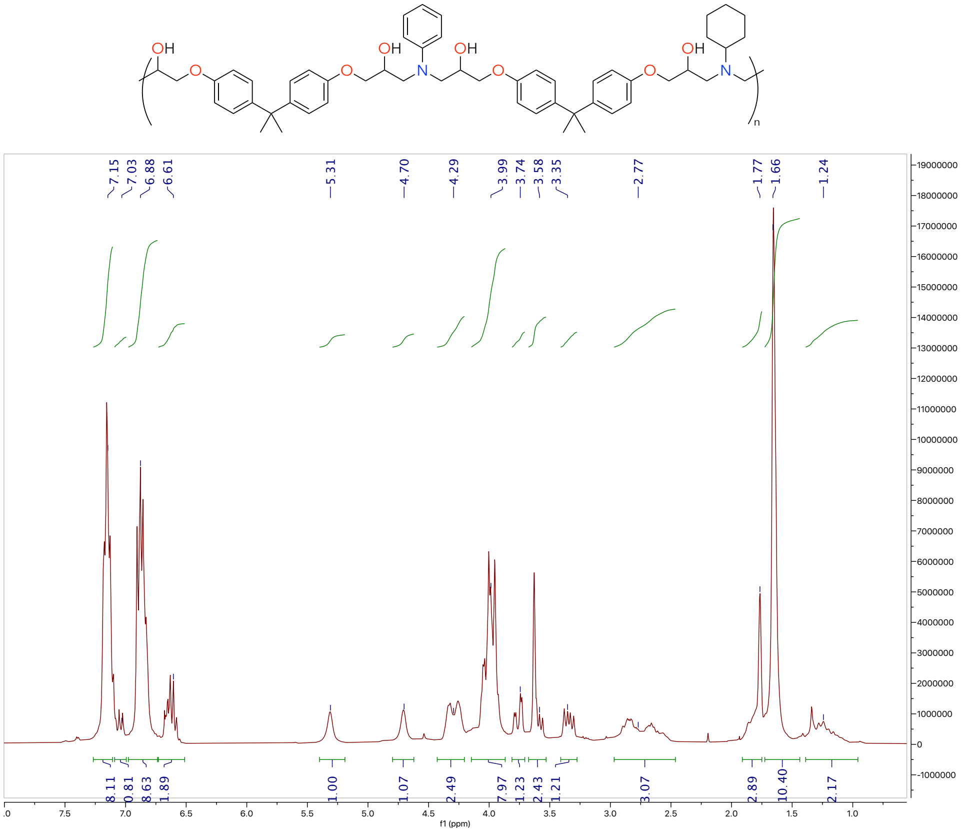

Figure S42. ^{1}H NMR spectrum (300 MHz, THF-d_8, 298 K) of poly-1-(cyclohexyl(ethyl)amino)-3-(4-(2-(4-(2-hydroxy-3-((2-hydroxy-3-(4-(2-(4-(2-hydroxypropoxy)phenyl)propan-2-yl)phenoxy)propyl)(phenyl)amino)propoxy)phenyl)propan-2-yl)phenoxy)propan-2-ol, as synthesized with 0.25 eq. of cyclohexylamine and 0.75 eq. of aniline (**10**).

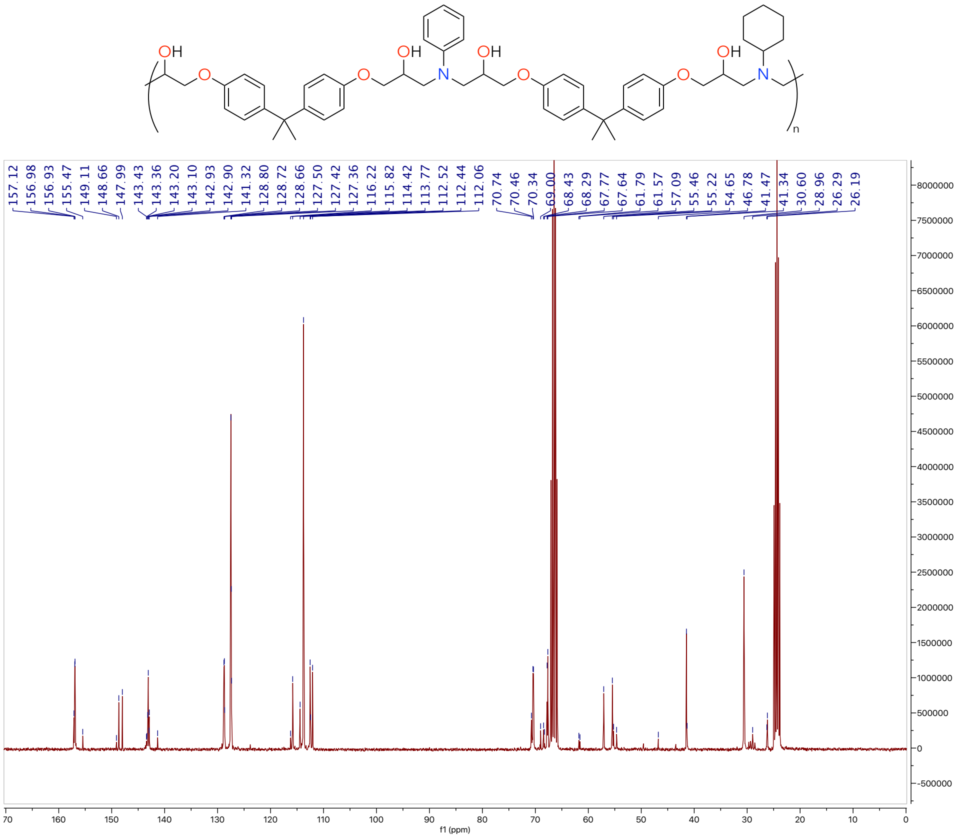

Figure S43. ^{13}C NMR spectrum (75 MHz, THF-d$_8$, 298 K) of poly-1-(cyclohexyl(ethyl)amino)-3-(4-(2-(4-(2-hydroxy-3-((2-hydroxy-3-(4-(2-(4-(2-hydroxypropoxy)phenyl)propan-2-yl)phenoxy)propyl)(phenyl)amino)propoxy)phenyl)propan-2-yl)phenoxy)propan-2-ol, as synthesized with 0.25 eq. of cyclohexylamine and 0.75 eq. of aniline (**10**).

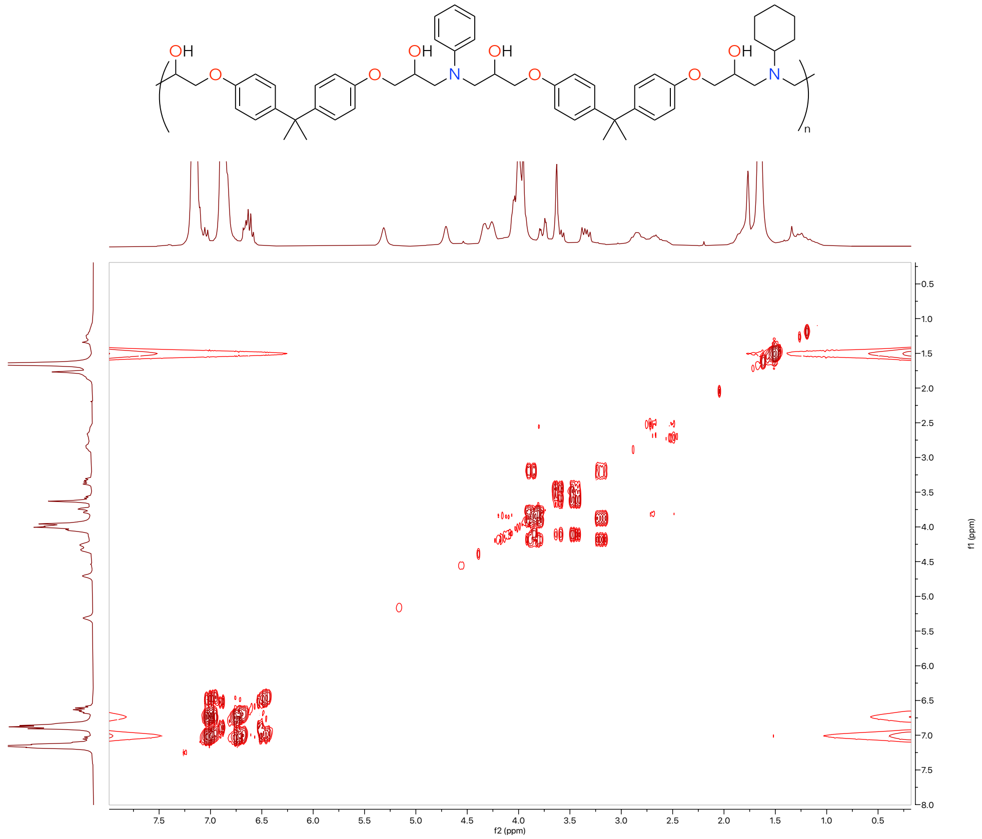

Figure S44. COSY NMR spectrum (300 MHz, THF-d_8, 298 K) of poly-1-(cyclohexyl(ethyl)amino)-3-(4-(2-(4-(2-hydroxy-3-((2-hydroxy-3-(4-(2-(4-(2-hydroxypropoxy)phenyl)propan-2-yl)phenoxy)propyl)(phenyl)amino)propoxy)phenyl)propan-2-yl)phenoxy)propan-2-ol, as synthesized with 0.25 eq. of cyclohexylamine and 0.75 eq. of aniline (**10**).

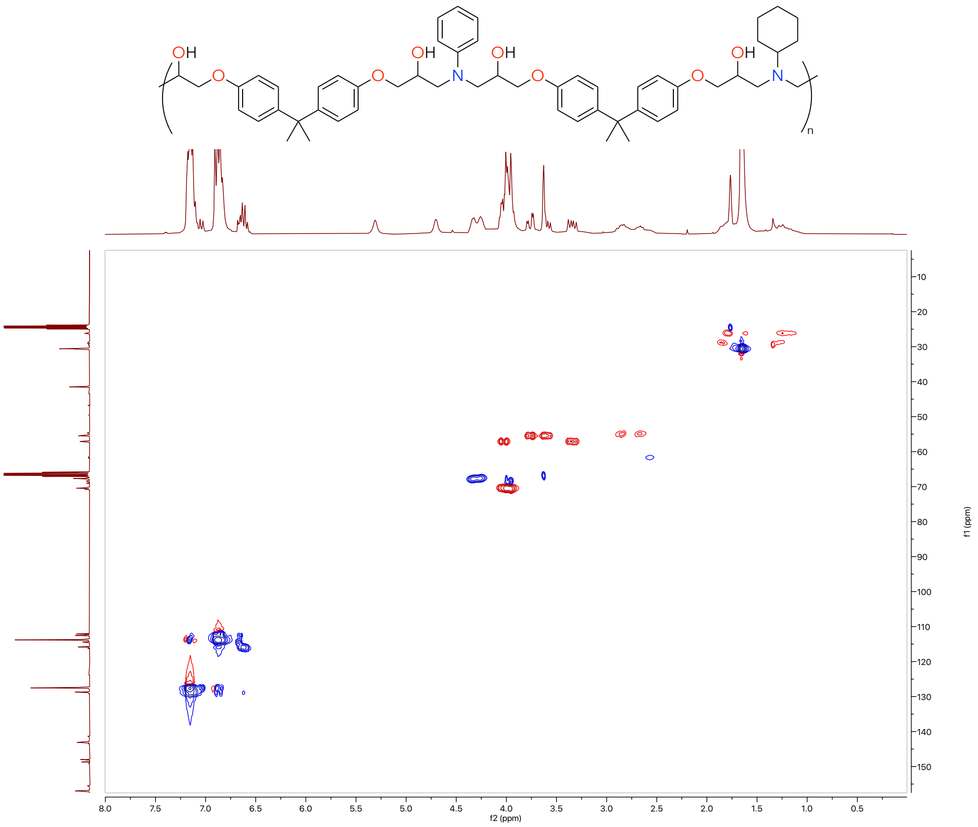

Figure S45. HSQC NMR spectrum (300 MHz, THF-d_8, 298 K) of poly-1-(cyclohexyl(ethyl)amino)-3-(4-(2-(4-(2-hydroxy-3-((2-hydroxy-3-(4-(2-(4-(2-hydroxypropoxy)phenyl)propan-2-yl)phenoxy)propyl)(phenyl)amino)propoxy)phenyl)propan-2-yl)phenoxy)propan-2-ol, as synthesized with 0.25 eq. of cyclohexylamine and 0.75 eq. of aniline (**10**).

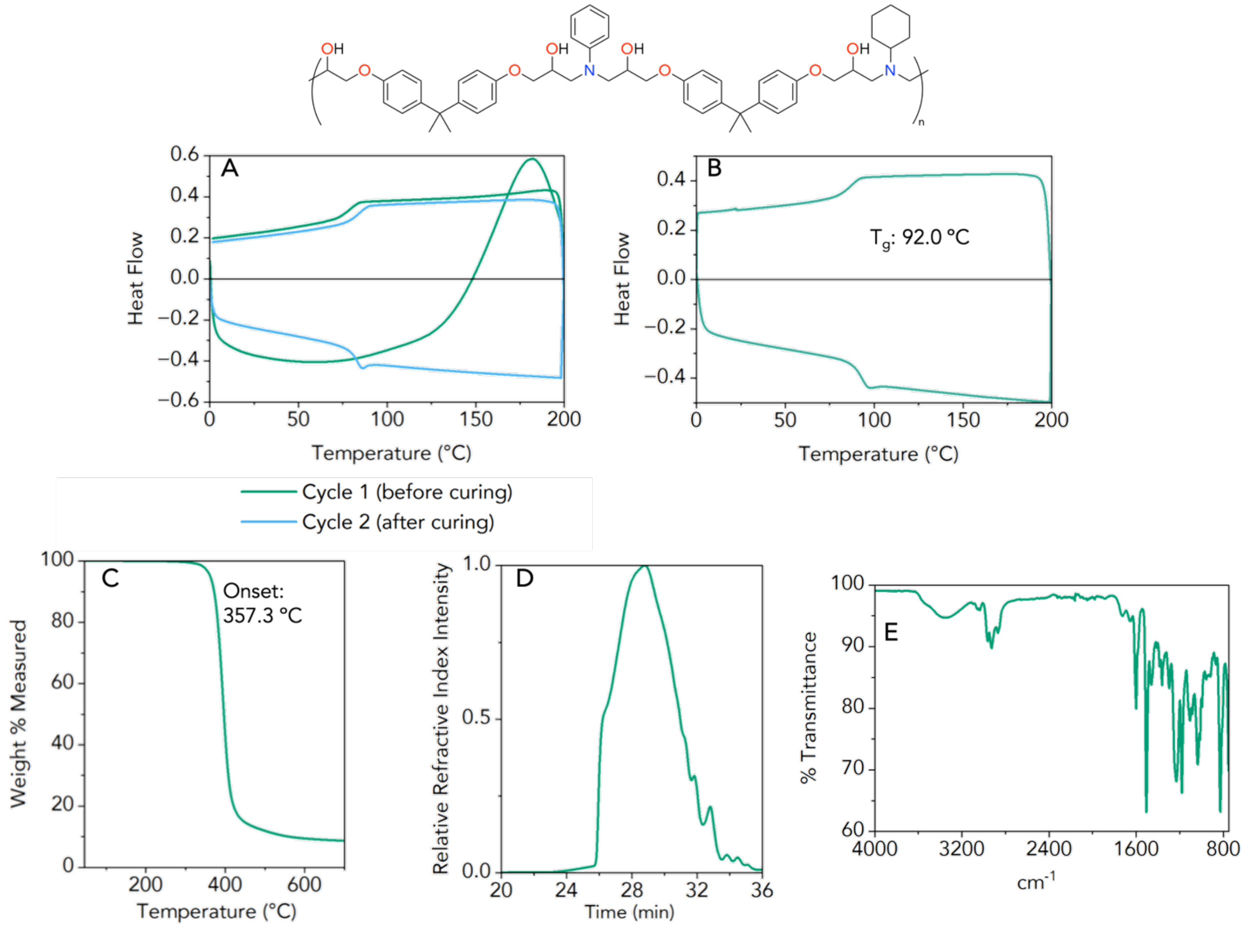

Figure S46. (**A**) DSC polymerization data before the synthesis of poly-1-(cyclohexyl(ethyl)amino)-3-(4-(2-(4-(2-hydroxy-3-((2-hydroxy-3-(4-(2-(4-(2-hydroxypropoxy)phenyl)propan-2-yl)phenoxy)propyl)(phenyl)amino)propoxy)phenyl)propan-2-yl)phenoxy)propan-2-ol, as synthesized with 0.25 eq. of cyclohexylamine and 0.75 eq. of aniline (**10**). (**B**) DSC analysis of **10**. (**C**) TGA analysis of **10**. (**D**) GPC analysis of **10**. (**E**) IR analysis of **10**.

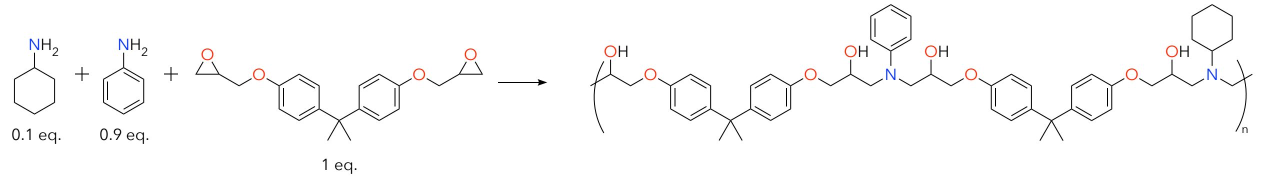

Synthesis of Poly-1-(cyclohexyl(ethyl)amino)-3-(4-(2-(4-(2-hydroxy-3-((2-hydroxy-3-(4-(2-(4-(2-hydroxypropoxy)phenyl)propan-2-yl)phenoxy)propyl)(phenyl)amino)propoxy)phenyl)propan-2-yl)phenoxy)propan-2-ol (11): Prepared following the general procedure outlined above: Aniline (4.93 g, 0.053 mol), cyclohexylamine (0.60 g, 0.006 mol), BADGE (20.00 g, 0.059 mol).

<u>Polymerization Schedule:</u>
80 °C for 1 hr; 1.5 °C /min ramp to 100 °C; 100 °C for 1 hr; 1.5 °C /min ramp to 120 °C; 120 °C for 1 hr; 1.5 °C /min ramp to 140 °C; 140 °C for 1 hr; 1.5 °C /min ramp to 160 °C; 160 °C for 1 hr; 1.5 °C /min ramp to 180 °C; 180 °C for 10 hr.

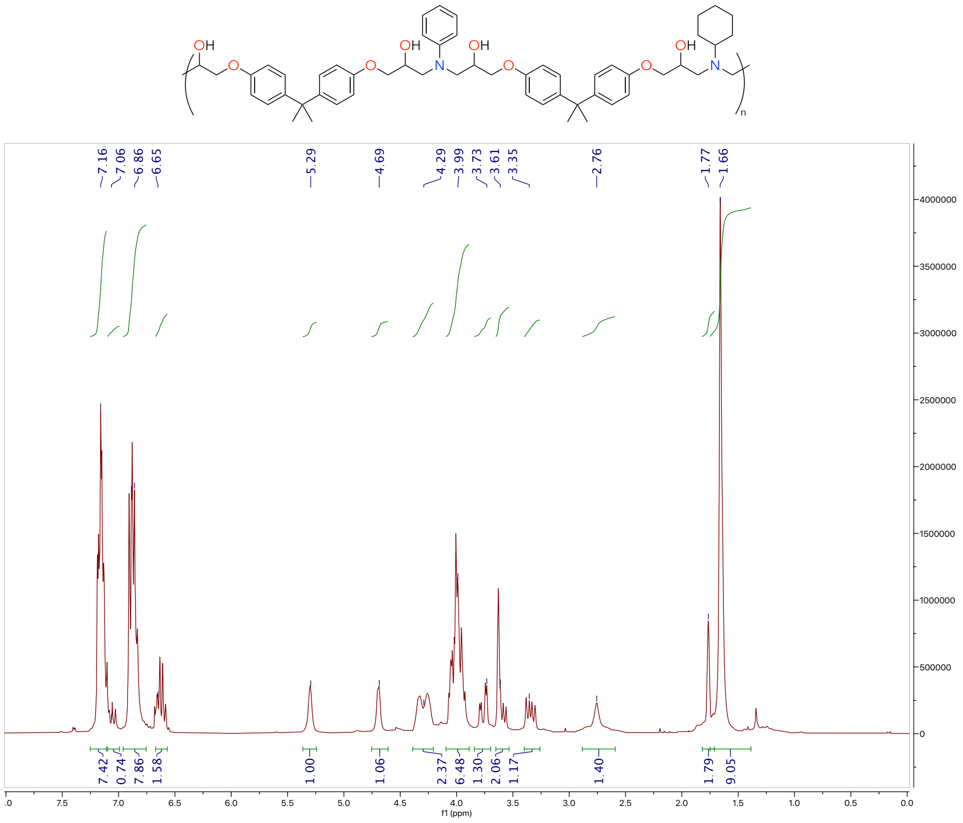

Figure S47. ^{1}H NMR spectrum (300 MHz, THF-d$_8$, 298 K) of poly-1-(cyclohexyl(ethyl)amino)-3-(4-(2-(4-(2-hydroxy-3-((2-hydroxy-3-(4-(2-(4-(2-hydroxypropoxy)phenyl)propan-2-yl)phenoxy)propyl)(phenyl)amino)propoxy)phenyl)propan-2-yl)phenoxy)propan-2-ol, as synthesized with 0.1 eq. of cyclohexylamine and 0.9 eq. of aniline (**11**).

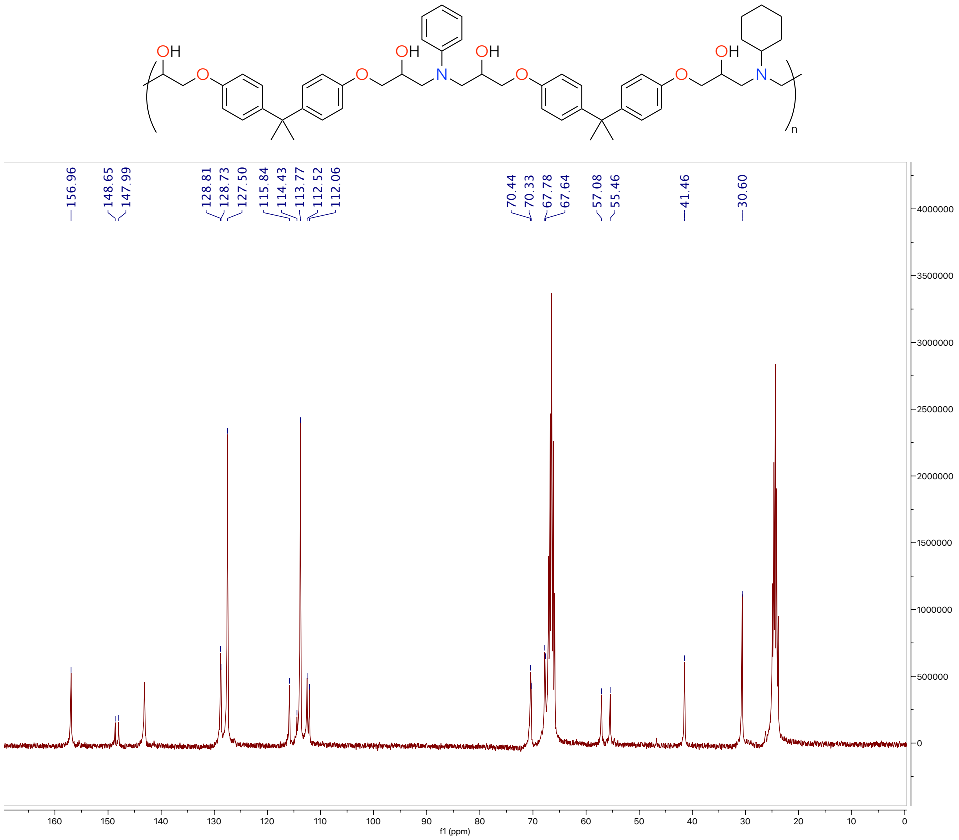

Figure S48. ^{13}C NMR spectrum (75 MHz, THF-d$_8$, 298 K) of poly-1-(cyclohexyl(ethyl)amino)-3-(4-(2-(4-(2-hydroxy-3-((2-hydroxy-3-(4-(2-(4-(2-hydroxypropoxy)phenyl)propan-2-yl)phenoxy)propyl)(phenyl)amino)propoxy)phenyl)propan-2-yl)phenoxy)propan-2-ol, as synthesized with 0.1 eq. of cyclohexylamine and 0.9 eq. of aniline (**11**).

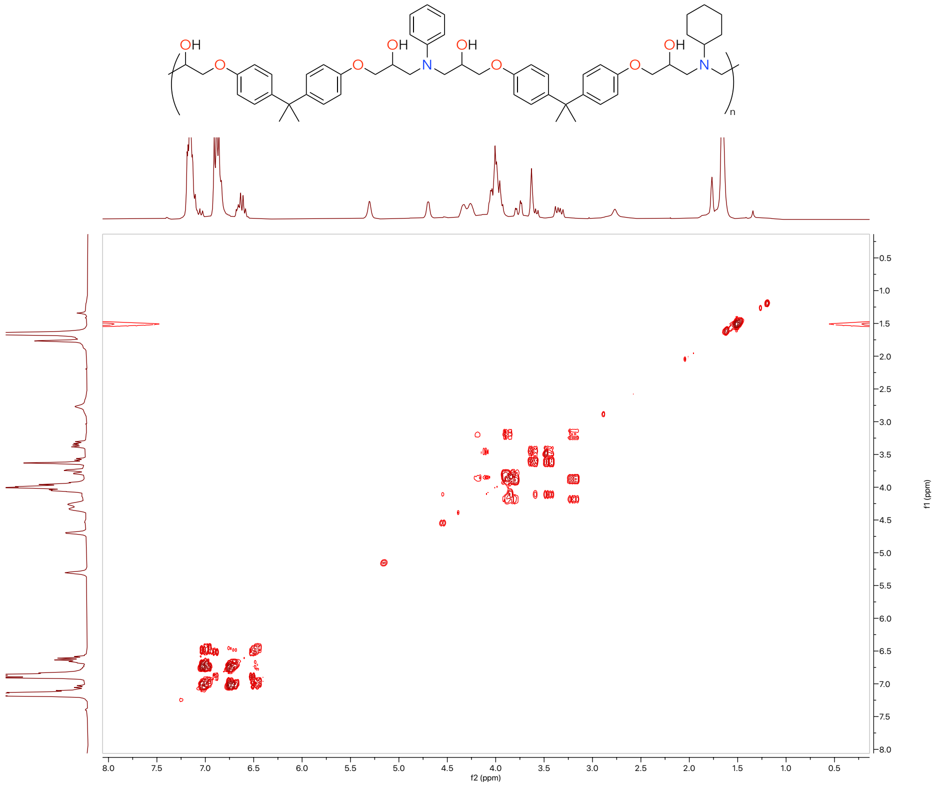

Figure S49. COSY NMR spectrum (300 MHz, THF-d$_8$, 298 K) of poly-1-(cyclohexyl(ethyl)amino)-3-(4-(2-(4-(2-hydroxy-3-((2-hydroxy-3-(4-(2-(4-(2-hydroxypropoxy)phenyl)propan-2-yl)phenoxy)propyl)(phenyl)amino)propoxy)phenyl)propan-2-yl)phenoxy)propan-2-ol, as synthesized with 0.1 eq. of cyclohexylamine and 0.9 eq. of aniline (**11**).

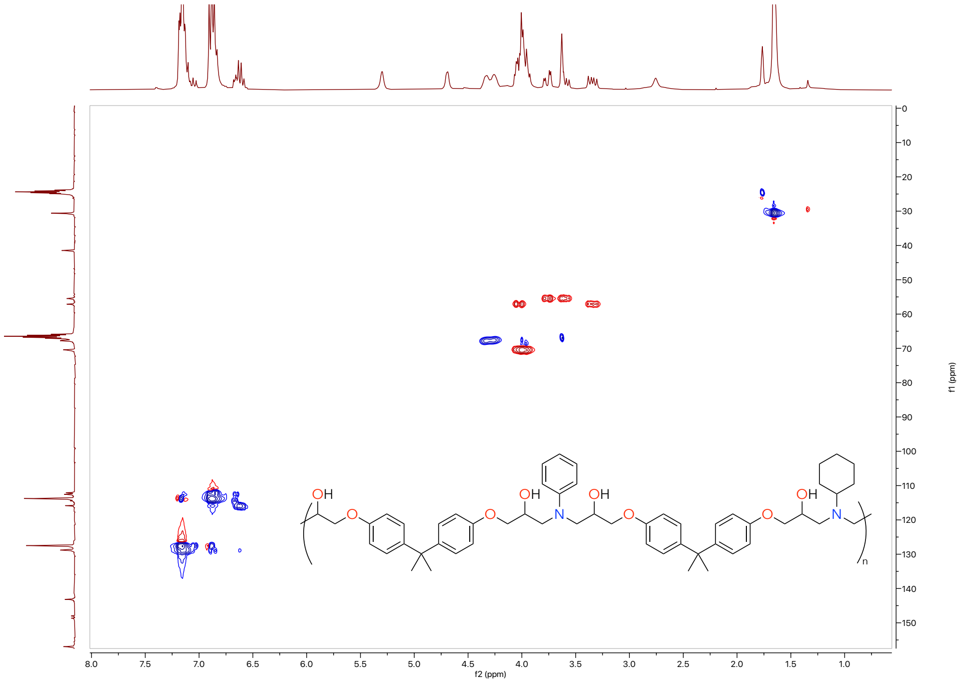

Figure S50. HSQC NMR spectrum (300 MHz, THF-d8, 298 K) of poly-1-(cyclohexyl(ethyl)amino)-3-(4-(2-(4-(2-hydroxy-3-((2-hydroxy-3-(4-(2-(4-(2-hydroxypropoxy)phenyl)propan-2-yl)phenoxy)propyl)(phenyl)amino)propoxy)phenyl)propan-2-yl)phenoxy)propan-2-ol, as synthesized with 0.1 eq. of cyclohexylamine and 0.9 eq. of aniline (**11**).

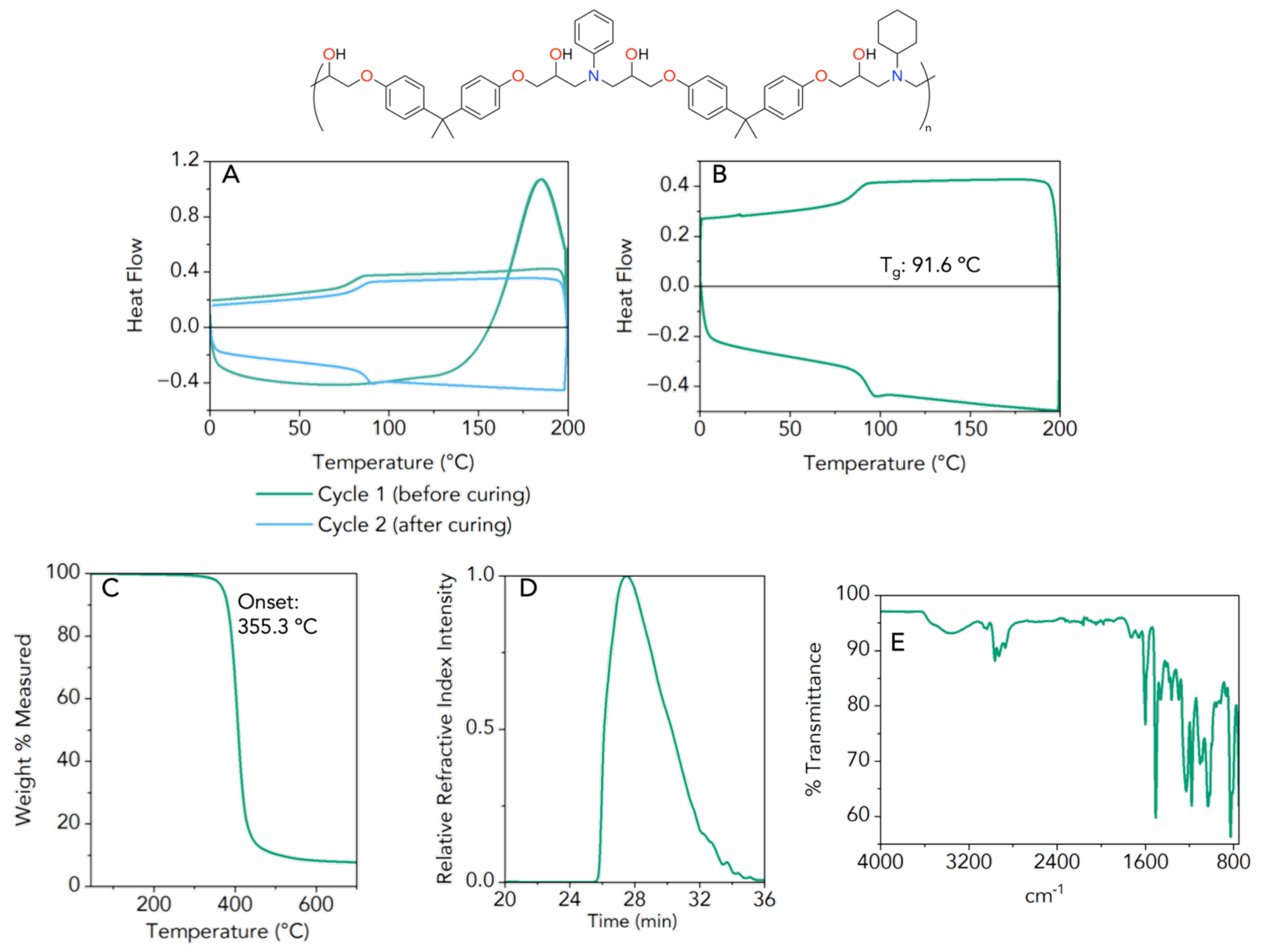

Figure S51. (**A**) DSC polymerization data before the synthesis of poly-1-(cyclohexyl(ethyl)amino)-3-(4-(2-(4-(2-hydroxy-3-((2-hydroxy-3-(4-(2-(4-(2-hydroxypropoxy)phenyl)propan-2-yl)phenoxy)propyl)(phenyl)amino)propoxy)phenyl)propan-2-yl)phenoxy)propan-2-ol, as synthesized with 0.1 eq. of cyclohexylamine and 0.9 eq. of aniline (**11**). (**B**) DSC analysis of **11**. (**C**) TGA analysis of **11**. (**D**) GPC analysis of **11**. (**E**) IR analysis of **11**.

Table S9. Molecular weight data for all thermoplastics (**7**-**11**), as determined via gel permeation chromatography. General GPC methods are noted in general procedures above. Note that for polymers **7**, **10**, and **11**, these data reflect the soluble fraction of these thermoplastics. All molecular weights were determined via the 100 % mass recovery method. PDI in all cases refers to the polydispersity index of each polymer.

Entry	Substrate	M_n	PDI
1	**7**	27.70 kDa ± 0.66 %	2.15 ± 0.77 %
2	**8**	49.75 kDa ± 3.43 %	1.96 ± 6.45 %
3	**9**	6.56 kDa ± 7.68 %	2.80 ± 7.68 %
4	**10**	12.64 kDa ± 0.46 %	1.99 ± 0.51 %
5	**11**	14.80 kDa ± 4.97 %	2.01 ± 4.91 %

S7. Thermoplastic Solubility Studies

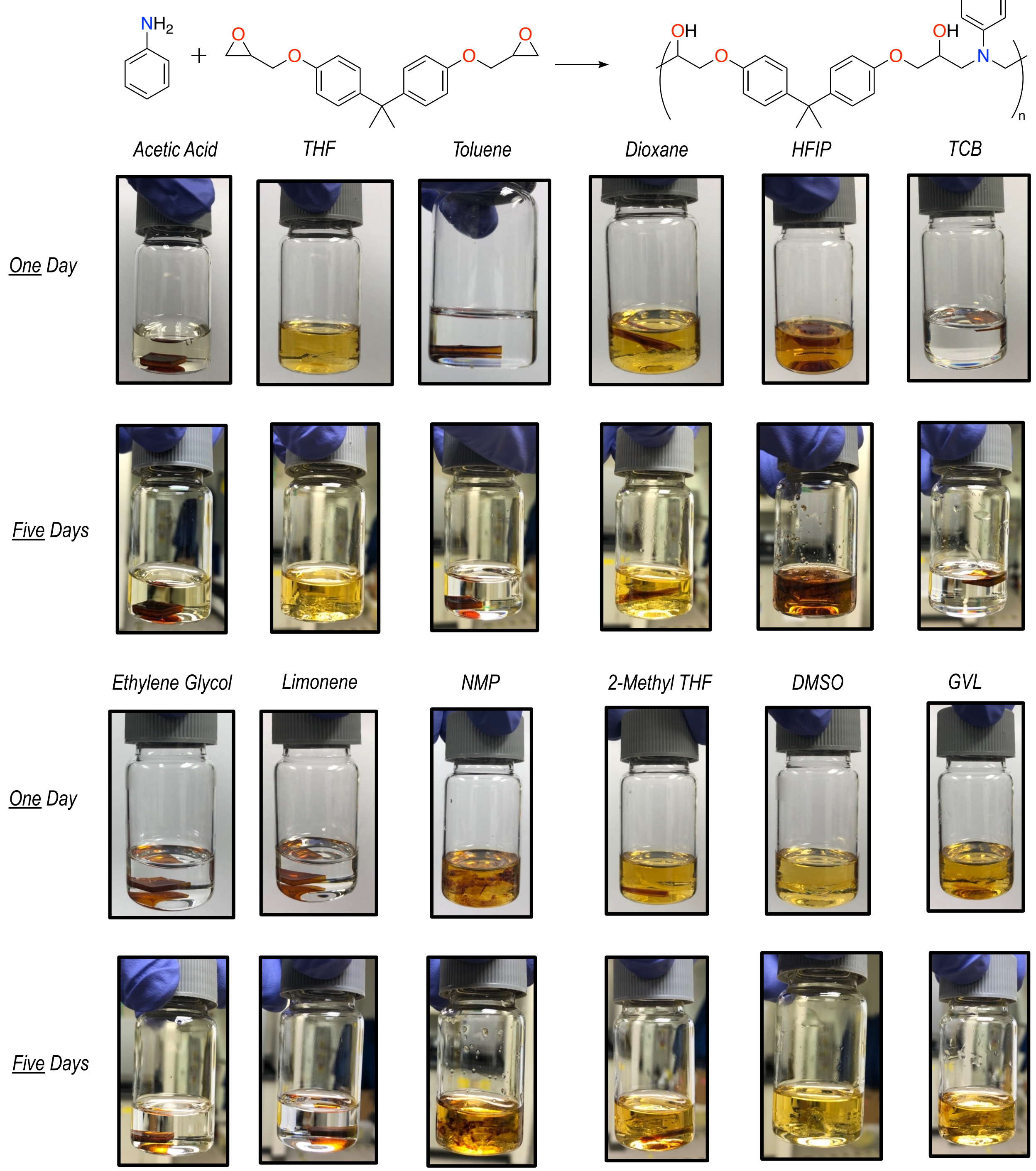

Figure S52. Solubility study results for poly-1-(ethyl(phenyl)amino)-3-(4-(2-(4-(2-hydroxypropoxy)phenyl)propan-2-yl)phenoxy)propan-2-ol (**7**). In this figure, THF represents tetrahydrofuran, HFIP represents hexafluoroisopropanol, TCB represents trichlorobenzene, NMP represents *N*-Methyl-2-pyrrolidone, DMSO represents dimethyl sulfoxide, and GVL represents γ-valerolactone.

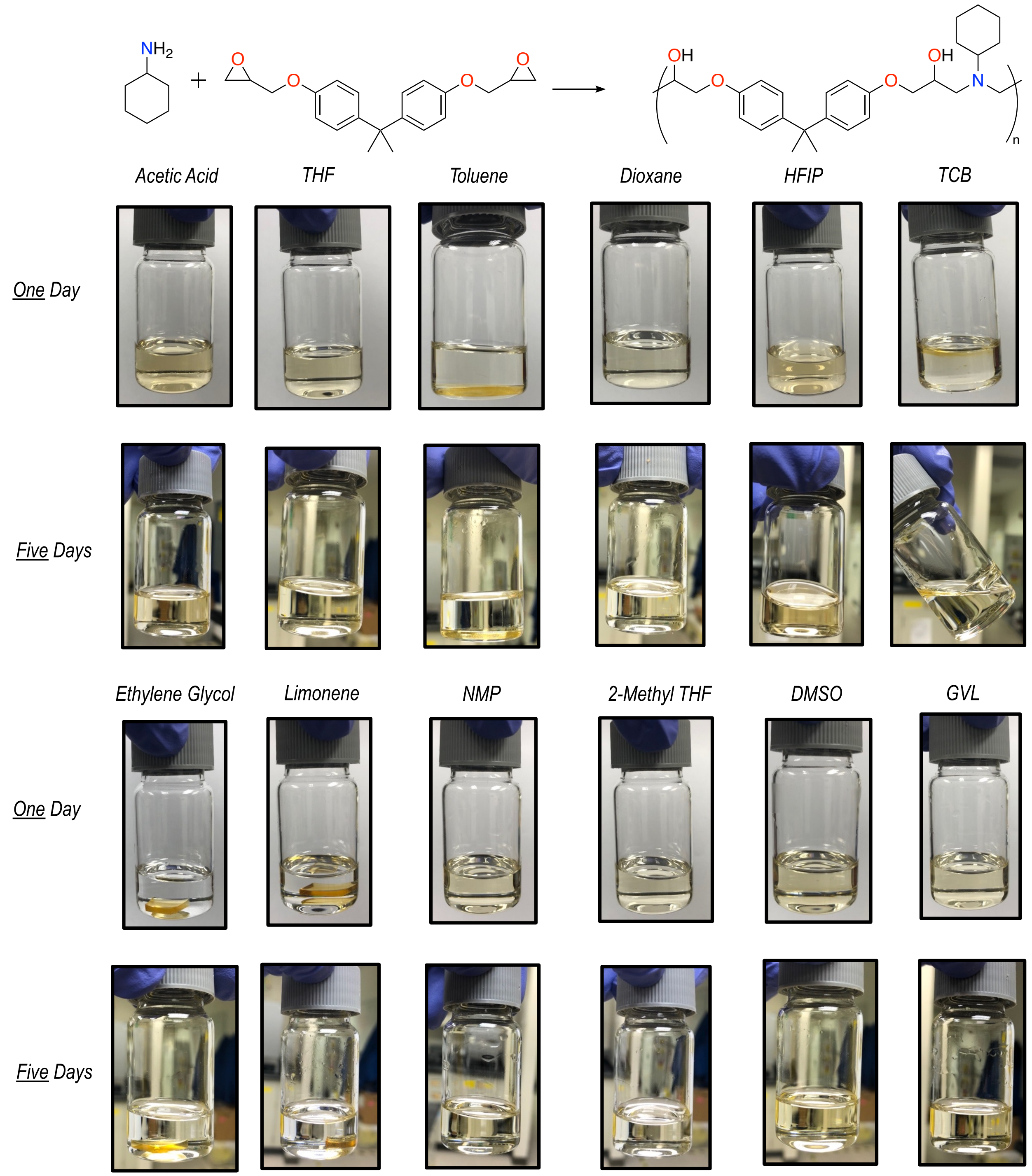

Figure S53. Solubility study results for poly-1-(cyclohexyl(ethyl)amino)-3-(4-(2-(4-(2-hydroxypropoxy)phenyl)propan-2-yl)phenoxy)propan-2-ol (**8**). In this figure, THF represents tetrahydrofuran, HFIP represents hexafluoroisopropanol, TCB represents trichlorobenzene, NMP represents *N*-Methyl-2-pyrrolidone, DMSO represents dimethyl sulfoxide, and GVL represents γ-valerolactone.

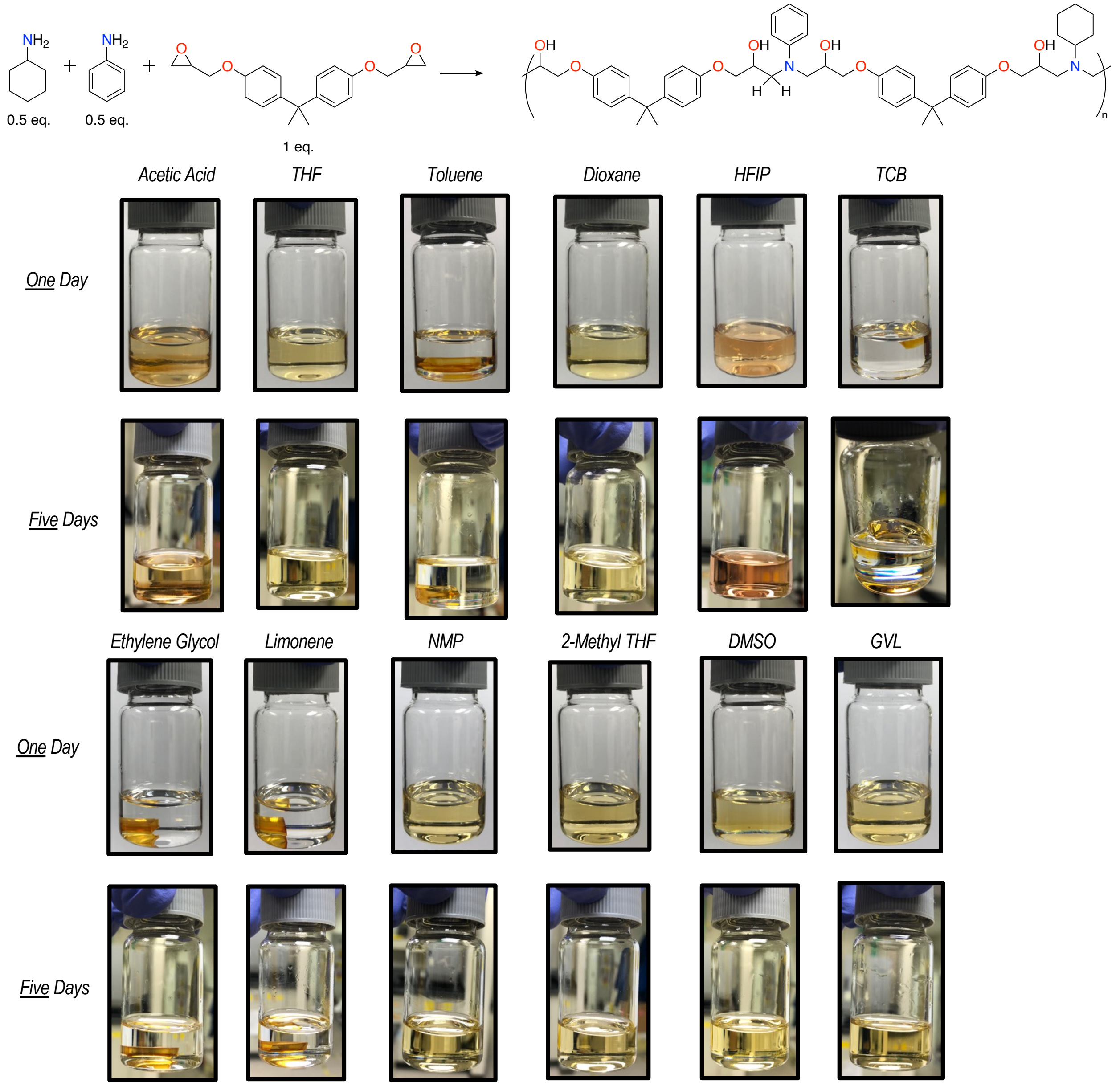

Figure S54. Solubility study results for poly-1-(cyclohexyl(ethyl)amino)-3-(4-(2-(4-(2-hydroxy-3-((2-hydroxy-3-(4-(2-(4-(2-hydroxypropoxy)phenyl)propan-2-yl)phenoxy)propyl)(phenyl)amino)propoxy)phenyl)propan-2-yl)phenoxy)propan-2-ol (**9**), as synthesized with 0.5 eq. of cyclohexylamine and 0.5 eq. of aniline. In this figure, THF represents tetrahydrofuran, HFIP represents hexafluoroisopropanol, TCB represents trichlorobenzene, NMP represents *N*-Methyl-2-pyrrolidone, DMSO represents dimethyl sulfoxide, and GVL represents γ-valerolactone.

S8. Base-Mediated Depolymerization Reactions of Model Thermoplastics in Microwave Vials

General procedures for thermoplastic deconstruction reactions – Microwave vials:
A single amine-cured epoxy thermoplastic cube (~200 mg, 0.5 mmol of monomer repeat units) and desired equivalents of base were weighed and directly added to a 20 mL Biotage microwave vial with a magnetic stir bar. Appropriate volumes of solvent(s) were added via a positive displacement pipette before sealing the reaction with a Biotage cap septum via an automatic crimper set to 55% tightness. The vial was then placed in a preheated heat block on a hot plate and reacted while stirring at 650 rpm. The vial was removed from heat after varying times. To neutralize the reaction, 3 mL of 2 M HCl in diethyl ether was added directly to the reaction vials via a positive displacement pipette. Reactions were then directly sampled with a 10uL positive displacement pipette for a 100x reaction dilution with HPLC grade acetone and direct GC-FID quantification of BPA. GC methods were discussed above in **Section S3**. Note that theoretical monomer content was calculated using the repeat unit weight for each polymer.

Thermoplastic deconstruction reactions and products:

Table S10. Yield data after comparing solvent amounts and ratios for thermoplastic deconstructions at 140 °C. Number values represent % yield values of BPA measured via GC-FID.

Entry	Substrate	Conditions	BPA
1	**7**	4 eq. KOtBu, 3 mL 1:1 Toluene:THF, 140 °C, 48h	29
2	**7**	4 eq. KOtBu, 4.5 mL 2:1 Toluene:THF, 140 °C, 48h	37
3	**8**	4 eq. KOtBu, 3 mL 1:1 Toluene:THF, 140 °C, 48h	37
4	**8**	4 eq. KOtBu, 4.5 mL 2:1 Toluene:THF, 140 °C, 48h	51
5	**9**	4 eq. KOtBu, 3 mL 1:1 Toluene:THF, 140 °C, 48h	54
6	**9**	4 eq. KOtBu, 4.5 mL 2:1 Toluene:THF, 140 °C, 48h	49

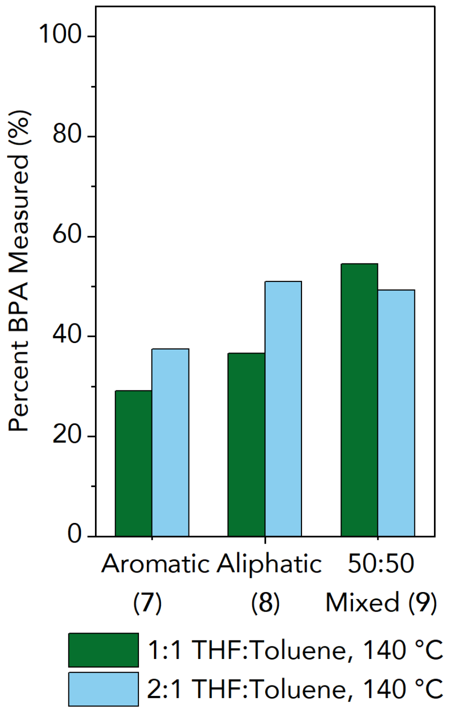

Figure S55. Yield data after comparing solvent amounts and ratios for thermoplastic deconstructions at 140 °C.

Table S11. Yield data after comparing solvent amounts and ratios for thermoplastic deconstructions at 160 °C. Number values represent % yield values of BPA measured via GC-FID.

Entry	Substrate	Conditions	BPA
1	**7**	4 eq. KOtBu, 3 mL 1:1 Toluene:THF, 160 °C, 48h	54
2	**7**	4 eq. KOtBu, 4.5 mL 2:1 Toluene:THF, 160 °C, 48h	71
3	**8**	4 eq. KOtBu, 3 mL 1:1 Toluene:THF, 160 °C, 48h	64
4	**8**	4 eq. KOtBu, 4.5 mL 2:1 Toluene:THF, 160 °C, 48h	65
5	**9**	4 eq. KOtBu, 3 mL 1:1 Toluene:THF, 160 °C, 48h	66
6	**9**	4 eq. KOtBu, 4.5 mL 2:1 Toluene:THF, 160 °C, 48h	59

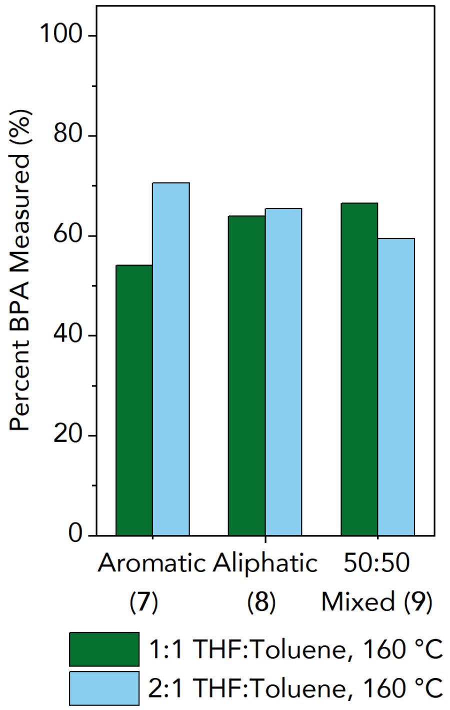

Figure S56. Yield data after comparing solvent amounts and ratios for thermoplastic deconstructions at 160 °C.

Table S12. Yield data after comparing reaction times for thermoplastic deconstructions at 140 °C. Number values represent % yield values of BPA measured via GC-FID.

Entry	Substrate	Conditions	BPA
1	**7**	4 eq. KOtBu, 3 mL 1:1 Toluene:THF, 140 °C, 24h	29
2	**7**	4 eq. KOtBu, 3 mL 1:1 Toluene:THF, 140 °C, 48h	33
3	**8**	4 eq. KOtBu, 3 mL 1:1 Toluene:THF, 140 °C, 24h	37
4	**8**	4 eq. KOtBu, 3 mL 1:1 Toluene:THF, 140 °C, 48h	43
5	**9**	4 eq. KOtBu, 3 mL 1:1 Toluene:THF, 140 °C, 24h	54
6	**9**	4 eq. KOtBu, 3 mL 1:1 Toluene:THF, 140 °C, 48h	54

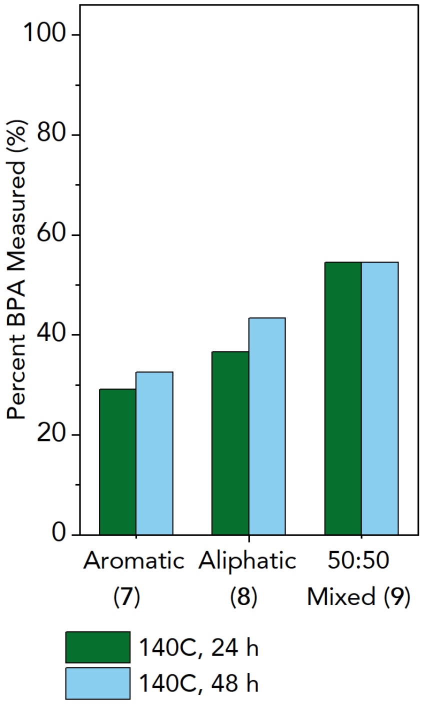

Figure S57. Yield data after comparing reaction times for thermoplastic deconstructions at 140 °C.

Table S13. Yield data after comparing reaction times for thermoplastic deconstructions at 160 °C. Number values represent % yield values of BPA measured via GC-FID.

Entry	Substrate	Conditions	BPA
1	**7**	4 eq. KOtBu, 3 mL 1:1 Toluene:THF, 160 °C, 24h	39
2	**7**	4 eq. KOtBu, 3 mL 1:1 Toluene:THF, 160 °C, 48h	54
3	**8**	4 eq. KOtBu, 3 mL 1:1 Toluene:THF, 160 °C, 24h	50
4	**8**	4 eq. KOtBu, 3 mL 1:1 Toluene:THF, 160 °C, 48h	64
5	**9**	4 eq. KOtBu, 3 mL 1:1 Toluene:THF, 160 °C, 24h	56
6	**9**	4 eq. KOtBu, 3 mL 1:1 Toluene:THF, 160 °C, 48h	66

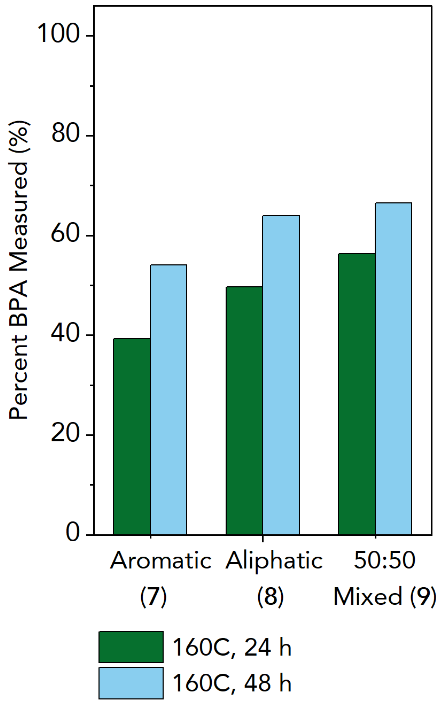

Figure S58. Yield data after comparing reaction times for thermoplastic deconstructions at 160 °C.

Table S14. Yield data after reactivity with cryomilled starting polymers vs. polymer cubes. Number values represent % yield values of BPA measured via GC-FID.

Entry	Substrate	Conditions	BPA
1	**7**	4 eq. KOtBu, 3 mL 1:1 Toluene:THF, 140 °C, 24h, **full polymer cube**	29
2	**7**	4 eq. KOtBu, 3 mL 1:1 Toluene:THF, 140 °C, 24h, **cryomilled polymer**	35
3	**8**	4 eq. KOtBu, 3 mL 1:1 Toluene:THF, 140 °C, 24h, **full polymer cube**	37
4	**8**	4 eq. KOtBu, 3 mL 1:1 Toluene:THF, 140 °C, 24h, **cryomilled polymer**	50
5	**9**	4 eq. KOtBu, 3 mL 1:1 Toluene:THF, 140 °C, 24h, **full polymer cube**	18
6	**9**	4 eq. KOtBu, 3 mL 1:1 Toluene:THF, 140 °C, 24h, **cryomilled polymer**	37

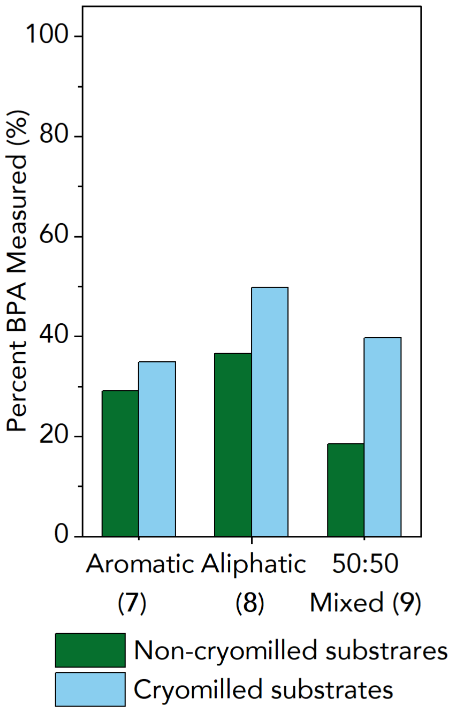

Figure S59. Yield data after reactivity with cryomilled starting polymers vs. polymer cubes.

Table S15. Yield data after comparing reaction temperatures for thermoplastic deconstructions. Number values represent % yield values of BPA measured via GC-FID.

Entry	Substrate	Conditions	BPA
1	**7**	4 eq. KOtBu, 3 mL 1:1 Toluene:THF, 140 °C, 24h	29
2	**7**	4 eq. KOtBu, 3 mL 1:1 Toluene:THF, 160 °C, 24h	39
3	**8**	4 eq. KOtBu, 3 mL 1:1 Toluene:THF, 140 °C, 24h	37
4	**8**	4 eq. KOtBu, 3 mL 1:1 Toluene:THF, 160 °C, 24h	50
5	**9**	4 eq. KOtBu, 3 mL 1:1 Toluene:THF, 140 °C, 24h	54
6	**9**	4 eq. KOtBu, 3 mL 1:1 Toluene:THF, 160 °C, 24h	56

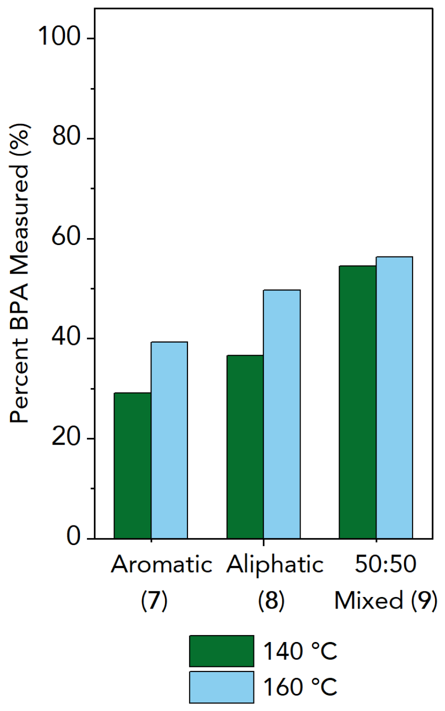

Figure S60. Yield data after comparing reaction temperatures for thermoplastic deconstructions.

Table S16. Yield data after comparing reaction temperatures of 160 °C vs. 180 °C for thermoplastic deconstructions. Number values represent % yield values of BPA measured via GC-FID.

Entry	Substrate	Conditions	BPA
1	**7**	4 eq. KOtBu, 4.5 mL 2:1 Toluene:Dioxane, 160 °C, 48h	72
2	**7**	4 eq. KOtBu, 4.5 mL 2:1 Toluene:Dioxane, 180 °C, 48h	66
3	**8**	4 eq. KOtBu, 4.5 mL 2:1 Toluene:Dioxane, 160 °C, 48h	99
4	**8**	4 eq. KOtBu, 4.5 mL 2:1 Toluene:Dioxane, 180 °C, 48h	87
5	**9**	4 eq. KOtBu, 4.5 mL 2:1 Toluene:Dioxane, 160 °C, 48h	90
6	**9**	4 eq. KOtBu, 4.5 mL 2:1 Toluene:Dioxane, 180 °C, 48h	94

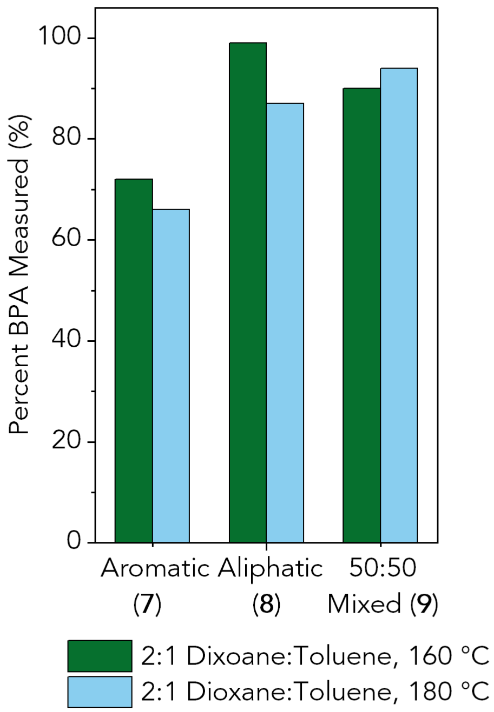

Figure S61. Yield data after comparing reaction temperatures of 160 °C vs. 180 °C for thermoplastic deconstructions.

Table S17. Yield data from **Figure 6A** in the main text of this paper, comparing BPA yields from optimized model compound conditions vs. optimized thermoplastic conditions. SD in this table refers to standard deviations. Number values represent % yield values of BPA measured via GC-FID.

Entry	Substrate	Conditions	BPA
1	**7**	4 eq. KOtBu, 3 mL 1:1 Toluene:THF, 140 °C, 24h	29
2	**7**	4 eq. KOtBu, 3 mL 1:1 Toluene:THF, 140 °C, 24h	36
3	**7**	4 eq. KOtBu, 3 mL 1:1 Toluene:THF, 140 °C, 24h	38
		Average =	34
		SD =	5
4	**8**	4 eq. KOtBu, 3 mL 1:1 Toluene:THF, 140 °C, 24h	37
5	**8**	4 eq. KOtBu, 3 mL 1:1 Toluene:THF, 140 °C, 24h	43
6	**8**	4 eq. KOtBu, 3 mL 1:1 Toluene:THF, 140 °C, 24h	40
		Average =	40
		SD =	3
7	**9**	4 eq. KOtBu, 3 mL 1:1 Toluene:THF, 140 °C, 24h	54
8	**9**	4 eq. KOtBu, 3 mL 1:1 Toluene:THF, 140 °C, 24h	47
9	**9**	4 eq. KOtBu, 3 mL 1:1 Toluene:THF, 140 °C, 24h	48
		Average =	50
		SD =	4

10	**7**	4 eq. KOtBu, 4.5 mL 2:1 Toluene:THF, 160 °C, 48h	71
11	**7**	4 eq. KOtBu, 4.5 mL 2:1 Toluene:THF, 160 °C, 48h	64
12	**7**	4 eq. KOtBu, 4.5 mL 2:1 Toluene:THF, 160 °C, 48h	66
13	**7**	4 eq. KOtBu, 4.5 mL 2:1 Toluene:THF, 160 °C, 48h	57
		Average =	64
		SD =	5
14	**8**	4 eq. KOtBu, 4.5 mL 2:1 Toluene:THF, 160 °C, 48h	65
15	**8**	4 eq. KOtBu, 4.5 mL 2:1 Toluene:THF, 160 °C, 48h	47
16	**8**	4 eq. KOtBu, 4.5 mL 2:1 Toluene:THF, 160 °C, 48h	75
		Average =	62
		SD =	14
17	**9**	4 eq. KOtBu, 4.5 mL 2:1 Toluene:THF, 160 °C, 48h	62
18	**9**	4 eq. KOtBu, 4.5 mL 2:1 Toluene:THF, 160 °C, 48h	59
19	**9**	5 eq. KOtBu, 4.5 mL 2:1 Toluene:THF, 160 °C, 48h	73
		Average =	65
		SD =	7
20	**7**	4 eq. KOtBu, 4.5 mL 2:1 Toluene:Dioxane, 160 °C, 48h	74
21	**7**	4 eq. KOtBu, 4.5 mL 2:1 Toluene:Dioxane, 160 °C, 48h	87
22	**7**	4 eq. KOtBu, 4.5 mL 2:1 Toluene:Dioxane, 160 °C, 48h	54
		Average =	72
		SD =	16
23	**8**	4 eq. KOtBu, 4.5 mL 2:1 Toluene:Dioxane, 160 °C, 48h	102
24	**8**	4 eq. KOtBu, 4.5 mL 2:1 Toluene:Dioxane, 160 °C, 48h	91
25	**8**	4 eq. KOtBu, 4.5 mL 2:1 Toluene:Dioxane, 160 °C, 48h	103
		Average =	99
		SD =	7
26	**9**	4 eq. KOtBu, 4.5 mL 2:1 Toluene:Dioxane, 160 °C, 48h	94
27	**9**	4 eq. KOtBu, 4.5 mL 2:1 Toluene:Dioxane, 160 °C, 48h	92
28	**9**	4 eq. KOtBu, 4.5 mL 2:1 Toluene:Dioxane, 160 °C, 48h	84
		Average =	90
		SD =	5

Table S18. Yield data from **Figure 6B** in the main text of this paper, comparing BPA yields from optimized model compound conditions vs. optimized thermoplastic conditions. SD in this table refers to standard deviations. Number values represent % yield values of BPA measured via GC-FID.

Entry	Substrate	Conditions	BPA	Aniline
1	**7**	4 eq. KOtBu, 4.5 mL 2:1 Toluene:Dioxane, 160 °C, 48h	74	62
2	**7**	4 eq. KOtBu, 4.5 mL 2:1 Toluene:Dioxane, 160 °C, 48h	87	57
3	**7**	4 eq. KOtBu, 4.5 mL 2:1 Toluene:Dioxane, 160 °C, 48h	54	54
		Average =	72	58
		SD =	16	4
4	**9**	4 eq. KOtBu, 4.5 mL 2:1 Toluene:Dioxane, 160 °C, 48h	94	48
5	**9**	4 eq. KOtBu, 4.5 mL 2:1 Toluene:Dioxane, 160 °C, 48h	92	33
6	**9**	4 eq. KOtBu, 4.5 mL 2:1 Toluene:Dioxane, 160 °C, 48h	84	33
		Average =	90	38
		SD =	5	9

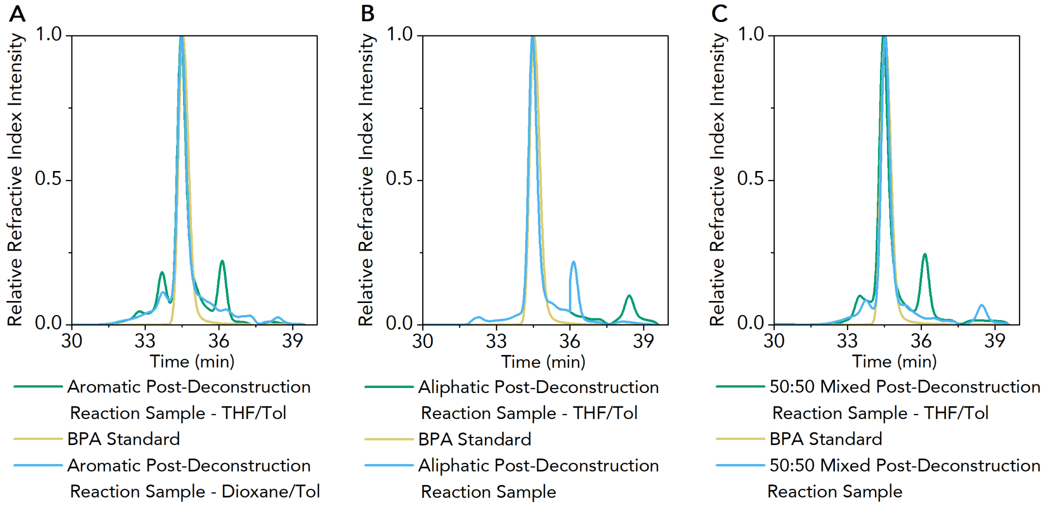

Figure S62. Comparative GPC data between optimized reactions in toluene/THF (teal) and toluene/dioxane (blue) as solvent mixtures for deconstruction of **7**–**9**.

S9. Thermoset Syntheses and Characterization

Synthesis of of 1-(cyclohexyl(-3-(4-(2-(4-(-3-((3-((dimethylamino)methyl)-3,5,5-trimethylcyclohexyl)(ethyl)amino)-2-hydroxypropoxy)phenyl)propan-2-yl)phenoxy)-2-hydroxypropyl)amino)-3-(4-(2-(4-(2-hydroxypropoxy)phenyl)propan-2-yl)phenoxy)propan-2-ol (12): Prepared by combining equal mol ratios of cyclohexylamine (2.91 g, 0.029 mol) and isophorone diamine (IPDA, 2.50 g, 0.016 mol) with one equivalent of BADGE. Monomers were combined by first melting the desired quantity of BADGE (20.00g, 0.059 mol), adding amine substrates, and homogenizing in a spin mixer cup at 2000 rpm for 10 seconds, and then 3000 rpm for 1 minute and 50 seconds. The resultant solution was added over a scale to 1x1x1 cm cubes in a

silicone sheet at desired weights. These polymer cubes were polymerized in a ventilated oven at the following cure schedule:
80 °C for 1 hr; 1.5 °C /min ramp to 100 °C; 100 °C for 8 hr; 1.5 °C /min ramp to 120 °C; 120 °C for 2 hr; 1.5 °C /min ramp to 130 °C; 130 °C for 4 hr.

Polymer cubes were then directly characterized and used in deconstruction reactions without purification.

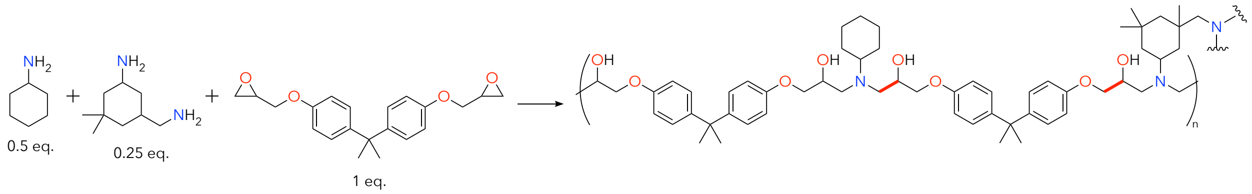

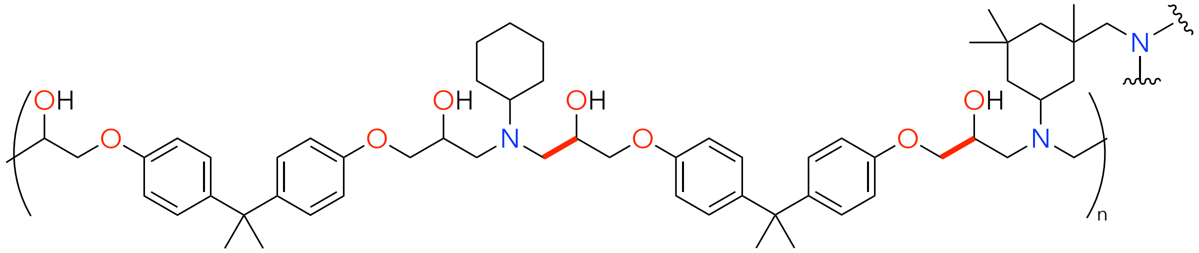

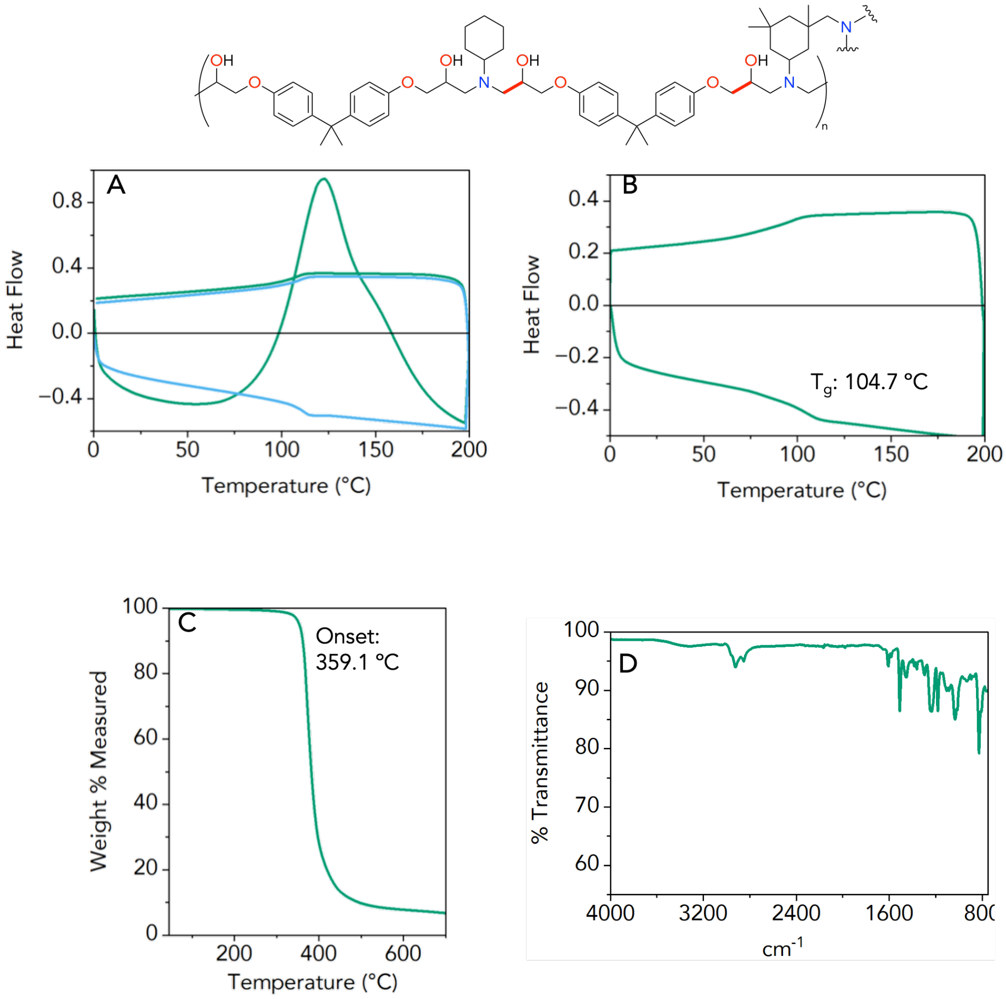

Figure S63. (**A**) DSC curing data before the synthesis of 1-(cyclohexyl(-3-(4-(2-(4-(-3-((3-((dimethylamino)methyl)-3,5,5-trimethylcyclohexyl)(ethyl)amino)-2-hydroxypropoxy)phenyl)propan-2-yl)phenoxy)-2-hydroxypropyl)amino)-3-(4-(2-(4-(2-hydroxypropoxy)phenyl)propan-2-yl)phenoxy)propan-2-ol (**12**). (**B**) DSC analysis of **12**. (**C**) TGA analysis of **12**. (**D**) IR analysis of **12**.

Thermoset **13** was prepared according to instructions in the following datasheet:

https://www.metyx.com/wp-content/uploads/PDF_Files/Hexion/TDS/TDS%20RIMH%20137.pdf

Specifically, curing of **13** occurred at 80 °C for five hours.

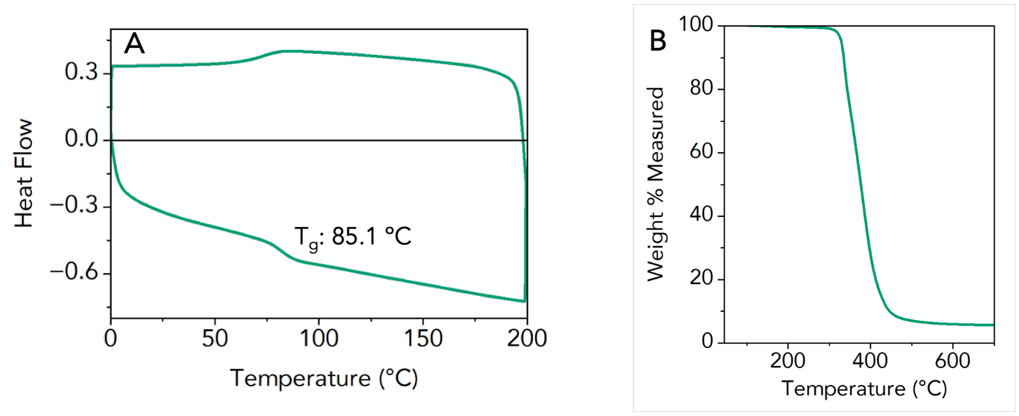

Figure S64. (**A**) DSC analysis of thermoset resin **13**. (**B**) TGA data for resin **13**.

S10. Thermoset Swelling Studies

For these experiment, a polymer cube of either **12** or **13** was added to a microwave vial with 3 mL of HPLC THF or Dioxane and a rare earth stir bar. These vials have a maximum pressure rating of 300 psi. This mixture was heated to 200 °C for one hour. Before TGA analyses, cubes were dried in a vacuum oven at 40 °C for one hour.

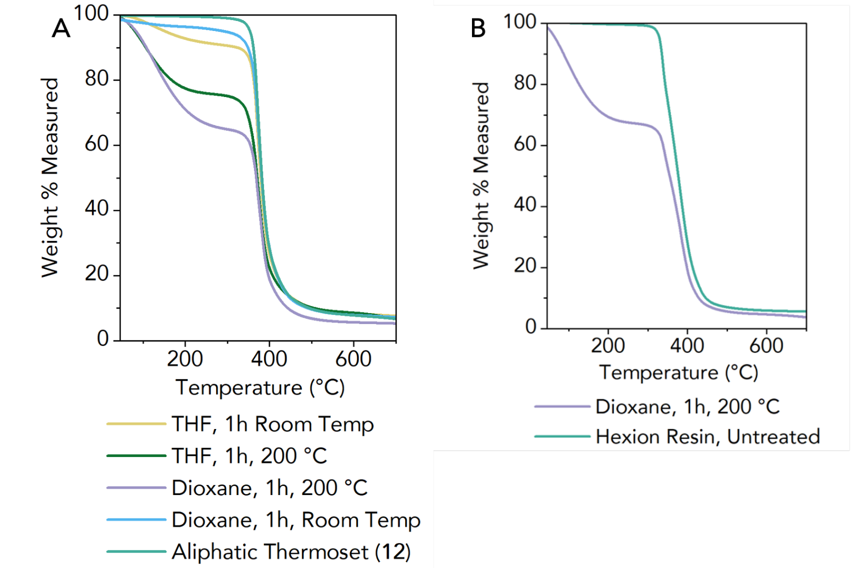

Figure S65. (**A**) TGA data before and after swelling of 1-(cyclohexyl(-3-(4-(2-(4-(-3-((3-((dimethylamino)methyl)-3,5,5-trimethylcyclohexyl)(ethyl)amino)-2-hydroxypropoxy)phenyl)propan-2-yl)phenoxy)-2-hydroxypropyl)amino)-3-(4-(2-(4-(2-hydroxypropoxy)phenyl)propan-2-yl)phenoxy)propan-2-ol (**12**). (**B**) TGA data before and after swelling of resin **13**.

S11. Thermoset Deconstruction Reactions

General procedures for thermoset deconstruction reactions – Microwave vials (GP3):

Cubes of **12** or of prepared were pretreated via swelling in 3 mL THF for 1 h at 200 C in a microwave (see **S8** above). The cube and all THF was then transferred to a 20 mL Biotage microwave vial, before adding 1.5 mL of toluene and 4eq. of KO*t*Bu with a magnetic stir bar. The reaction was sealed with a Biotage cap septum via an automatic crimper set to 55% tightness. The vial was then placed in a preheated heat block on a hot plate and reacted while stirring at 650 rpm. The vial was removed from heat after 48 h. To neutralize the reaction, 3 mL of 2M HCl in diethyl ether was added directly to the reaction vials via a positive displacement pipette. Reactions were then directly sampled with a 10uL positive displacement pipette to a 100x reaction dilution with HPLC grade acetone for direct GC-FID quantification of reaction products.

Table S19. Yield data from **Figure 8** in the main text of this paper, comparing BPA yields from optimized model compound conditions vs. optimized thermoplastic conditions. SD in this table refers to standard deviations. Number values represent % yield values of BPA measured via GC-FID.

Entry	Substrate	Conditions	BPA
1	**12**	4 eq. KOtBu, 4.5 mL 2:1 Toluene:Dioxane, 160 °C, 48h	81
2	**12**	4 eq. KOtBu, 4.5 mL 2:1 Toluene:Dioxane, 160 °C, 48h	73
3	**12**	4 eq. KOtBu, 4.5 mL 2:1 Toluene:Dioxane, 160 °C, 48h	60
		Average =	71
		SD =	11
